# Increased levels of acidic free-*N*-glycans, including multi-antennary and fucosylated structures, in the urine of cancer patients

**DOI:** 10.1371/journal.pone.0266927

**Published:** 2022-04-12

**Authors:** Ken Hanzawa, Miki Tanaka-Okamoto, Hiroko Murakami, Noriko Suzuki, Mikio Mukai, Hidenori Takahashi, Takeshi Omori, Kenji Ikezawa, Kazuyoshi Ohkawa, Masayuki Ohue, Shunji Natsuka, Yasuhide Miyamoto

**Affiliations:** 1 Department of Molecular Biology, Osaka International Cancer Institute, Chuo-ku, Osaka, Japan; 2 Graduate School of Science and Technology, Niigata University, Nishi-ku, Niigata, Japan; 3 Department of Medical Checkup, Osaka International Cancer Institute, Chuo-ku, Osaka, Japan; 4 Department of Gastroenterological Surgery, Osaka International Cancer Institute, Chuo-ku, Osaka, Japan; 5 Department of Hepatobiliary and Pancreatic Oncology, Osaka International Cancer Institute, Chuo-ku, Osaka, Japan; Fisheries and Oceans Canada, CANADA

## Abstract

We recently reported increased levels of urinary free-glycans in some cancer patients. Here, we focused on cancer related alterations in the levels of high molecular weight free-glycans. The rationale for this study was that branching, elongation, fucosylation and sialylation, which lead to increases in the molecular weight of glycans, are known to be up-regulated in cancer. Urine samples from patients with gastric cancer, pancreatic cancer, cholangiocarcinoma and colorectal cancer and normal controls were analyzed. The extracted free-glycans were fluorescently labeled with 2-aminopyridine and analyzed by multi-step liquid chromatography. Comparison of the glycan profiles revealed increased levels of glycans in some cancer patients. Structural analysis of the glycans was carried out by performing chromatography and mass spectrometry together with enzymatic or chemical treatments. To compare glycan levels between samples with high sensitivity and selectivity, simultaneous measurements by reversed-phase liquid chromatography-selected ion monitoring of mass spectrometry were also performed. As a result, three lactose-core glycans and 78 free-*N*-glycans (one phosphorylated oligomannose-type, four sialylated hybrid-type and 73 bi-, tri- and tetra-antennary complex-type structures) were identified. Among them, glycans with α1,3-fucosylation ((+/− sialyl) Lewis X), triply α2,6-sialylated tri-antennary structures and/or a (Man3)GlcNAc1-core displayed elevated levels in cancer patients. However, simple α2,3-sialylation and α1,6-core-fucosylation did not appear to contribute to the observed increase in the level of glycans. Interestingly, one tri-antennary free-*N*-glycan that showed remarkable elevation in some cancer patients contained a unique Glcβ1-4GlcNAc-core instead of the common GlcNAc2-core at the reducing end. This study provides further insights into free-glycans as potential tumor markers and their processing pathways in cancer.

## Introduction

Elevated levels of glycans and glycoproteins, such as CA19-9, STN, SLX, CEA, CA15-3 and AFP, are routinely used as tumor markers in clinical practice [1–3]. Nonetheless, to improve diagnosis, novel tumor markers are sought that are effective in those patients who are negative for existing markers. Studies of glycan profiles of glycoproteins have revealed alterations associated with cancer [4,5]. However, few studies have analyzed cancer associated changes in the levels of free-glycans. These free-glycans comprise structures in which the reducing ends are not covalently linked to other molecules. Abnormal accumulation of sialyl free-*N*-glycans has been reported in the cytoplasm of gastric cancer cell lines [6] and in cancer tissues from pancreatic and prostate cancer patients [7,8]. Free-glycans excreted in urine are particularly attractive candidate disease markers for large scale screening because they can be measured in a non-invasive manner.

Urinary free-glycan profiles have been reported to reflect alterations in the metabolic pathways of glycosylation in some diseases, especially in patients with oligosaccharidosis, a subgroup of lysosomal storage diseases [9,10]. The levels of some free-glycans were also increased in healthy individuals during pregnancy and lactation [11,12]. Moreover, increased urinary levels of sialyllactose and sialyl LacNAc have been reported in some inflammatory diseases [13,14] as well as cancer [15,16]. Based on these reports, we recently performed profiling of major urinary acidic free-glycans and reported that some sialylated, fucosylated, and/or sulfated free-glycans were elevated in cancer patients [17]. Our previous study was carried out using a method optimized for free-glycans ranging from disaccharides to simple sialylated bi-antennary free-*N*-glycans but did not include higher molecular weight glycans. Urinary free-glycans may be present in more than just low molecular weight degraded fragments. In some patients with lysosomal diseases including GM1 gangliosidosis, galactosialidosis, and mucolipidosis, larger multiantennary and/or elongated free-*N*-glycans have been detected [18–20]. Additionally, it is well established that glycans with branched, elongated, sialylated, and/or fucosylated structures are up-regulated in cancer [21]. Based on these findings, we reasoned that larger free-glycans may be present at elevated levels in the urine of cancer patients. In this study, we investigated urinary free-glycans, focusing on fractions containing acidic bi- to tetra-antennary free-*N*-glycans, in order to identify cancer-specific glycans that may act as novel tumor marker candidates. Chromatography and mass spectrometry-based approaches revealed that some free-glycans, especially tri-/tetra-antennary free-*N*-glycans with α1,3-fucosylation or triple α2,6-sialylation, tend to be present at elevated levels in cancer patients. Interestingly, one of the glycans that showed elevated levels in cancer patients included a tri-antennary free-*N*-glycan variation with a unique core of Glcβ1-4GlcNAc instead of the common core GlcNAc_2_.

## Materials and methods

### Urine samples

Urine samples of patients with gastric cancer (n = 12, male 7, female 5, mean age 66.4 years), pancreatic cancer (n = 10, male 6, female 4, mean age 62.7 years), cholangiocarcinoma (n = 4, male 2, female 2, mean age 68.0) and colorectal cancer (n = 15, male 4, female 11, mean age 63.3 years) were obtained from Osaka International Cancer Institute. The clinical features of the individuals examined in this study are summarized in Table 1. The patients were numbered G1–G12 for gastric cancer patients, and P1–P10 for pancreatic cancer patients, B1–B4 for cholangiocarcinoma (“B” is derived from bile duct) and C1–C15 for colorectal cancer patients. Urine samples of normal controls, numbered as N1–N21 (n = 21, male 15, female 6, mean age 63.2 years) were obtained from cancer-free volunteers. Except for colorectal cancer patients, these cancer patients and normal controls are identical to those used in our previous analysis of free glycans in urine [17]. This study was approved by the Local Ethics Committee of Osaka International Cancer Institute. Informed written consent was obtained from each patient and volunteer.

**Table 1:** Clinical information of the normal controls and patients.

Case No. ^a^	Sex	Age	Stage	CA19-9 (U/mL)^b^	CEA (ng/mL)^c^	ABO blood group	Urine Creatinine (mg/dL)	BUN^d^ (mg/dL)	Serum Creatinine (mg/dL)^e^
N1	M	64	nd^f^	<2	1.9	O	61.6	13	1.14
N2	M	69	nd	7	1.5	O	175.0	18	0.80
N3	M	69	nd	2	1.8	A	59.8	11	0.86
N4	M	77	nd	4	1.2	A	190.6	22	0.82
N5	M	68	nd	2	3.8	O	116.7	15	1.02
N6	F	53	nd	3	2.1	AB	140.1	13	0.73
N7	F	70	nd	3	1.1	O	77.9	16	0.63
N8	M	75	nd	14	2.1	A	84.0	20	1.20
N9	M	81	nd	**2**	1.2	A	83.1	18	1.00
N10	M	50	nd	12	2.9	B	84.1	15	0.93
N11	F	75	nd	2	2.8	B	58.8	14	0.84
N12	M	69	nd	4	1.2	A	105.1	29	1.51
N13	F	33	nd	5	2.1	A	72.9	12	0.66
N14	F	56	nd	2	1.4	O	115.8	13	0.48
N15	M	81	nd	4	2.4	AB	80.6	16	0.85
N16	M	50	nd	8	1.5	A	281.4	15	1.01
N17	M	71	nd	12	3.2	A	43.8	15	0.71
N18	M	42	nd	3	0.8	A	94.2	21	0.90
N19	F	61	nd	5	1.4	B	77.2	14	0.60
N20	M	66	nd	4	2.5	A	126.9	17	0.83
N21	M	48	nd	3	0.9	A	148.4	12	0.74
G1	F	61	IV	4	1.1	B	32.1	11	0.42
G2	F	65	IV	67	1.0	A	384.7	11	0.69
G3	M	65	IV	206	60.0	B	115.3	19	0.79
G4	M	78	IV	568	6.2	AB	160.6	13	0.82
G5	F	70	IV	<2	1.2	B	319.4	14	0.63
G6	M	64	III	4	7.5	O	190.4	17	1.04
G7	M	72	IV	76	1.7	A	115.7	16	1.06
G8	M	74	IV	3	1.1	A	186.4	42	1.65
G9	M	59	III	1063	27.4	A	28.6	8	0.79
G10	F	67	IV	2	1.2	O	162.9	30	0.65
G11	F	62	IV	16	549.9	O	187.1	13	0.59
G12	M	60	III	1187	4.5	A	254.3	20	0.92
P1	M	48	IV	3311	3.7	A	69.4	12	0.72
P2	F	58	IV	>100000	560.2	B	180.5	12	0.66
P3	M	68	IV	>100000	220.5	A	439.5	18	0.86
P4	F	50	IV	16421	11.5	A	50.5	8	0.59
P5	F	66	IV	<2	3.0	B	159.5	14	0.69
P6	M	62	IV	46597	4.8	A	270.1	16	0.97
P7	M	72	IV	20124	13.1	A	51.5	15	0.48
P8	F	62	IV	371	7.3	A	116.2	29	0.77
P9	M	64	IV	>100000	162.9	O	187.7	15	0.86
P10	M	77	IV	>100000	42.0	A	59.7	15	0.56
B1	F	55	IV	29046	206.0	O	74.6	8	0.74
B2	M	65	IV	32678	1673.1	AB	363.8	19	1.43
B3	F	78	IV	>100000	164.4	O	45.4	15	0.93
B4	M	74	IV	81803	156.2	O	153.4	13	0.81
C1	F	70	IV	50	35.5	B	106.3	30	1.02
C2	F	51	IV	54545	1129.5	A	104.8	13	0.52
C3	F	45	IV	459	854.0	A	37.3	12	0.59
C4	M	70	IV	14038	126.4	A	198.2	20	1.12
C5	F	68	IV	307	85.1	AB	351.0	10	0.83
C6	F	71	IV	>100000	3472.6	A	182.6	15	0.53
C7	F	64	IV	4	19.1	A	34.7	10	0.61
C8	F	67	IV	4	1.6	A	96.1	6	0.57
C9	M	65	IV	294	13.1	O	201.6	15	0.73
C10	M	55	IV	1935	233.1	O	357.0	16	0.93
C11	F	63	IV	8	7.3	nd^f^	145.2	17	0.74
C12	F	73	IV	9	68.8	A	128.1	16	0.63
C13	F	61	IV	363	168.1	B	75.1	8	0.60
C14	M	55	IV	2	3.3	AB	269.5	20	0.89
C15	F	53	IV	328	132.8	O	34.5	8	0.42

a) N, G, P, B and C indicate normal control, patient with gastric cancer, pancreatic cancer, cholangiocarcinoma and colorectal cancer, respectively.

b) Cut-off value of CA19-9 and CEA are 37 U/mL and 5 ng/mL, respectively.

d) Normal range of BUN is 8.0–20.0 mg/dL.

e) Normal range of serum creatinine (mg/dL) is 0.65–1.07 for male (M) and 0.46–0.79 for female (F), respectively.

f) “nd” indicates “not determined”.

### Extraction and PA-labeling of free-glycans from urine

Extraction of free-glycans from urine were carried out as previously reported [17]. Briefly, urine was pretreated with Dowex 50W-X8 resin (H^+^-form, 200–400 mesh, FUJIFILM Wako Pure Chemical, Osaka, Japan), neutralized with sodium bicarbonate solution, and desalted with graphite carbon cartridge (InertSepGC 300 mg; GL Science, Tokyo, Japan). The reducing ends of the glycans were labeled by reductive amination with 2-aminopyridine [22,23]. Preparation of PA-glycans was performed as described previously [24,25].

### Liquid chromatography fractionation and analysis of PA-labeled urinary free-glycans

PA-glycans were fractionated on a HPLC system; either a Shimadzu LC20A or a Shimadzu LC10A (Shimadzu, Kyoto, Japan). Comparable separation of glycans was achieved using either of the two HPLC systems. PA-glycans were detected by a fluorescence detector connected to the HPLC, RF-10Axl (Shimadzu) or Waters 2475 (Waters, Milford, MA, USA). The separated glycans were collected in 1.5-mL tubes or 96-well plates by a 222XL liquid handler (Gilson, Middleton, WI, USA). Detailed settings for the HPLCs, including column specifications, temperatures, solvents, and gradient conditions are given in Table A (S1 File). Anion-exchange HPLC was performed on a TSKgel DEAE-5PW column (10 μm, 7.5 × 75 mm; Tosoh) as described in a previous study [26]. Normal phase (NP-, amide-HILIC-mode) HPLC was performed on a TSKgel Amide-80 (5 μm, 2 × 250 mm; Tosoh) [27].

Reversed phase (RP-) HPLC was performed at 35°C using a flow rate of 0.2 mL/min on a Shim-pack Scepter C18-120 column (3 μm, 2.1 × 150 mm; Shimadzu). The following solvents were used; solvent A: 9:1 (*v/v*) of water / 0.5 M acetic acid, adjusted to pH 4.0 with triethylamine, and solvent B: 7:2:1 (*v/v*) of water / acetonitrile / 0.5 M acetic acid adjusted to pH 4.0 with triethylamine. The elution time of each PA-glycan was converted to an *R* value by a modified reversed phase scale, as well as RP glucose units (RP-GU). The conversion of elution times to *R* values was based on a previous report, but with several modifications [28,29]. Eight (four pairs) standard PA-*N*-glycans #R-1 to R-8 were used as shown in Fig A (S2 File). The *R* values were determined so that the shift in position due to core-α1,6-fucose contributed equally. In the previous reversed phase scale, the *R* value at the time of sample injection was set to 0. The *R* value at the elution position of PA-Gal (#R-0) was set to 0. The elution time of #R-0 was subtracted from the elution time of each glycan, and the *R* value was calculated in the same way as described previously. In addition, the elution position of #R-8 was set to 70.

### Enzymatic digestions

Unless otherwise stated, enzymes supplied as a solution were used at 1/6–1/10 dilution in 1× NEB GlycoBuffer 1 (50 mM sodium acetate and 5 mM CaCl2 pH 5.5; New England Biolabs, Ipswich, MA, USA) and reactions were performed at 37°C overnight (i.e. for more than 16 h). *Streptococcus pneumoniae* β-*N*-acetylglucosaminidase, *Xanthomonas manihotis* β-galactosidase and *Streptomyces plicatus* β1,3/4/6-*N*-acetylhexosaminidase was from New England Biolabs, *S*. *pneumoniae* β-galactosidase was from Agilent Technologies (Santa Clara, CA, USA), bovine kidney α-fucosidase was from Sigma-Aldrich (St Louis, Mo, USA), and α1,3/4-fucosidase (*Streptomyces* sp. 142) was from Takara Bio (Shiga, Japan). To achieve substrate specificity, neuraminidase from *Salmonella typhimurium* (Takara Bio) was incubated for 2 hours to cleave non-reducing terminal α2,3-linkages or overnight to cleave non-reducing terminal α2,3/6-linkages [30]. Neuraminidase with broad specificity *Arthrobacter ureafaciens* (Nacalai, Kyoto, Japan) was used at a concentration of 2 mU/μL. Sodium phosphate (50 mM pH 6.8) was used as the buffer for β-mannosidase from *Cellulomonas fimi*, β-glucosidase from *Thermotoga maritima* and acetylxylanesterase from *Orpinomyces* sp. (Megazyme, Bray, Ireland). Jack bean α-mannosidase was used in 1× supplied buffer, 100 mM sodium acetate pH5.0, 2 mM ZnCl_2_ (Agilent Technologies). Alkaline phosphatase from *Escherichia coli* C75 was used in 1× supplied buffer, 50 mM Tris-HCl pH8.0, 1 mM MgCl_2_ (Takara Bio). The digested glycans were analyzed and desalted by RP-HPLC, and then subjected to NP-HPLC and mass spectrometry.

### Chemical treatments for linkage analysis of PA-glycans

Periodate oxidation and subsequent reduction were used for cleavage of C-C bonds between unsubstituted vicinal diols [31,32], and were performed as described previously [17]. To obtain supporting linkage information of sialic acids, sialic acid linkage-specific alkylamidation (SALSA) was performed using isopropylamine and methylamine [33]. The procedure was based on the previously described conditions for PA-glycans [34,35]. The treated glycans were analyzed by mass spectrometry.

### Standard glycans

The structures and further information of standard glycans are given in Table B (S1 File). The standard PA-labeled glycans were purchased from Takara Bio, Glyence (Aichi, Japan) or Seikagaku Corporation (Tokyo, Japan). Un-labeled glycans were from Sigma-Aldrich, Tokyo Chemical Industry (Tokyo, Japan), Dextra Laboratories (Reading, UK), Carbosynth (Compton, UK) and IsoSep AB (Stockholm, Sweden). The standard *N*-glycans were also prepared from glycoproteins by hydrazinolysis (anhydrous hydrazine, Nacalai) or enzymatic release using endo H and endo F3 (New England Biolabs). Sources of glycoproteins used were human blood γ-globulin, human plasma α1-acid glycoprotein, mouse serum, bovine serum (Sigma-Aldrich), sialylglycopeptide (Tokyo chemical industry) and bovine milk lactoferrin (FUJIFILM Wako Pure Chemical) [36–41]. The standard disaccharides, Glcβ1-4GlcNAc and GalNAcβ1-4GlcNAc (LacdiNAc), were enzymatically prepared. Briefly, it has been reported that bovine milk β-galactosyltransferase with α-lactalbumin (lactose synthase complex) also exhibits activities as a β1,4-glucosyltransferase and β1,4-*N*-acetylgalactosaminyltransferase [42,43]. The enzyme and α-lactalbumin were from Sigma-Aldrich. These disaccharides were synthesized by using UDP-glucose and UDP-GalNAc (Yamasa, Chiba, Japan) as donors and unlabeled GlcNAc (Tokyo Chemical Industry) as an acceptor. The unlabeled glycans were subjected to PA-labeling. As standards for reversed phase scale, #R-0 and R-1 were from Takara Bio, #R-2 was from the standard #Gn2-2 (GlcNAcβ1-4(Fucα1–6)GlcNAc; Tokyo Chemical Industry) by β-*N*-acetylglucosaminidase (*S*. *pneumoniae*) digestion. Glycans #R-3 and R-4 were from mouse serum *N*-glycans by digestions with neuraminidase (*A*. *ureafaciens*) and β1,4-glactosidase (*S*. *pneumoniae*). Glycans #R-5 to R-8 were from *N*-glycans of human blood γ-globulin with/without α-fucosidase (bovine kidney) and sialic acid labeling by ethylamine. For preparation of the standard disialylated agalacto-bi-antennary glycan with tetradeuterated (D_4_-) PA (#D_4_-std), *N*-glycans liberated from bovine serum were labeled by hexadeuterated aminopyridine (Cambridge Isotope Laboratories, Tewksbury, MA) [44], and the collected tetrasialyl fraction digested with neuraminidase (*S*. *typhimurium*) and β-galactosidase (*X*. *manihotis*).

### Mass spectrometry

Mass spectrometry for structural analysis was mainly performed on a LTQ XL linear ion trap mass spectrometer with a HESI-II probe (Thermo Scientific, San Jose, CA) connected to a LC20 HPLC system (Shimadzu). The HPLC was run at a flow rate of 50 μL/min and a temperature of 35°C. PA-glycans were trapped on a InertSustain AQ-C18 column (3 μm, 1 × 100 mm; GL Sciences) and eluted with 50% acetonitrile (Table B in S1 File). The column was equilibrated with 0.4% (*v/v*) formic acid to promote the formation of multiply protonated ions, or 5 mM acetic acid-triethylamine for forming of monovalent ions, which was suitable for labile periodate-treated glycans. The mass spectrometer was set to positive-ion mode. Sheath gas and auxiliary gas were set to 30 and 5 units. The following parameters were used for the formic acid solvent: spray voltage, 4 kV; ion source temperature, 250°C; ion transfer capillary temperature, 175°C; tube lens voltage, 80 V. The following parameters were used for the acetic acid-triethylamine solvent: spray voltage, 3 kV; ion source temperature, 250°C; ion transfer capillary temperature, 300°C; tube lens voltage, 125 V. MS^2^ experiments were performed in data-dependent mode or by specifying the value of the precursor ion. Data were recorded and analyzed using Xcalibur 4.0 software (Thermo Scientific).

SRM experiments and some of the structural analysis were performed on a 4500 QTrap hybrid triple quadrupole/linear ion trap mass spectrometer with a Turbo V ion source (AB SCIEX, Framingham, MA, USA) connected to a LC20A HPLC system (Shimadzu). The mass spectrometer was set to positive-ion mode, an ion spray voltage of 5 kV, curtain gas of 30 psi, nebulizer gas (GS1) of 65 psi, turbo gas (GS2) of 55 psi and an interface heater temperature of 500°C. Prior to SRM experiments, MS^2^ spectra of target glycans were acquired by Enhanced Product Ion (EPI) scan to obtain their fragmentation patterns in the Q TRAP instrument. For simultaneous scans, PA-glycans were separated by reversed-phase HPLC using MS-compatible solvents (Table A in S1 File). The following conditions were used: column, Shim-pack Scepter C18-120 (3 μm, 2.1 × 150 mm; Shimadzu), solvent A: 0.2% formic acid, solvent B: 0.2% (v/v) formic acid in 10% (v/v) acetonitrile, 40% (v/v) methanol, flow rate 0.25 mL/min, column temperature, 45°C. For stabilizing the ionization, 0.2 mL/min of acetonitrile was added post-column via a microvolume Tee-connector (Valco Instruments, Houston, TX, USA) by an additional LC-20AD pump [45–47]. Samples were measured in a randomized order. The D_4_-PA-labeled form of a standard glycan (#D4-std) was added to the samples (1 pmol/sample) prior to measurement to check the stability of the system. A mixture of a portion of all analyzed samples was prepared as a pooled quality control (QC) of the system and measured in intervals of 10 or 11 samples [48,49]. Data acquisition was performed by enabling scheduled MRM mode. The settings for sMRM, including Q1 and Q3 values, elution time and acquisition window, are shown in Table C in S1 File. The data were recorded by Analyst 1.7.2 software and analyzed by SCIEX OS 2.0.0 software (AB SCIEX). The peak area in the extracted ion chromatogram of each SRM transition was measured. For each PA glycan transition, an intensity of 600 was used as the LOQ (limit of quantification). As an exception, the LOQ was set to 200 for transitions with a weight setting of 5.0. For the data of the glycan levels obtained from SRM, Mann−Whitney *U* test was performed in GraphPad PRISM 6.0. Values lower than LOQ were set to 0. PCA was performed on the web-based platform, MetaboAnalyst version 5.0 [50,51]. The data of the glycan levels were autoscaled.

## Results

### Preparation and analysis of urinary free-glycans

**Fig 1:**
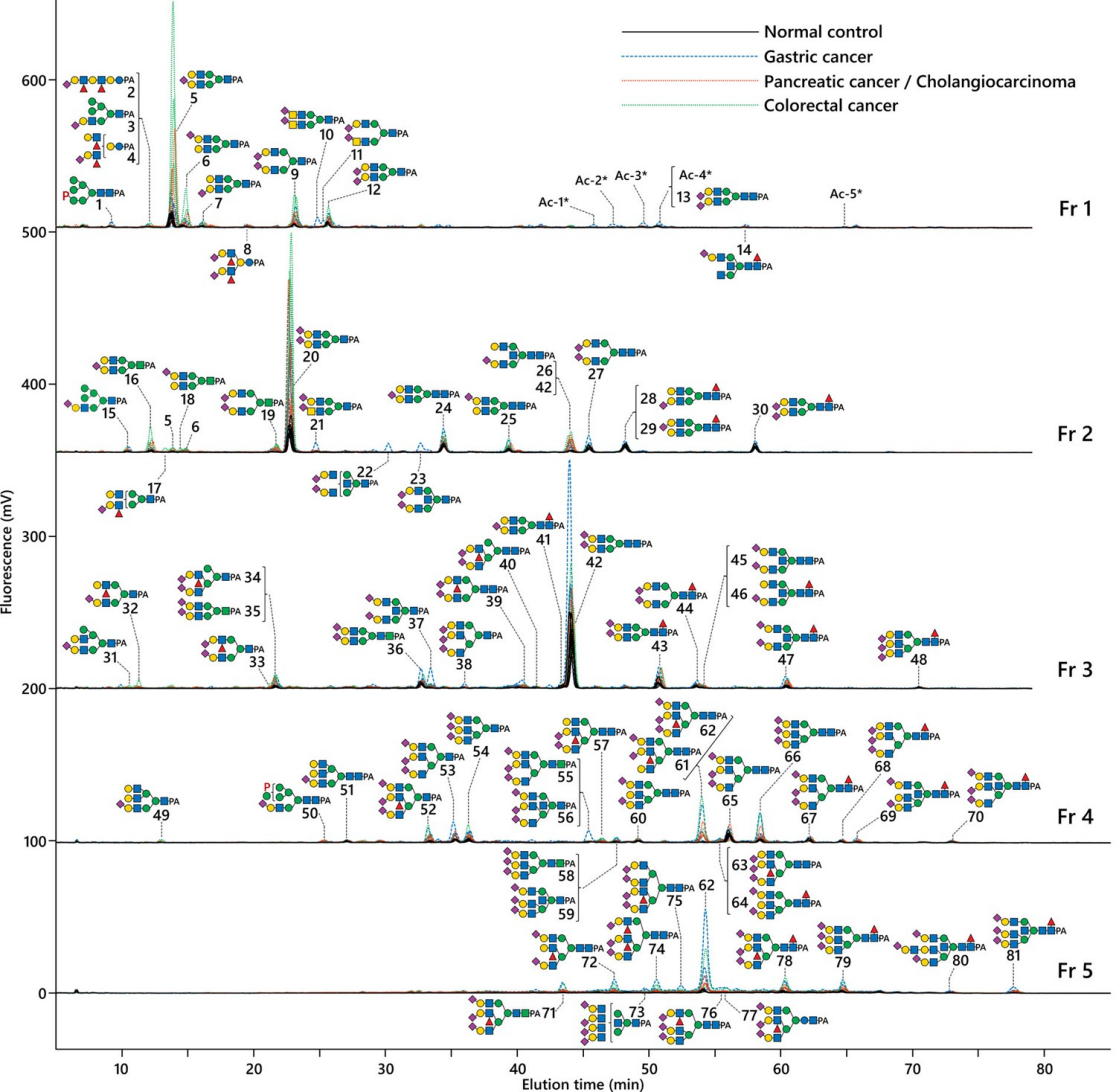
Reversed phase HPLC profiles of the urinary acidic free-glycans a range of bi- to tetra-antennary glycan fractions. PA-labeled free-glycans fractionated by anion-exchange and normal phase HPLC were further separated by reversed phase HPLC on a Shim-pack Scepter C18-120 column. Fluorescent signals were recorded using a RF-10Axl fluorescence detector at ex/em = 315/400 nm (Shimadzu). The amount of urine sample corresponded to 20 μg of creatinine. Representative overlaid chromatograms are shown from five normal controls (N5, N7, N8, N10 and N11), black line; four gastric cancer patients (G2, G8, G10 and G11), blue dotted line; two pancreatic cancer patients (P3 and P5) and two cholangiocarcinoma patients (B2 and B4), orange dotted line; three colorectal cancer patients (C6, C11 and C12), green dotted line. The major glycans comprising the fluorescent peaks are indicated as numbers with symbols of the proposed structures. The glycans are also summarized in Fig 2 in accordance with their basal structure and shown in Table D (S1 File) in numerical order with more detailed information of the structures. Monosaccharide symbols are according to the symbol nomenclature for glycans (SNFG) [52], and indicated as follows: Blue circle, Glc; blue square, GlcNAc; green circle, Man; green square, ManNAc; yellow circle, Gal; yellow square, GalNAc; red triangle, Fuc; purple diamond, Sialic acid (NeuAc); “P”, phosphate.

Urinary free-glycans were extracted and labeled with 2-aminopyridine (PA). The acidic fraction of the glycans was collected by DEAE anion-exchange HPLC (Fig B-a in S2 File). A portion of the acidic fraction was then further separated into five fractions (Fr 1–5) in the range of GU 7–12.5, which included the common bi- to tetra-antennary *N*-glycans, by normal phase (NP-) HPLC with an Amide-HILIC column (Fig B-b in S2 File). These fractions were further separated by reversed phase (RP-) HPLC. The overlaid chromatograms of representative cases are shown in Fig 1.

Structural assignment of glycans was performed by a combination of two-dimensional (2D-) HPLC mapping (NP and RP- HPLC) and mass spectrometry with glycosidase and esterase digestion, periodate treatment and sialic acid linkage-specific alkylamidation. The elution positions of the sample glycans on the HPLCs were converted into values based on those of the standard glycans for 2D-HPLC mapping. For NP-HPLC, the positions were converted into conventional glucose units (NP-GU), based on the degree of polymerization of isomalto-oligosaccharides (IMO). However, IMO-based values in RP-HPLC (RP-GU) showed reduced accuracy for later eluting glycans, such as complex-type *N*-glycans. To circumvent this problem, an alternative correction for the positions on RP-HPLC has been proposed by assigning an *R* value for the reversed phase scale [28,29]. In this study, a modified reversed phase scale was used for correction (Fig A in S2 File). The modified correction procedure was more compatible with the properties of the end-capped C_18_-column packing material and also covered a later elution time range. PA-Gal, which eluted very early in the run, was used as a zero-reference point to minimize deviations arising from the different instruments.

**Fig 2:**
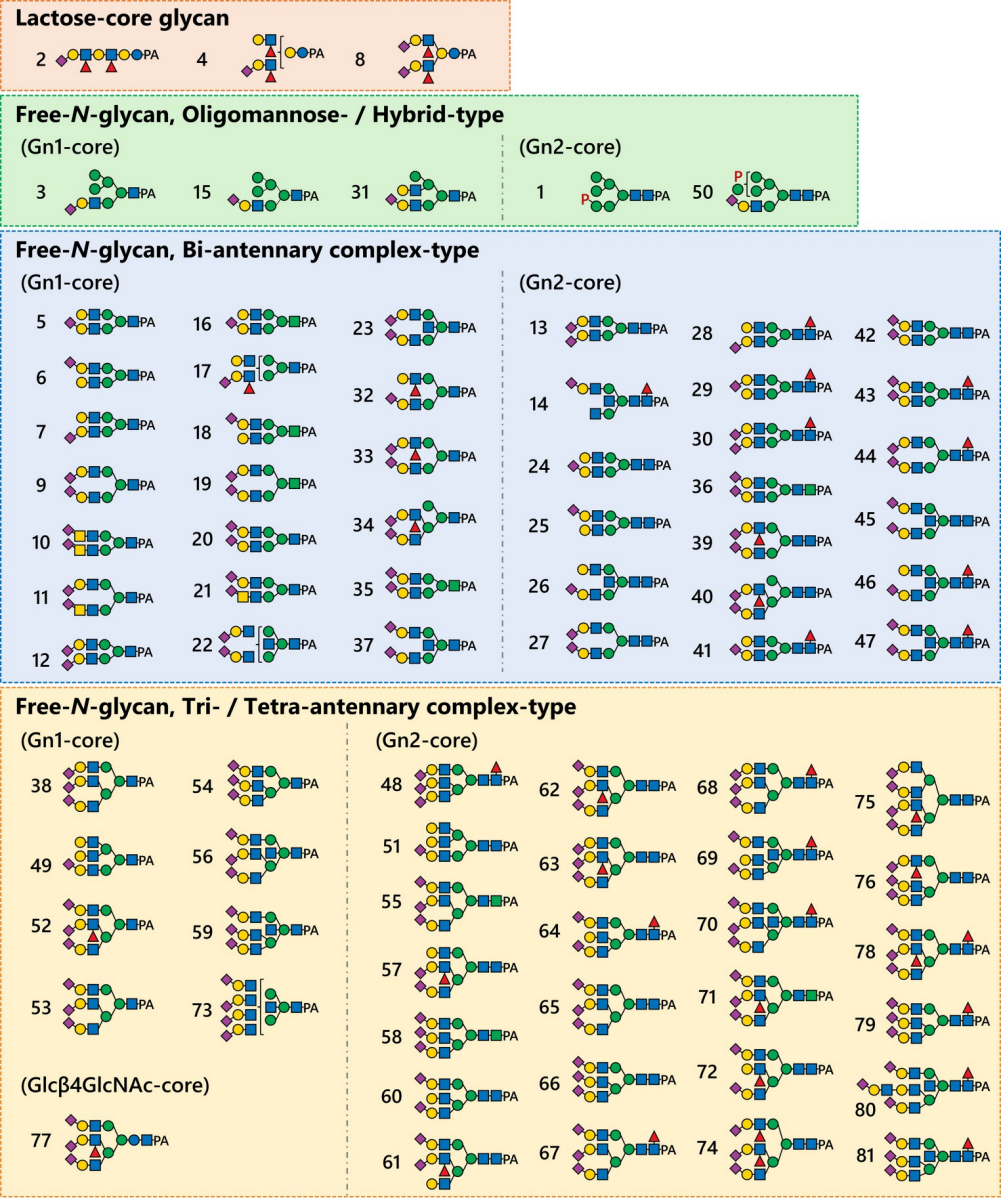
Proposed structures of the urinary acidic free-glycans found in this study. Glycan numbers are from Fig 1. The detailed structural information in numerical order is shown in Table D (S1 File).

In the present study, three lactose-core glycans and 78 of free-*N*-glycans, which share structural features with *N*-glycans found on glycoproteins, were identified. The free-*N*-glycans were classified as oligomannose-, hybrid- or complex-type (bi-, tri- and tetra-antennary) according to their non-reducing terminal structures. The reducing termini of these glycans comprised either GlcNAc_1_ (Gn1-core), GlcNAc_2_ (Gn2-core), or an unusual Glcβ1-4GlcNAc-core. Representative structures of these glycans are described in the following sections. The proposed structures of these glycans are summarized in Fig 2 for each group, and detailed structures in numerical order are shown in Table D (S1 File) along with the converted elution positions and mass values. Clear alterations in the glycan profiles of some cancer patients were observed in RP-HPLC (Fig 1). SRM assays for semi-quantitative high sensitivity comparisons are described in a later section.

### Lactose-core glycans

Glycans with lactose as the reducing terminal structure were minor components in contrast to previous study that specifically analyzed smaller urinary free-glycans [17]. Glycans #2, 4 and 8, which comprise a linear or branched lacto-*N*-neohexaose-type backbone with sialylation and α3-fucosylation, were found at elevated levels in urine samples taken from cancer patients (Fig 1, Fr 1).

### Oligomannose and hybrid type free-*N*-glycans

Minor amounts of acidic oligomannose- and hybrid-type free-*N*-glycans were identified. Hybrid-type glycans #3 and 15 were assigned as sialyl LacNAc-Man_5_GlcNAc_1_ structures (Fig 1, Fr 1, 2). Interestingly, #31 appeared to have an unusual structure containing not only a Man_4_GlcNAc_1_ moiety that characterizes the hybrid-type, but also a bi-antennary structure (Fig 1, Fr 3). This glycan was shifted to the mono-sialylated biantennary glycan #5 by digestion with α-mannosidase, and to Manα1-6Manα1-6(Manα1–3)Manβ1-4GlcNAc-PA by sequential digestion with neuraminidase, β1,4-galactosidase and β-*N*-acetylglucosaminidase (Fig C-a in S2 File). Mass spectrometric analysis after periodate oxidation supported this structural assignment (Fig C-b in S2 File). Glycan #1 was presumed to be a phosphorylated form of an oligo-mannose-type, Man_6_GlcNAc_2_ (Fig 1, Fr 1). The phosphate group was verified by alkaline phosphatase-susceptibility and appeared to be a typical mannose-6-phosphate (Fig D in S2 File). The Gn2-core hybrid type glycan #50 had both sialylation and phosphorylation (Fig 1, Fr 4).

### Bi-antennary free-*N*-glycans

Bi-antennary free-*N*-glycans accounted for a major proportion of urinary acidic free-glycans from both cancer patients and normal controls in the range of analysis used in this study. Simple sialylated structures were abundant in Fr 1–3, such as glycans #5–7, 9, 12 and 20 (Gn1-core) and #24, 25, 27 and 42 (Gn2-core), which have been reported previously [17]. Prominent peaks of Gn1-core glycans having antennal sialyl LacdiNAc (GalNAcβ1-4GlcNAc) or bisecting GlcNAc were identified from one gastric cancer patient G11 (#10, 11, 21–23 and 37; in Fig 1, Fr 1–3). Gn2-core glycans with core-α1,6-fucose and/or bisecting GlcNAc modifications were also relatively abundant (#14, 26, 28–30, 41, 43–47; in Fig 1, Fr 2, 3). Fr 1 contained small amounts of *O*-acetylated glycans (#Ac-1–5), which could be digested by acetylxylanesterase (*Orpinomyces* sp.) to α2,6-sialylated bi-antennary glycans #20 or 42. However, these glycans were not included in the number of glycans identified because they may represent byproducts of side-reactions with acetic acid during the PA-labeling procedure. Glycans with antennal α1,3-fucose were also identified among the peaks that displayed increased intensity in some cancer patients (#17, 32–34, 39 and 40; in Fig 1, Fr 2, 3). Glycans #34 and 40 are bi-antennary with both branches attached to the α1,3-mannose-arm, corresponding to partially degraded forms of tri-/tetra-antennary glycans described in the next section.

### Tri- and tetra-antennary free-*N*-glycans

Tri- and tetra-antennary free-*N*-glycans present in small amounts in Fr 3 were major components in Fr 4, and 5 (Fig 1). Glycosidase digestion analysis of representative tri-antennary glycans are shown in Fig E in S2 File, and the mass values of the glycans after sialic acid linkage-specific alkylamidation (SALSA) are shown in Table E in S1 File. Most of the tri-antennary glycans had 2,4,2’-type branching, having a β1,4-branch on the α1,3-mannose-arm, which resembled the structure of human blood glycoproteins [39,53–55]. These glycans varied in Gn1- or Gn2-core at their reducing termini, the number and linkage positions of sialic acids, and the presence or absence of antennal and/or core fucosylation and bisecting GlcNAc. A trisialyl tri-antennary glycan with a Gn2-core, #65, and its core-fucosylated form, #67, were found at relatively stable levels in both cancer patients and normal controls (Fig 1, Fr 4). In these glycans, the β1,2-branches on both the α1,3- and α1,6-mannose-arms were α2,6-sialylated, while the β1,4-branch on the α1,3-mannose-arm was α2,3-sialylated. Glycans with antennal α1,3-fucosylation, i.e. (+/− sialyl) Lewis X, were detected at increased levels in some cancer patients. The most prominent peak among these was glycan #62 (Fig 1, Fr 4, 5), which contained sialyl Lewis X as a β1,4-branch, corresponding to the α1,3-fucosylated form of #65. A tri-antennary glycan #74 which contained two sialyl Lewis X antennae, and a tri-antennary glycan #78, which was a core-fucosylated form of #62, were also elevated in the urine of some cancer patients. Likewise, the triply α2,6-sialylated glycans #66 and 79 were also detected at increased levels in the urine of some cancer patients (Fig 1, Fr 4, 5). Glycosidase digestion analysis of the Gn2-core, β1,4-branched tri-antennary glycans are shown in Fig E-a in S2 File. Tri-antennary glycans with both bisecting GlcNAc and core-α1,6-fucose were also detected (#69, 70, 80 and 81; Fig 1, Fr 4 and 5). Glycans #69, 70 and 81 share a neutral backbone structure (Fig E-b in S2 File), whereas #80 includes an additional LacNAc on the β1,4-branch (Fig E-c in S2 File). Glycan #75 was a tetra-antennary glycan containing one sialyl Lewis X as the β1,4-branch (Fig 1, Fr 5). Gn1-core glycans, which possessed the corresponding antennal structures of major Gn2-core glycans, were also observed at increased levels in the urine of cancer patients (#62 and 78 → #52, #65 and 67 → #53, #66 and 79 → #54, #68 → 38; Fig 1, Fr 3 and 4; Fig E-d in S2 File). Glycans composed of 2,2’,6’-type branching, another tri-antennary backbone containing a β1,6-branch, were also detected but as minor components (#49 and 51; Fig 1, Fr 5; Fig E-e in S2 File).

### An unusual free-*N*-glycan containing a Glc-GlcNAc-core

**Fig 3:**
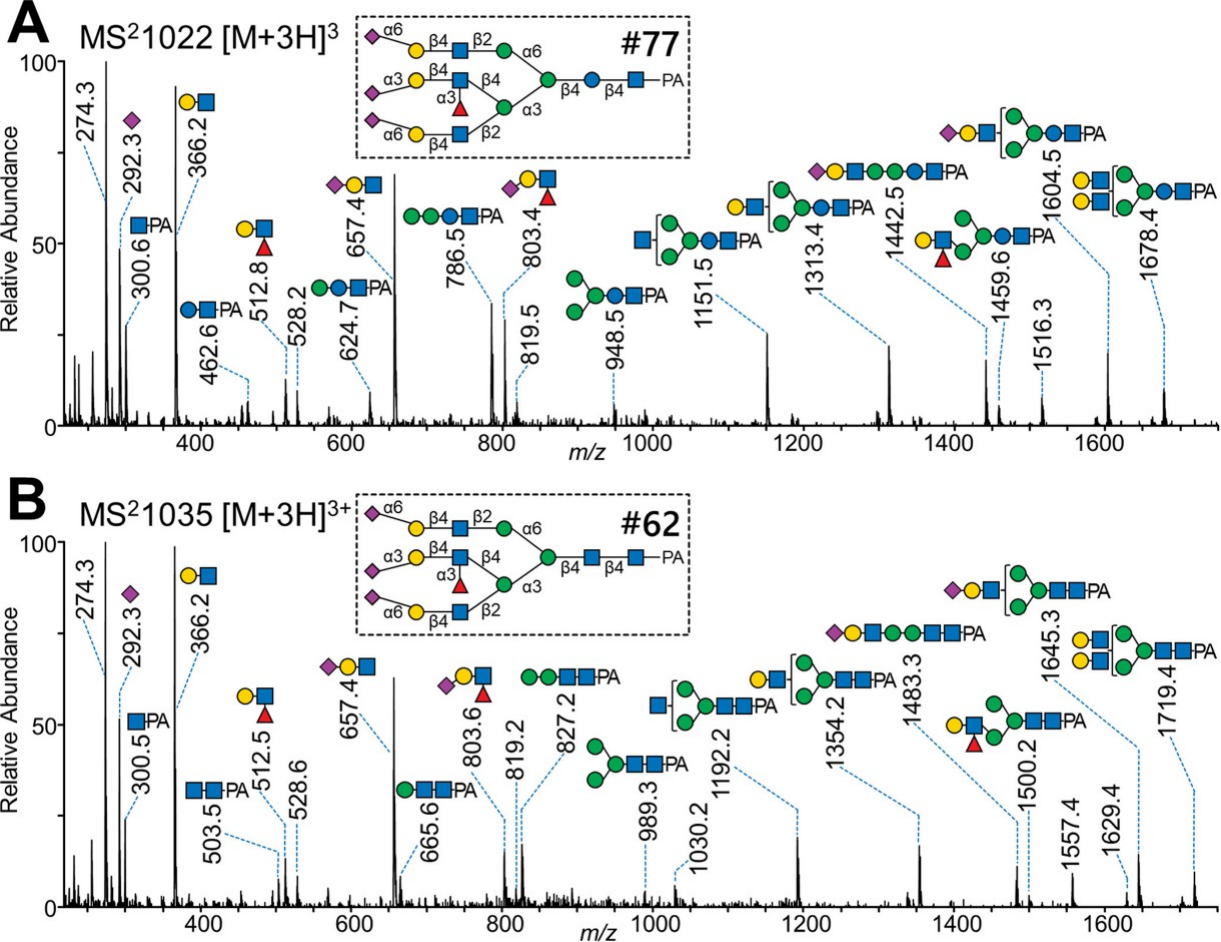
Mass spectrometric comparison of the triantennary free-*N*-glycans containing an unusual core and common GlcNAc_2_-core. MS^2^ (EPI) spectra at 40 eV of CE obtained by the QTRAP 4500 mass spectrometer are shown. (a) Spectrum of glycan #77 at *m/z* 1022 [M+3H]^3+^. (b) Spectrum of glycan #62 at *m/z* 1035 [M+3H]^3+^.

In some cancer patients, a glycan of unusual composition Hex_7_HexNAc_4_dHex_1_NeuAc_3_-PA (#77) was detected as a minor component, which was subjected to detailed structural analysis. The MS^2^ spectrum of this glycan at *m/z* 1022 [M+3H]^3+^ (Fig 3A) showed ions at *m/z* 300, 462, 624, 786 and 948 corresponding to Hex_0–4_HexNAc_1_-PA, which were reminiscent of Gn1-type glycans, although its overall fragmentation pattern was rather similar to that of the tri-antennary Gn2-core glycan #62 at *m/z* 1035 [M+3H]^3+^ (Hex_6_HexNAc_5_dHex_1_NeuAc_3_-PA; Fig 3B). Comparing the spectra of these glycans, the Y-type fragment ions were shifted by 41 Da, except for HexNAc-PA at *m/z* 300, suggesting the presence of an unusual core structure of Hex_4_HexNAc_1_-PA, instead of Man_3_GlcNAc_2_-PA.

**Fig 4:**
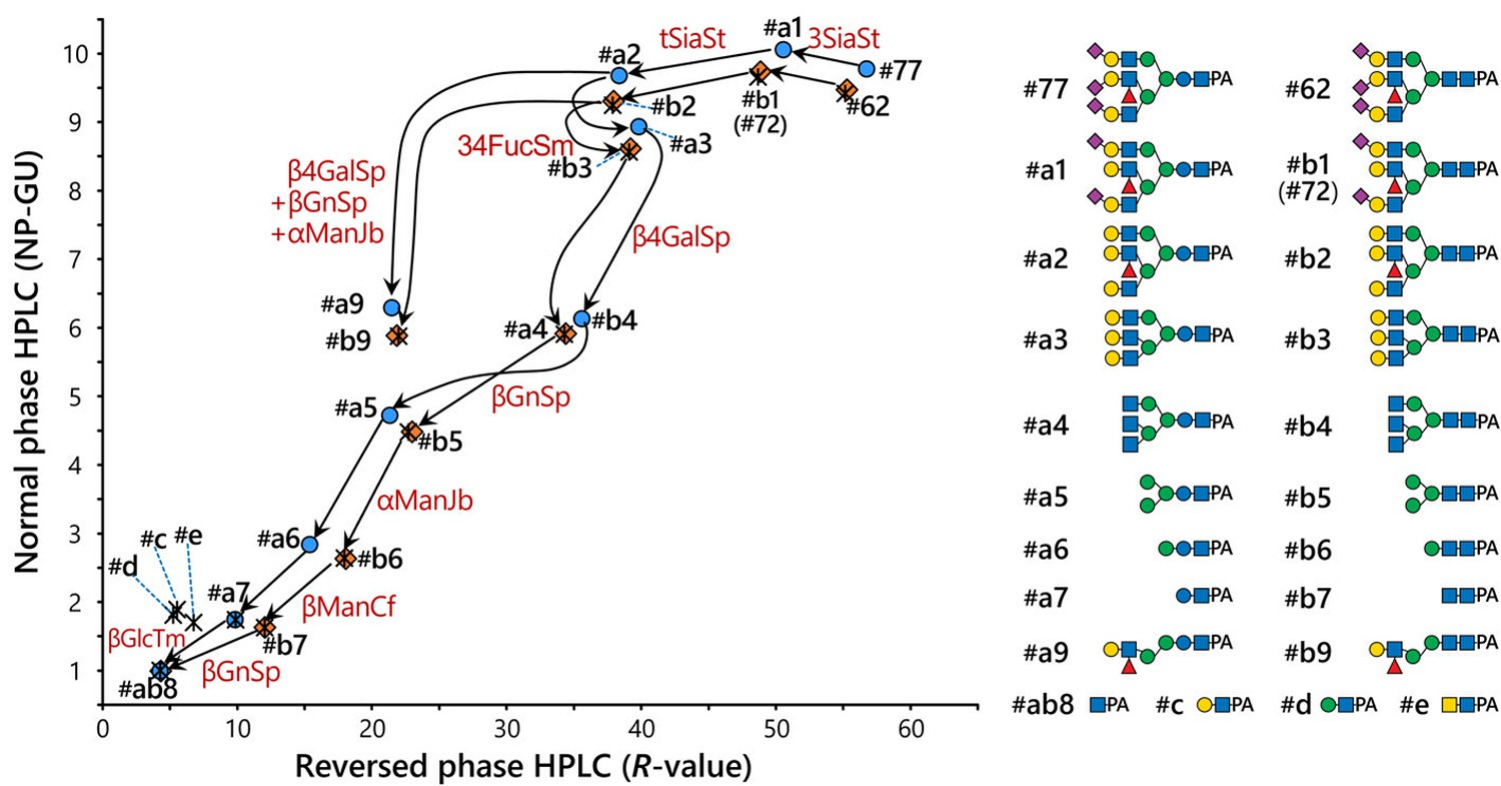
Two-dimensional HPLC mapping analysis of the triantennary free-*N*-glycans containing an unusual core and common GlcNAc_2_-core. The elution positions of PA-glycans on NP- and RP-HPLC were converted into NP-glucose units (NP-GU) and *R* values, respectively. The positions of glycan #77 and its digests (#a1–a7, ab8, a9) are indicated as blue circles, and those of glycan #62 and its digests (#b1–b7, ab8, b9) are indicated as orange diamonds. Asterisks indicate the positions of the standard glycans. Arrows indicate shifts of the elution positions of the glycans by glycosidases. The glycosidases used are indicated as follows: 3SiaSt, α-neuraminidase under the conditions for non-reducing terminal α2,3-linkages from *S*. *typhimurium*; tSiaSt, α-neuraminidase for non-reducing terminal α2,3/6-linkages from *S*. *typhimurium*; 34FucSm, α1,3/4-fucosidase from *Streptomyces* sp. 142; β4GalSp, β1,4-galactosidase from *S*. *pneumoniae*; βGnSp, β-*N*-acetylglucosaminidase from *S*. *pneumoniae*; αManJb, α-mannosidase from jack bean; βManCf, β-mannosidase from *C*. *fimi*; βGlcTm, β-glucosidase from *T*. *maritima*. Glycans #77 and 62 were sequentially digested into the core disaccharides #a7 and b7, corresponding to the positions of standard Glcβ1-4GlcNAc-PA and GlcNAcβ1-4GlcNAc-PA, respectively, but not to #c (Galβ1-4GlcNAc-PA), #d (Manβ1-4GlcNAc-PA) and #e (GalNAcβ1-4GlcNAc-PA).

As shown in the 2D-HPLC map in Fig 4, glycan #77 could be digested sequentially by neuraminidase (from *Salmonella typhimurium*), α1,3/4-fucosidase (from *Streptomyces* sp. 142), β1,4-galactosidase, β-*N*-acetylglucosaminidase (from *Streptococcus pneumoniae*), α-mannosidase (from jack bean) and β-mannosidase (from *Cellulomonas fimi*). The resulting disaccharide (#a7) was consistent with standard Glcβ1-4GlcNAc-PA on the 2D-map, and could be subsequently digested into GlcNAc-PA by β-glucosidase (from *Thermotoga maritima*). Although this β-glucosidase is also known to act on β-linked galactose [56], the disaccharide appeared to contain β-linked glucose based on the discrepancy with Galβ1-4GlcNAc-PA (#c) and Manβ1-4GlcNAc-PA (#d) on the 2D-map. Additionally, the shift pattern of #77 to the core disaccharide was very similar to the sequential digestions of #62 to the Gn2-core, suggesting structural similarity.

**Fig 5:**
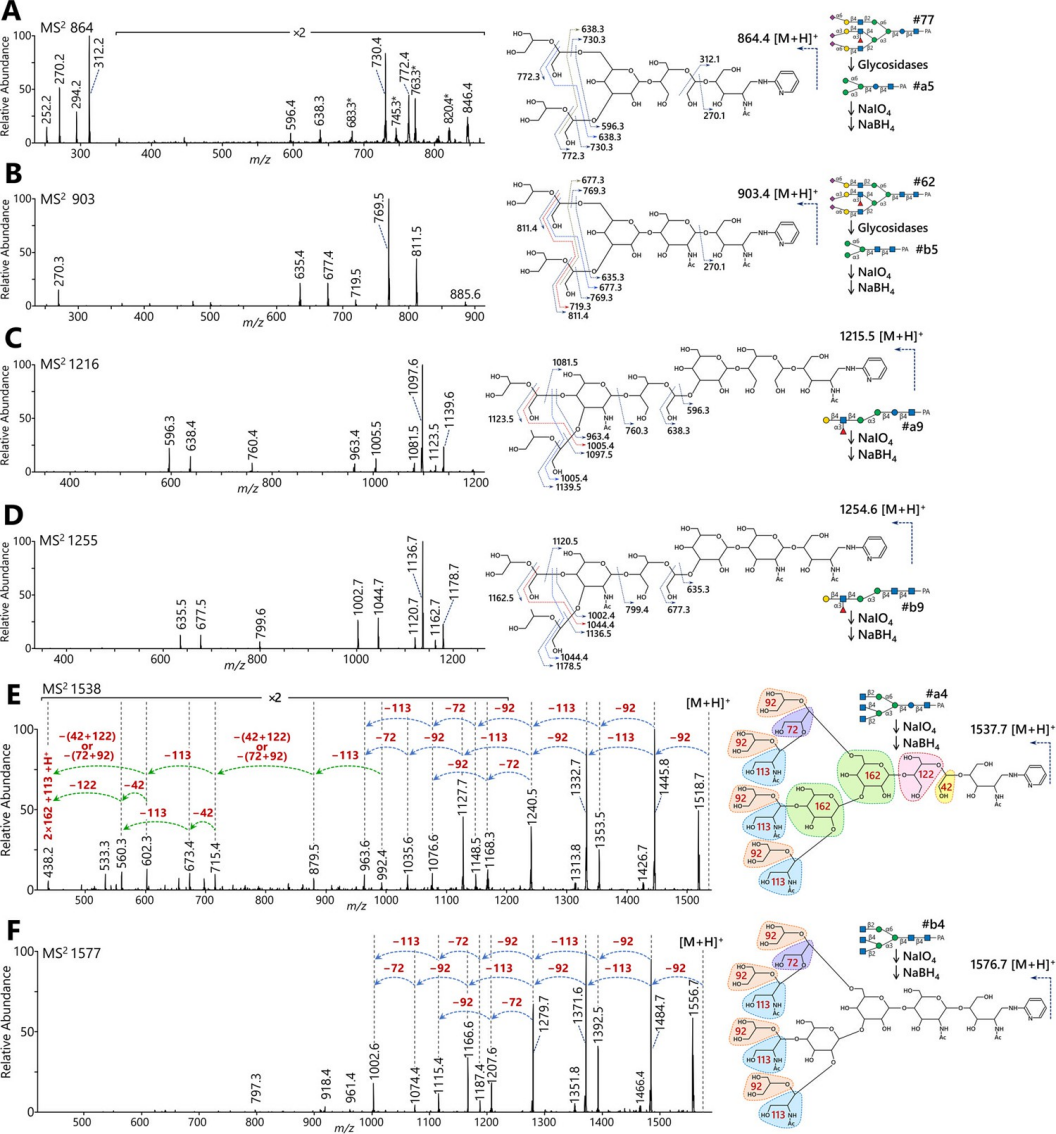
Mass spectrometric comparison of the triantennary free-*N*-glycans after glycosidase digestions and periodate cleavage. To obtain linkage information of core and branching structures, the unusual Glcβ1-4GlcNAc-core glycan #77 and related Gn2-core glycan #62 for comparison were subjected to partial hydrolysis by glycosidases (Fig 4) and periodate cleavage at their C-C bonds between unsubstituted vicinal diols, followed by mass spectrometry. The spectra were obtained using a LTQ XL mass spectrometer. Acetic acid-triethylamine was used as solvent modifier to prevent fragmentation during ionization by forming monovalent precursor ions. Asterisks indicate non-specific signals, which were not thought to be derived from glycan fragments. (a, b) MS^2^ spectra of the trimannosyl pentasaccharides of (a) #a5 from #77 at *m/z* 864 and (b) #b5 from #62 at *m/z* 903. (c, d) MS^2^ spectra of the monoantennary Man_2_-structures with the fucosylated antenna (Lewis X) of (c) #a9 from #77 at *m/z* 1216 and (d) #b9 from #62 at *m/z* 1255. (e, f) MS^2^ spectra of the agalacto-triantennary structures of (e) #a4 from #77 at *m/z* 1538 and (f) #b4 from #62 at *m/z* 1577.

For further linkage analysis of the core structure, Hex_4_HexNAc_1_-PA (#a5) derived from the glycan #77 was subjected to mass spectrometry after periodate oxidation and subsequent reduction (Fig 5A, 5C and 5E). The treated glycan was detected at *m/z* 864 [M+H]^+^ (Hex_4_HexNAc_1_-PA −3×CH_2_O +3×2H). In the MS^2^ spectrum, ions at *m/z* 270 and 312 corresponded to PA*-N*-acetylpentosamine and that with a 42 Da C_2_H_2_O-fragment, respectively. This suggested periodate-cleavage between C-2 and C-3 of the Hex-residue attached to the C-4 OH-group of HexNAc-PA. The sequence of the unusual trimannosyl core, Hex-(Hex-)Hex1-4Hex1-4HexNAc-PA, was confirmed by the presence of an ion at *m/z* 596 (Fig 5A). To confirm the branching structure, periodate treatments and mass spectrometry were performed for the Lewis X-Man_2_-core structure (#a9) at *m/z* 1216 [M+H]^+^ (Hex_4_HexNAc_2_dHex_1_-PA −3×CH_2_O +4×2H; Fig 5C) and the agalacto-tri-antennary structure (#a4) at *m/z* 1538 [M+H]^+^ (Hex_4_HexNAc_4_-PA −CH_2_O +5×2H; Fig 5E). In parallel, MS^2^ experiments of periodate-cleaved structures from the Gn2-core glycan #62 were also performed to confirm structural similarity (Fig 5B, 5D and 5F); namely a common trimannosyl core pentasaccharide (#b5) at *m/z* 903 [M+H]^+^ (Hex_3_HexNAc_2_-PA −3×CH_2_O +2×2H; Fig 5B), Lewis X-Man_2_-core structure (#b9) at *m/z* 1255 [M+H]^+^ (Hex_3_HexNAc_3_dHex_1_-PA −3CH_2_O +3×2H; Fig 5D), and the agalacto-tri-antennary form (#b4) at *m/z* 1577 [M+H]^+^ (Hex_3_HexNAc_5_-PA −CH_2_O +4×2H; Fig 5F). These results suggest that glycan #77 consisted of 2,4,2’-tri-antennary branching with fucosylation on the β1,4-antenna, which was identical to glycan #62, and that only the common Gn2-core was replaced by the unusual Glcβ1-4GlcNAc. We could not identify any other glycans with this unusual core structure among the urinary free-glycans, including the one corresponding to the major bi-antennary structure #42.

### Comparison of the levels of free-glycans by reversed phase HPLC SRM

**Fig 6:**
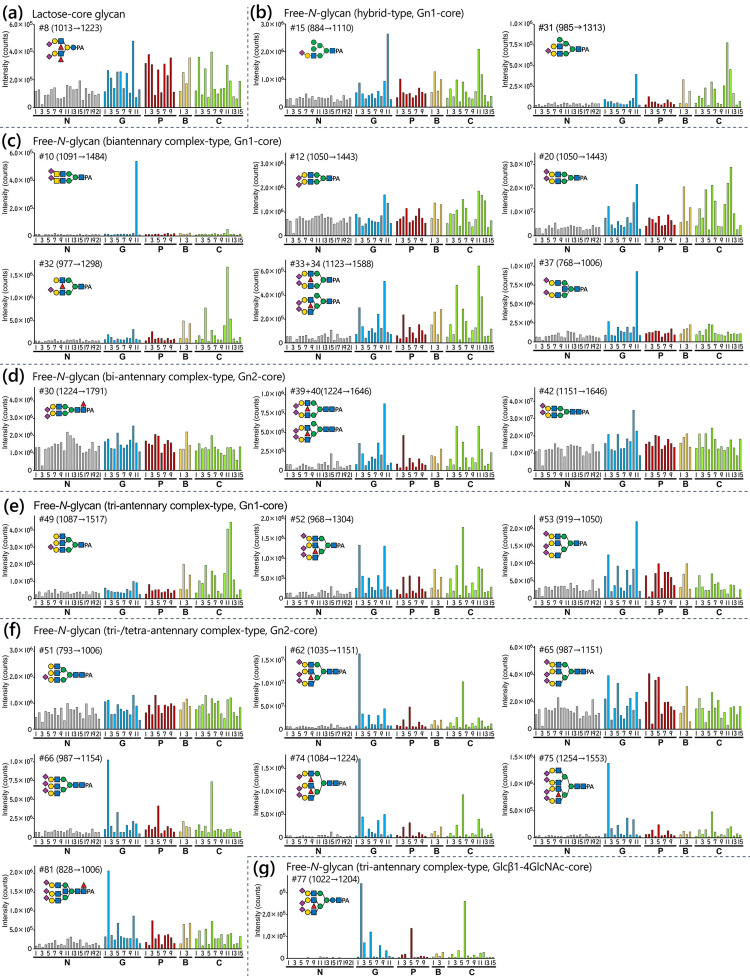
Levels of representative urinary free-glycans from SRM. The peak areas in the extracted ion chromatograms of SRM measurements are shown. The amount of urine sample corresponded to 40 μg of creatinine. The levels of the glycans are indicated by bars as follows: Normal controls (N1–N21), gray; gastric cancer patients (G1–G12), blue; pancreatic cancer patients (P1–P10), red; cholangiocarcinoma patients (B1–B4), yellow; colorectal cancer patients (C1–C15), light green. Glycan number and proposed structure are indicated in each glycan panel. The mass values of Q1 → Q3 (*m/z*) are indicated in parentheses. (a) Lactose-core glycan #8. (b) Hybrid-type, Gn1-core free-*N*-glycans #15 and 31. (c) Biantennary complex-type, Gn1-core free-*N*-glycans #10, 12, 20, 32, 33 + 34 (mixture), and 37. (d) Biantennary complex-type, Gn2-core free-*N*-glycans #30, 39 + 40 (mixture), and 42. (e) Tri-antennary complex-type, Gn1-core free-*N*-glycans #49, 52 and 53. (f) Tri-/tetra-antennary complex-type, Gn2-core free-*N*-glycans #51, 62, 65, 66, 74, 75, and 81. (g) Tri-antennary complex-type, Glcβ1-4GlcNAc-core glycan #77.

To further investigate alterations of glycan levels among cancer patients, selective reaction monitoring was performed. The mass spectrometry-based measurement allows for highly sensitive and specific sample comparison, even in the presence of fused or overlapped peaks in the fluorescent chromatogram of PA-glycans, as long as the separation of structurally similar isomers was achieved. In our previous study, each glycan was independently fractionated by reversed phase HPLC and then analyzed by mass spectrometry, which was time-consuming [17]. Reversed phase HPLC with formic acid-based solvents, commonly used for LC/MS, has been shown to provide a good isomeric separation and on-line MS detection of PA-glycans [57]. Based on this observation, the total acidic fraction from anion-exchange HPLC (Fig B-a in S2 File) was subjected to reversed phase HPLC/ESI-MS/MS, and the specific acidic free-glycans identified in this study were simultaneously measured by SRM for semi-quantitative comparison of samples. A steeper organic solvent gradient setting was applied to improve sensitivity and sample throughput. Moreover, additional acetonitrile was infused into the post-column to improve the efficiency and stability of ionization [45,46]. SRM measurements were performed in scheduled MRM mode. Settings, including Q1 and Q3 mass values and collision energy, are given in Table C in S1 File. Examples of SRM extracted ion chromatograms from a quality control (QC) prepared by mixing PA-glycans of all analyzed samples are shown in Fig F (S2 File). The results of the SRM measurements are shown in Fig 6 and Fig G (S2 File). The *p*-values from the Mann–Whitney *U*-test and the fold change of mean values of the measurements in normal controls and each cancer patient group are shown in Table F. The relationship between glycan structures and cancer patients was attempted to be visualized by performing a principal component analysis (PCA) (Fig H and Supporting Results in S2 File).

Lactose-core glycans, which consist of fucosylated Lacto-*N*-hexaose-type (#8 in Fig 6A; 2 and 4 in Fig G-a in S2 File), showed a small increasing trend in cancer patients. This was similar to the previously reported results for lactose-core glycans with LacNAc extension, sialylation and fucosylation.

Both Gn1- and Gn2-types of hybrid-type free-*N*-glycans showed increased levels in the urine of some cancer patients. A lesser extent elevation was shown by the phosphorylated oligomannose-type #1 (Fig 6B; Fig G-b and G-c in S2 File).

Most bi-antennary Gn1-core free-*N*-glycans showed increased levels in the urine of some cancer patients as reported in our previous study [17]. In particular, α2,6-sialylation and α1,3-fucosylation seemed to contribute to the increase (e.g. #20, 32, 33 + 34), while α2,3-sialylation did not (e.g., #12) as shown in Fig 6D and Fig G-d in S2 File. Glycans with sialyl LacdiNAc or bisecting GlcNAc showed markedly elevated levels only in a gastric cancer patient G11 (e.g., #10 and 37 in Fig 6D). Although some bi-antennary Gn2-core free-*N*-glycans were found at elevated levels in cancer patients the increase was not as marked as for Gn1-core glycans, which was consistent with our previous study (Fig 6D; Fig G-e in S2 File). Indeed, some of these bi-antennary Gn2-core free-*N*-glycans (e.g. #13, 28–30 and 41) displayed relatively stable levels between cancer patients and normal controls. Doubly α2,6-sialylated and α1,6-core fucosylated glycans with/without bisecting GlcNAc (#43 and 47) displayed slightly elevated levels in the urine of cancer patients. As was the case for the Gn1-core glycans, antennal α1,3-fucose appeared to contribute to the elevated levels associated with cancer (refer to #39 + 40 in Fig 6D).

Urinary tri-, tetra-antennary Gn1-core free-*N*-glycans also tended to be found at elevated levels in cancer patients. In particular, α1,3-fucosylated or triply α2,6-sialylated tri-antennary structures seemed to associate with the observed increase (e.g. #52 in Fig 6E; 54 and 59 in Fig G-f in S2 File). Similar to the bi-antennary Gn1-core glycans, structures with bisecting GlcNAc and α2,3-sialylation in addition to α2,6-sialylation showed markedly elevated urinary levels specifically for gastric cancer patients G11 (#56 and 73 in Fig G-f in S2 File). The minor 2,2’,6’-tri-antennary glycan #49 was clearly increased in some patients with cholangiocarcinoma or colorectal cancer (Fig 6E). Tri- and tetra-antennary Gn2-core glycans showed a marked elevation in several cancer patients, in contrast to bi-antennary Gn2-core glycans. As with Gn1-core glycans, α1,3-fucosylation (e.g. #62, 74, 75 and 78) and triple α2,6-sialylation (e.g. #66, 79–81) of Gn2-core glycans appeared to contribute to the increase, while the presence of α2,3-sialylation without α1,3-fucosylation appeared to reduce the extent of increase (e.g. #48, 65 and 68) (Fig 6F; Fig G-g in S2 File). The minor 2,2’,6’-tri-antennary glycan #51, in contrast to the Gn1-core glycan #49, was not detected at obviously elevated levels in cancer patients (Fig 6F). The tri-antennary Glcβ1-4GlcNAc-core glycan #77 showed relatively increased levels in the urine of some cancer patients although the basal level in normal controls was much lower compared to other tri-antennary glycans (Fig 6G).

## Discussion

The patterns of free-glycans in urine appear to reflect alterations in the biosynthesis and degradation pathways of glycans. The increased levels of tri- and tetra-antennary free-*N*-glycans in the urine of cancer patients were particularly marked. Branching of *N*-glycans has been reported to be associated with cancer [58]. The expression of GnT-V, which acts on the β1,6-branch formation on the α1,6-mannose-arm, is upregulated in cancer [59–61]. However, the tri-antennary glycans corresponding to GnT-V products were relatively minor, and only Gn1-type glycan #49 showed an increase in cancer patients, while Gn2-type #51 displayed no marked increase. The tetra-antennary glycans #73 and 75 showed increased levels according to the same trend displayed by other Gn1-type glycans with bisecting GlcNAc (#23 and 37), and tri-antennary glycans with sialyl Lewis X (e.g. #62). Therefore, the contribution of the observed β1,6-branch to the increase seemed to be minor. Most of the tri-antennary glycans found in this study contained β1,4-branching on the α1,3-mannose-arm. The biosynthesis of this branch is initiated by GnT-IV (GnT-IVa and GnT-IVb). However, compared to GnT-V, there are only a few studies that have reported an association with cancer. For example, it has been proposed that GnT-IV is associated with malignancy in colorectal cancer, choriocarcinoma and hepatocarcinoma [62–64]. Moreover, antennal α1,3-fucosylation and α2,6-sialylation are characteristics of free-glycans that show increased levels in the urine of cancer patients. These modifications are also known to be associated with cancer [65–68].

Interestingly, some free-*N*-glycans with tri-antennary structures showed small or no apparent increases in cancer patients. However, the presence of serum Gn2-core sialylated free-*N*-glycans, structurally similar to *N*-glycans on glycoproteins derived from hepatocytes, has been reported in mammals, including human [69,70]. Thus, the excretion of these glycans into urine probably accounts for the basal levels of urinary Gn2-core free-*N*-glycans in healthy individuals.

The observed increase in multi-antennary glycans in cancer patients may not be necessarily derived from cancer cells. Some hepatocyte-derived blood glycoproteins are known to contain tri-/tetra-antennary *N*-glycans containing β1,4-branch with/without α1,3-fucosylation, which share the structural features of free-*N*-glycans found in the present study [39,53,55]. In breast cancer patients, trisialyl tri-antennary glycans with α1,3-fucosylation on the β1,4-branch, which are related to #62 in this study, were found to be elevated in serum. Moreover, the elevation of these glycans correlated with cancer progression along with several acute-phase proteins, α1-acid glycoprotein, α1-antichymotrypsin, and haptoglobin β-chain [54]. Elevated levels of tri-antennary *N*-glycans with sialyl Lewis X and acute phase proteins have been reported in gastric cancer patients, which may be the result of pro-inflammatory cytokine signaling related to carcinogenesis [71]. Several studies have shown that α1,3-fucosylation of glycans of α1- acid glycoprotein in blood is associated with poor prognosis of cancer patients [72,73]. Alterations in the biosynthesis of hepatic glycoproteins, including some acute-phase proteins, may also affect the levels of free-*N*-glycans.

Some of the urinary Gn2-core glycans identified in this study, including those showing cancer-related elevations, contained core-fucose (e.g. #67, 78 and 81). Although the lysosomal degradation of *N*-glycoproteins has been shown to be the major pathway for the generation of complex/hybrid-type free glycans [74,75], the presence of core fucose inhibits the cleavage of *N-*glycosyl asparagine by aspartylglucosaminidase (AGA) in the pathway due to steric hindrance [76]. These findings suggest other pathways that produce free-*N*-glycans are also upregulated in cancer patients, in addition to the lysosomal pathways that are thought to be involved in the production of Gn1-core glycans and some of the Gn2-core glycans. Oligosaccharyltransferase (OST) is a membrane protein complex responsible for the transfer of the glycan from the dolichol-linked *N*-glycan precursor to nascent polypeptides in the endoplasmic reticulum (ER). OST is also reported to generate oligomannose-type free-*N*-glycans [77]. Moreover, it has been hypothesized that the liberated glycans are processed to sialylated free-*N*-glycans with a Gn2-core [69].

In this study, a very small amount of glycan containing a Glcβ1-4GlcNAc-core (#77) instead of a Gn2-core was identified. Interestingly, the relative levels of glycan #77 were very low in normal controls and higher in some cancer patients compared to the structurally similar Gn2-core tri-antennary glycans (e.g. #62). This observation suggested that in addition to antennal modifications, the Glcβ1-4GlcNAc-core might contribute to the specificity in cancer patients. To the best of our knowledge, a core comprising Glcβ1-4GlcNAc has not been reported in *N*-glycans. The glycans possessed a mature non-reducing terminal structure, which was probably synthesized through an *N*-glycan processing pathway, including mannosylation, trimming, branching, galactosylation, sialylation, and fucosylation, similar to typical complex-type *N*-glycans [78]. The biosynthesis of *N*-glycans is initiated from dolichol-linked precursors on the surface of the ER. The formation of the Gn2 structure by transferring the second GlcNAc to the reducing terminal GlcNAc is carried out by the GlcNAc-transferase, ALG13/ALG14 complex. The use of UDP-glucose instead of UDP-GlcNAc in this step might result in the misincorporation of glucose. However, as far as we are aware, no detailed studies on the donor substrate specificity of this enzyme have been reported. Incidentally, examples of glucosylation mediated by GlcNAc-transferases (GnT-III and GnT-V) that are involved in the branch formation of complex-type *N*-glycans have been studied. Intriguingly, these enzymes can exhibit glucosyltransferase activity, albeit with very low efficiency, when UDP-glucose was used as the donor substrate [79,80]. This internal glucosylation may reflect changes to intracellular glucose metabolism in cancer. Indeed, it is known that glucose uptake is frequently increased in cancer cells and inflammatory tissues [81–84]. It has also been reported that UDP-glucose pyrophosphorylase 2 (UGP2), an enzyme responsible for UDP-glucose biosynthesis, is up-regulated in several cancers and that this upregulation is also important for cancer cell survival, proliferation, and metastasis [85–88]. Next, dolichol-linked Gn2 is β1,4-mannosylated by ALG1. To date, no studies using Glcβ1-4GlcNAc as an acceptor substrate for ALG1 have been reported. However, solubilized ALG1 has been shown to further mannosylate the β1,4-mannose upon extended incubation time [89]. This observation suggests that ALG1 has a broad specificity for different acceptors at the C-2 position and that Glcβ1-4GlcNAc could be β1,4-mannosylated. Whether the glycan with a Glcβ1-4GlcNAc-core (#77) core is transferred to the protein and then subsequently liberated remains unclear. It has been shown that OST from yeast can also utilize Glcβ1-4GlcNAc-PP-dolichol with about 60% efficiency as a donor compared with GlcNAcβ1-4GlcNAc-PP-dolichol [90]. Further studies on the Glc1-4GlcNAc-core glycans may lead to the identification of more useful markers and will also expand our understanding of glycan metabolism in cancer.

The unusual bi-antennary hybrid-type #31 followed the similar pattern as other glycans of the Gn1-core hybrid-type and bi-antennary complex-type in terms of elevated levels in the urine of cancer patients. Interestingly, however, the structure of #31 comprises a Man_4_-oligo-mannose moiety as well as a second β1,2-branch on the α1,6-mannose-arm. This β1,2-branch was presumably produced by GnT-II. Although, the substrate specificity for Man_4_-hybrid-type acceptors has not been investigated, GnT-II may tolerate substitutions at the C-6 of the α1,6-mannose-arm to some extent [91,92].

Urine analyzed in the present study was obtained from patients with advanced stages of cancer. Because of the small number of samples it was difficult to evaluate clear differences in the increasing trend of glycans for each type of cancer. Nonetheless, some patients who were negative for established serum tumor markers (i.e., both CA19-9 and CEA), despite their advanced stages of cancer (G5, 8, 10, P5; Table 1), showed increased levels of some free-glycans in urine (e.g. #31, 62, 74 and 77; Fig 6). Therefore, these free-glycans may be potentially useful markers for such patients.

A more detailed study of the alterations of glycans in cancer patients requires the analysis of a larger number of samples. In this study, SRM measurements for comparison of glycan levels was performed with a high sample throughput rate due to shorter measurement times achieved by combining the separation with formic acid-based RP-HPLC. Specifically, the retention of the PA-moiety of PA-glycans on RP-columns is weakened under acidic conditions [93]. The Gn2-core PA-*N*-glycans are known to show relatively strong retention, and favorable separation was achieved using formic acid eluents as described previously [57]. Nonetheless, the present study showed that lactose-core and Gn1-core PA-glycans, which display weak retention, could be well separated as sialylated molecules (Fig F in S2 File). This finding might be due to a decrease in the retention of the PA moiety, which increased the contribution of the carbohydrate moiety of the PA-glycans to facilitate isomeric separation. In the near future we aim to perform simultaneous measurements of various classes of free-glycans in urine to analyze a large number of samples.

## Conclusions

This study focused on analyzing urinary high molecular weight free-glycans as potential markers for cancer. Structural profiling and analysis of their relative levels revealed an increasing trend of mainly α1,3-fucosylated / α2,6-sialylated multi-antennary free-*N*-glycans in cancer patients. Intriguingly, Glcβ1-4GlcNAc, which is a novel structure related to the *N*-glycan core, was identified at elevated levels in some cancer patients. Further studies are needed to elucidate the detailed relationship between the relative levels of these glycans and the corresponding stages or types of cancer.

## Supporting information

S1 File (PDF)

S2 File (PDF)

## References

[1] Kannagi R, Sakuma K, Miyazaki K, Lim KT, Yusa A, Yin J, et al. Altered expression of glycan genes in cancers induced by epigenetic silencing and tumor hypoxia: clues in the ongoing search for new tumor markers. Cancer science. 2010;101(3):586–93. Epub 2010/01/21. doi: 10.1111/j.1349-7006.2009.01455.x . 20085584 PMC11158919

[2] Pinho SS, Reis CA. Glycosylation in cancer: mechanisms and clinical implications. Nature reviews Cancer. 2015;15(9):540–55. Epub 2015/08/21. doi: 10.1038/nrc3982 . 26289314

[3] Thomas D, Rathinavel AK, Radhakrishnan P. Altered glycosylation in cancer: A promising target for biomarkers and therapeutics. Biochimica et biophysica acta Reviews on cancer. 2021;1875(1):188464. Epub 2020/11/07. doi: 10.1016/j.bbcan.2020.188464 ; PubMed Central PMCID: PMC7855613. 33157161 PMC7855613

[4] Kailemia MJ, Xu G, Wong M, Li Q, Goonatilleke E, Leon F, et al. Recent Advances in the Mass Spectrometry Methods for Glycomics and Cancer. Analytical chemistry. 2018;90(1):208–24. Epub 2017/10/20. doi: 10.1021/acs.analchem.7b04202 ; PubMed Central PMCID: PMC6200424. 29049885 PMC6200424

[5] Ruhaak LR, Miyamoto S, Lebrilla CB. Developments in the identification of glycan biomarkers for the detection of cancer. Molecular & cellular proteomics: MCP. 2013;12(4):846–55. Epub 2013/02/01. doi: 10.1074/mcp.R112.026799 ; PubMed Central PMCID: PMC3617331. 23365456 PMC3617331

[6] Ishizuka A, Hashimto Y, Naka R, Kinoshita M, Kakehi K, Seino J, et al. Accumulation of free complex-type N-glycans in MKN7 and MKN45 stomach cancer cells. Biochem J. 2008;413(2):227–37. doi: 10.1042/BJ20071562 . 18399796

[7] Yabu M, Korekane H, Hatano K, Kaneda Y, Nonomura N, Sato C, et al. Occurrence of free deaminoneuraminic acid (KDN)-containing complex-type N-glycans in human prostate cancers. Glycobiology. 2013;23(6):634–42. doi: 10.1093/glycob/cws132 . 22975979

[8] Yabu M, Korekane H, Takahashi H, Ohigashi H, Ishikawa O, Miyamoto Y. Accumulation of free Neu5Ac-containing complex-type N-glycans in human pancreatic cancers. Glycoconj J. 2013;30(3):247–56. doi: 10.1007/s10719-012-9435-9 . 22890903

[9] Sewell AC. Urinary oligosaccharide excretion in disorders of glycolipid, glycoprotein and glycogen metabolism. A review of screening for differential diagnosis. Eur J Pediatr. 1980;134(3):183–94. doi: 10.1007/BF00441471 . 6775948

[10] Humbel R, Collart M. Oligosaccharides in urine of patients with glycoprotein storage diseases. I. Rapid detection by thin-layer chromatography. Clin Chim Acta. 1975;60(2):143–5. doi: 10.1016/0009-8981(75)90119-9 . 1126036

[11] Lemonnier M, Bourrillon R. Characterization and structure of a sialic acid-containing hexasaccharide isolated from human pregnancy urine. Carbohydrate research. 1976;51(1):99–106. Epub 1976/10/01. doi: 10.1016/s0008-6215(00)84039-2 . 1000529

[12] Hallgren P, Lindberg BS, Lundblad A. Quantitation of some urinary oligosaccharides during pregnancy and lactation. J Biol Chem. 1977;252(3):1034–40. Epub 1977/02/10. . 838696

[13] Maury P. Increased excretion of two sialic acid-containing trisaccharides in the urine of patients with rheumatoid arthritis. European journal of clinical investigation. 1978;8(6):405–9. Epub 1978/12/01. doi: 10.1111/j.1365-2362.1978.tb00872.x . 105913

[14] Maury CP, Wegelius O. Urinary sialyloligosaccharide excretion as an indicator of disease activity in rheumatoid arthritis. Rheumatology international. 1981;1(1):7–10. Epub 1981/01/01. doi: 10.1007/BF00541216 . 7346964

[15] Zhang T, Wu X, Ke C, Yin M, Li Z, Fan L, et al. Identification of potential biomarkers for ovarian cancer by urinary metabolomic profiling. J Proteome Res. 2013;12(1):505–12. doi: 10.1021/pr3009572 . 23163809

[16] Shimada I, Shoji M, Futatsuya R, Katoh T, Kominato Y, Sakamoto T, et al. Elevation of ratio of urinary N-acetylneuraminlactose to free sialic acid in some advanced cancer patients. J Gastroenterol. 1995;30(1):21–7. doi: 10.1007/BF01211370 . 7719410

[17] Hanzawa K, Tanaka-Okamoto M, Murakami H, Mukai M, Takahashi H, Omori T, et al. Investigation of acidic free-glycans in urine and their alteration in cancer. Glycobiology. 2021;31(4):391–409. Epub 2020/11/03. doi: 10.1093/glycob/cwaa100 ; PubMed Central PMCID: PMC8091460. 33135073 PMC8091460

[18] Xia B, Asif G, Arthur L, Pervaiz MA, Li X, Liu R, et al. Oligosaccharide Analysis in Urine by MALDI-TOF Mass Spectrometry for the Diagnosis of Lysosomal Storage Diseases. Clinical Chemistry. 2013;59(9):1357–68. doi: 10.1373/clinchem.2012.201053 23676310

[19] Yamashita K, Ohkura T, Okada S, Yabuuchi H, Kobata A. Urinary oligosaccharides of GM1-gangliosidosis. Different excretion patterns of oligosaccharides in the urine of type 1 and type 2 subgroups. J Biol Chem. 1981;256(10):4789–98. Epub 1981/05/25. . 6785275

[20] Ohkura T, Yamashita K, Kobata A. Urinary oligosaccharides of GM1-gangliosidosis. Structures of oligosaccharides excreted in the urine of type 1 but not in the urine of type 2 patients. J Biol Chem. 1981;256(16):8485–90. Epub 1981/08/25. . 6790542

[21] Kim YJ, Varki A. Perspectives on the significance of altered glycosylation of glycoproteins in cancer. Glycoconj J. 1997;14(5):569–76. Epub 1997/08/01. doi: 10.1023/a:1018580324971 . 9298689

[22] Kuraya N, Hase S. Release of O-linked sugar chains from glycoproteins with anhydrous hydrazine and pyridylamination of the sugar chains with improved reaction conditions. J Biochem. 1992;112(1):122–6. Epub 1992/07/01. doi: 10.1093/oxfordjournals.jbchem.a123850 . 1429500

[23] Hase S, Ikenaka T, Matsushima Y. Structure analyses of oligosaccharides by tagging of the reducing end sugars with a fluorescent compound. Biochem Biophys Res Commun. 1978;85(1):257–63. Epub 1978/11/14. doi: 10.1016/s0006-291x(78)80037-0 . 743278

[24] Tanaka-Okamoto M, Yabu M, Mukai M, Takahashi H, Fujiwara Y, Ohue M, et al. Elevation of CA19-9-Related Novel Marker, Core 1 Sialyl Lewis A, in Sera of Adenocarcinoma Patients Verified by a SRM-Based Method. J Proteome Res. 2016;15(1):152–65. doi: 10.1021/acs.jproteome.5b00893 . 26641888

[25] Natsuka S, Hirohata Y, Nakakita S, Sumiyoshi W, Hase S. Structural analysis of N-glycans of the planarian Dugesia japonica. The FEBS journal. 2011;278(3):452–60. Epub 2011/01/06. doi: 10.1111/j.1742-4658.2010.07966.x . 21205195

[26] Tanaka-Okamoto M, Mukai M, Takahashi H, Fujiwara Y, Ohue M, Miyamoto Y. Various sulfated carbohydrate tumor marker candidates identified by focused glycomic analyses. Glycobiology. 2017;27(5):400–15. doi: 10.1093/glycob/cww133 . 28025252

[27] Misonou Y, Shida K, Korekane H, Seki Y, Noura S, Ohue M, et al. Comprehensive clinico-glycomic study of 16 colorectal cancer specimens: elucidation of aberrant glycosylation and its mechanistic causes in colorectal cancer cells. J Proteome Res. 2009;8(6):2990–3005. Epub 2009/03/19. doi: 10.1021/pr900092r . 19292502

[28] Natsuka S, Masuda M, Sumiyoshi W, Nakakita S. Improved method for drawing of a glycan map, and the first page of glycan atlas, which is a compilation of glycan maps for a whole organism. PloS one. 2014;9(7):e102219. Epub 2014/07/10. doi: 10.1371/journal.pone.0102219 ; PubMed Central PMCID: PMC4090225. 25006806 PMC4090225

[29] Yanagida K, Ogawa H, Omichi K, Hase S. Introduction of a new scale into reversed-phase high-performance liquid chromatography of pyridylamino sugar chains for structural assignment. Journal of chromatography A. 1998;800(2):187–98. Epub 1998/04/30. doi: 10.1016/s0021-9673(97)01109-6 . 9561762

[30] Shida K, Misonou Y, Korekane H, Seki Y, Noura S, Ohue M, et al. Unusual accumulation of sulfated glycosphingolipids in colon cancer cells. Glycobiology. 2009;19(9):1018–33. doi: 10.1093/glycob/cwp083 19541771

[31] Minamida S, Aoki K, Natsuka S, Omichi K, Fukase K, Kusumoto S, et al. Detection of UDP-D-Xylose: &alpha;-D-Xyloside &alpha;1&rarr;3Xylosyltransferase Activity in Human Hepatoma Cell Line HepG2. The Journal of Biochemistry. 1996;120(5):1002–6. doi: 10.1093/oxfordjournals.jbchem.a021492 8982869

[32] Omichi K, Hase S. Determination of the linkage positions of reducing-end residues of oligosaccharides by partial periodate oxidation of pyridylaminated derivatives. Anal Biochem. 1995;227(2):404–7. doi: 10.1006/abio.1995.1304 . 7573970

[33] Nishikaze T, Tsumoto H, Sekiya S, Iwamoto S, Miura Y, Tanaka K. Differentiation of Sialyl Linkage Isomers by One-Pot Sialic Acid Derivatization for Mass Spectrometry-Based Glycan Profiling. Analytical chemistry. 2017;89(4):2353–60. Epub 2017/02/15. doi: 10.1021/acs.analchem.6b04150 . 28194959

[34] Abe T, Kameyama A, Natsuka S, Suzuki N. Sequential modifications of glycans by linkage-specific alkylamidation of sialic acids and permethylation. Anal Biochem. 2020;606:113861. Epub 2020/08/03. doi: 10.1016/j.ab.2020.113861 . 32739348

[35] Suzuki N, Abe T, Natsuka S. Quantitative LC-MS and MS/MS analysis of sialylated glycans modified by linkage-specific alkylamidation. Anal Biochem. 2019;567:117–27. Epub 2018/11/24. doi: 10.1016/j.ab.2018.11.014 . 30468716

[36] O’Riordan N, Kane M, Joshi L, Hickey RM. Structural and functional characteristics of bovine milk protein glycosylation. Glycobiology. 2014;24(3):220–36. doi: 10.1093/glycob/cwt162 24398766

[37] Bondt A, Rombouts Y, Selman MH, Hensbergen PJ, Reiding KR, Hazes JM, et al. Immunoglobulin G (IgG) Fab glycosylation analysis using a new mass spectrometric high-throughput profiling method reveals pregnancy-associated changes. Molecular & cellular proteomics: MCP. 2014;13(11):3029–39. Epub 2014/07/10. doi: 10.1074/mcp.M114.039537 ; PubMed Central PMCID: PMC4223489. 25004930 PMC4223489

[38] Yamada E, Tsukamoto Y, Sasaki R, Yagyu K, Takahashi N. Structural changes of immunoglobulin G oligosaccharides with age in healthy human serum. Glycoconj J. 1997;14(3):401–5. Epub 1997/04/01. doi: 10.1023/a:1018582930906 . 9147063

[39] Clerc F, Reiding KR, Jansen BC, Kammeijer GS, Bondt A, Wuhrer M. Human plasma protein N-glycosylation. Glycoconj J. 2016;33(3):309–43. Epub 2015/11/12. doi: 10.1007/s10719-015-9626-2 ; PubMed Central PMCID: PMC4891372. 26555091 PMC4891372

[40] Sumiyoshi W, Nakakita S, Miyanishi N, Yamada K, Hasehira K, Nakakita Y, et al. Hypersialylated type-I lactosamine-containing N-glycans found in Artiodactyla sera are potential xenoantigens. Glycobiology. 2012;22(8):1031–41. Epub 2012/04/12. doi: 10.1093/glycob/cws069 . 22492204

[41] Takegawa Y, Deguchi K, Nakagawa H, Nishimura S. Structural analysis of an N-glycan with "beta1-4 bisecting branch" from human serum IgG by negative-ion MSn spectral matching and exoglycosidase digestion. Analytical chemistry. 2005;77(18):6062–8. Epub 2005/09/15. doi: 10.1021/ac050843e . 16159142

[42] Ramakrishnan B, Shah PS, Qasba PK. alpha-Lactalbumin (LA) stimulates milk beta-1,4-galactosyltransferase I (beta 4Gal-T1) to transfer glucose from UDP-glucose to N-acetylglucosamine. Crystal structure of beta 4Gal-T1 x LA complex with UDP-Glc. J Biol Chem. 2001;276(40):37665–71. Epub 2001/08/04. doi: 10.1074/jbc.M102458200 . 11485999

[43] Do KY, Do SI, Cummings RD. Alpha-lactalbumin induces bovine milk beta 1,4-galactosyltransferase to utilize UDP-GalNAc. J Biol Chem. 1995;270(31):18447–51. Epub 1995/08/04. doi: 10.1074/jbc.270.31.18447 . 7629170

[44] Yuan J, Hashii N, Kawasaki N, Itoh S, Kawanishi T, Hayakawa T. Isotope tag method for quantitative analysis of carbohydrates by liquid chromatography-mass spectrometry. Journal of chromatography A. 2005;1067(1–2):145–52. Epub 2005/04/23. doi: 10.1016/j.chroma.2004.11.070 . 15844519

[45] Feng X, Xiang P, Chen H, Shen M. LC-MS-MS with Post-Column Reagent Addition for the Determination of Zolpidem and its Metabolite Zolpidem Phenyl-4-carboxylic Acid in Oral Fluid after a Single Dose. Journal of analytical toxicology. 2017;41(9):735–43. Epub 2017/10/07. doi: 10.1093/jat/bkx062 . 28985436

[46] Hinneburg H, Chatterjee S, Schirmeister F, Nguyen-Khuong T, Packer NH, Rapp E, et al. Post-Column Make-Up Flow (PCMF) Enhances the Performance of Capillary-Flow PGC-LC-MS/MS-Based Glycomics. Analytical chemistry. 2019;91(7):4559–67. Epub 2019/02/28. doi: 10.1021/acs.analchem.8b05720 . 30810297

[47] Janda I, Weinmann W, Kuehnle T, Lahode M, Alt A. Determination of ethyl glucuronide in human hair by SPE and LC-MS/MS. Forensic science international. 2002;128(1–2):59–65. Epub 2002/09/05. doi: 10.1016/s0379-0738(02)00163-9 . 12208024

[48] Want EJ, Wilson ID, Gika H, Theodoridis G, Plumb RS, Shockcor J, et al. Global metabolic profiling procedures for urine using UPLC-MS. Nature protocols. 2010;5(6):1005–18. Epub 2010/05/08. doi: 10.1038/nprot.2010.50 . 20448546

[49] Want EJ, Masson P, Michopoulos F, Wilson ID, Theodoridis G, Plumb RS, et al. Global metabolic profiling of animal and human tissues via UPLC-MS. Nature protocols. 2013;8(1):17–32. Epub 2012/12/12. doi: 10.1038/nprot.2012.135 . 23222455

[50] Pang Z, Chong J, Zhou G, de Lima Morais DA, Chang L, Barrette M, et al. MetaboAnalyst 5.0: narrowing the gap between raw spectra and functional insights. Nucleic acids research. 2021;49(W1):W388–w96. Epub 2021/05/22. doi: 10.1093/nar/gkab382 ; PubMed Central PMCID: PMC8265181. 34019663 PMC8265181

[51] Xia J, Psychogios N, Young N, Wishart DS. MetaboAnalyst: a web server for metabolomic data analysis and interpretation. Nucleic acids research. 2009;37(Web Server issue):W652–60. Epub 2009/05/12. doi: 10.1093/nar/gkp356 ; PubMed Central PMCID: PMC2703878. 19429898 PMC2703878

[52] Varki A, Cummings RD, Aebi M, Packer NH, Seeberger PH, Esko JD, et al. Symbol Nomenclature for Graphical Representations of Glycans. Glycobiology. 2015;25(12):1323–4. doi: 10.1093/glycob/cwv091 26543186 PMC4643639

[53] Kolarich D, Weber A, Turecek PL, Schwarz HP, Altmann F. Comprehensive glyco-proteomic analysis of human alpha1-antitrypsin and its charge isoforms. Proteomics. 2006;6(11):3369–80. Epub 2006/04/20. doi: 10.1002/pmic.200500751 . 16622833

[54] Abd Hamid UM, Royle L, Saldova R, Radcliffe CM, Harvey DJ, Storr SJ, et al. A strategy to reveal potential glycan markers from serum glycoproteins associated with breast cancer progression. Glycobiology. 2008;18(12):1105–18. doi: 10.1093/glycob/cwn095 18818422

[55] Endo M, Suzuki K, Schmid K, Fournet B, Karamanos Y, Montreuil J, et al. The structures and microheterogeneity of the carbohydrate chains of human plasma ceruloplasmin. A study employing 500-MHz 1H-NMR spectroscopy. J Biol Chem. 1982;257(15):8755–60. Epub 1982/08/10. . 7096333

[56] Gabelsberger J, Liebl W, Schleifer K-H. Purification and properties of recombinant β-glucosidase of the hyperthermophilic bacterium Thermotoga maritima. Applied Microbiology and Biotechnology. 1993;40(1):44–52. doi: 10.1007/BF00170427

[57] Suzuki N, Abe T, Hanzawa K, Natsuka S. Toward robust N-glycomics of various tissue samples that may contain glycans with unknown or unexpected structures. Scientific reports. 2021;11(1):6334. Epub 2021/03/20. doi: 10.1038/s41598-021-84668-x ; PubMed Central PMCID: PMC7973440. 33737529 PMC7973440

[58] Taniguchi N, Korekane H. Branched N-glycans and their implications for cell adhesion, signaling and clinical applications for cancer biomarkers and in therapeutics. BMB reports. 2011;44(12):772–81. Epub 2011/12/23. doi: 10.5483/bmbrep.2011.44.12.772 . 22189679

[59] Dennis JW, Laferté S, Waghorne C, Breitman ML, Kerbel RS. Beta 1–6 branching of Asn-linked oligosaccharides is directly associated with metastasis. Science (New York, NY). 1987;236(4801):582–5. Epub 1987/05/01. doi: 10.1126/science.2953071 . 2953071

[60] Fernandes B, Sagman U, Auger M, Demetrio M, Dennis JW. Beta 1–6 branched oligosaccharides as a marker of tumor progression in human breast and colon neoplasia. Cancer research. 1991;51(2):718–23. Epub 1991/01/15. . 1985789

[61] Seelentag WK, Li WP, Schmitz SF, Metzger U, Aeberhard P, Heitz PU, et al. Prognostic value of beta1,6-branched oligosaccharides in human colorectal carcinoma. Cancer research. 1998;58(23):5559–64. Epub 1998/12/16. . 9850094

[62] D’Arrigo A, Belluco C, Ambrosi A, Digito M, Esposito G, Bertola A, et al. Metastatic transcriptional pattern revealed by gene expression profiling in primary colorectal carcinoma. International journal of cancer. 2005;115(2):256–62. Epub 2005/02/03. doi: 10.1002/ijc.20883 . 15688387

[63] Fan J, Wang S, Yu S, He J, Zheng W, Zhang J. N-acetylglucosaminyltransferase IVa regulates metastatic potential of mouse hepatocarcinoma cells through glycosylation of CD147. Glycoconj J. 2012;29(5–6):323–34. Epub 2012/06/28. doi: 10.1007/s10719-012-9414-1 . 22736280

[64] Niimi K, Yamamoto E, Fujiwara S, Shinjo K, Kotani T, Umezu T, et al. High expression of N-acetylglucosaminyltransferase IVa promotes invasion of choriocarcinoma. British journal of cancer. 2012;107(12):1969–77. Epub 2012/11/22. doi: 10.1038/bjc.2012.496 ; PubMed Central PMCID: PMC3516685. 23169300 PMC3516685

[65] Matsuura N, Narita T, Hiraiwa N, Hiraiwa M, Murai H, Iwase T, et al. Gene expression of fucosyl- and sialyl-transferases which synthesize sialyl Lewisx, the carbohydrate ligands for E-selectin, in human breast cancer. International journal of oncology. 1998;12(5):1157–64. Epub 1998/06/06. doi: 10.3892/ijo.12.5.1157 . 9538143

[66] Barthel SR, Wiese GK, Cho J, Opperman MJ, Hays DL, Siddiqui J, et al. Alpha 1,3 fucosyltransferases are master regulators of prostate cancer cell trafficking. Proceedings of the National Academy of Sciences of the United States of America. 2009;106(46):19491–6. Epub 2009/11/06. doi: 10.1073/pnas.0906074106 ; PubMed Central PMCID: PMC2780742. 19889975 PMC2780742

[67] Schultz MJ, Swindall AF, Bellis SL. Regulation of the metastatic cell phenotype by sialylated glycans. Cancer metastasis reviews. 2012;31(3–4):501–18. Epub 2012/06/16. doi: 10.1007/s10555-012-9359-7 ; PubMed Central PMCID: PMC4079276. 22699311 PMC4079276

[68] Gretschel S, Haensch W, Schlag PM, Kemmner W. Clinical relevance of sialyltransferases ST6GAL-I and ST3GAL-III in gastric cancer. Oncology. 2003;65(2):139–45. Epub 2003/08/22. doi: 10.1159/000072339 . 12931020

[69] Seino J, Fujihira H, Nakakita S-i, Masahara-Negishi Y, Miyoshi E, Hirabayashi J, et al. Occurrence of free sialyl oligosaccharides related to N-glycans (sialyl free N-glycans) in animal sera. Glycobiology. 2016;26(10):1072–85. doi: 10.1093/glycob/cww048 27102284

[70] Iwatsuka K, Watanabe S, Kinoshita M, Kamisue K, Yamada K, Hayakawa T, et al. Free glycans derived from glycoproteins present in human sera. Journal of chromatography B, Analytical technologies in the biomedical and life sciences. 2013;928:16–21. Epub 2013/04/16. doi: 10.1016/j.jchromb.2013.03.010 . 23584042

[71] Bones J, Byrne JC, O’Donoghue N, McManus C, Scaife C, Boissin H, et al. Glycomic and glycoproteomic analysis of serum from patients with stomach cancer reveals potential markers arising from host defense response mechanisms. J Proteome Res. 2011;10(3):1246–65. Epub 2010/12/15. doi: 10.1021/pr101036b . 21142185

[72] Yazawa S, Takahashi R, Yokobori T, Sano R, Mogi A, Saniabadi AR, et al. Fucosylated Glycans in α1-Acid Glycoprotein for Monitoring Treatment Outcomes and Prognosis of Cancer Patients. PloS one. 2016;11(6):e0156277. Epub 2016/06/15. doi: 10.1371/journal.pone.0156277 ; PubMed Central PMCID: PMC4905682. 27295180 PMC4905682

[73] Hashimoto S, Asao T, Takahashi J, Yagihashi Y, Nishimura T, Saniabadi AR, et al. alpha1-acid glycoprotein fucosylation as a marker of carcinoma progression and prognosis. Cancer. 2004;101(12):2825–36. Epub 2004/11/13. doi: 10.1002/cncr.20713 . 15536618

[74] Harada Y, Hirayama H, Suzuki T. Generation and degradation of free asparagine-linked glycans. Cellular and molecular life sciences: CMLS. 2015;72(13):2509–33. Epub 2015/03/17. doi: 10.1007/s00018-015-1881-7 . 25772500 PMC11113800

[75] Seino J, Wang L, Harada Y, Huang C, Ishii K, Mizushima N, et al. Basal autophagy is required for the efficient catabolism of sialyloligosaccharides. J Biol Chem. 2013;288(37):26898–907. Epub 2013/07/25. doi: 10.1074/jbc.M113.464503 ; PubMed Central PMCID: PMC3772239. 23880766 PMC3772239

[76] Noronkoski T, Mononen I. Influence of L-fucose attached alpha 1—>6 to the asparagine-linked N-acetylglucosamine on the hydrolysis of the N-glycosidic linkage by human glycosylasparaginase. Glycobiology. 1997;7(2):217–20. Epub 1997/03/01. doi: 10.1093/glycob/7.2.217 . 9134428

[77] Harada Y, Masahara-Negishi Y, Suzuki T. Cytosolic-free oligosaccharides are predominantly generated by the degradation of dolichol-linked oligosaccharides in mammalian cells. Glycobiology. 2015;25(11):1196–205. Epub 2015/07/25. doi: 10.1093/glycob/cwv055 . 26206502

[78] Schjoldager KT, Narimatsu Y, Joshi HJ, Clausen H. Global view of human protein glycosylation pathways and functions. Nature reviews Molecular cell biology. 2020;21(12):729–49. Epub 2020/10/23. doi: 10.1038/s41580-020-00294-x . 33087899

[79] Ikeda Y, Koyota S, Ihara H, Yamaguchi Y, Korekane H, Tsuda T, et al. Kinetic basis for the donor nucleotide-sugar specificity of beta1, 4-N-acetylglucosaminyltransferase III. J Biochem. 2000;128(4):609–19. Epub 2000/09/30. doi: 10.1093/oxfordjournals.jbchem.a022793 . 11011143

[80] Sasai K, Ikeda Y, Fujii T, Tsuda T, Taniguchi N. UDP-GlcNAc concentration is an important factor in the biosynthesis of beta1,6-branched oligosaccharides: regulation based on the kinetic properties of N-acetylglucosaminyltransferase V. Glycobiology. 2002;12(2):119–27. Epub 2002/03/12. doi: 10.1093/glycob/12.2.119 . 11886845

[81] Love C, Tomas MB, Tronco GG, Palestro CJ. FDG PET of infection and inflammation. Radiographics: a review publication of the Radiological Society of North America, Inc. 2005;25(5):1357–68. Epub 2005/09/15. doi: 10.1148/rg.255045122 . 16160116

[82] Maddalena F, Lettini G, Gallicchio R, Sisinni L, Simeon V, Nardelli A, et al. Evaluation of Glucose Uptake in Normal and Cancer Cell Lines by Positron Emission Tomography. Molecular imaging. 2015;14:490–8. Epub 2015/10/16. . 26461458

[83] Di Chiro G, DeLaPaz RL, Brooks RA, Sokoloff L, Kornblith PL, Smith BH, et al. Glucose utilization of cerebral gliomas measured by [18F] fluorodeoxyglucose and positron emission tomography. Neurology. 1982;32(12):1323–9. Epub 1982/12/01. doi: 10.1212/wnl.32.12.1323 . 6983044

[84] Mochizuki T, Tsukamoto E, Kuge Y, Kanegae K, Zhao S, Hikosaka K, et al. FDG uptake and glucose transporter subtype expressions in experimental tumor and inflammation models. Journal of nuclear medicine: official publication, Society of Nuclear Medicine. 2001;42(10):1551–5. Epub 2001/10/05. . 11585872

[85] Li Y, Zhuang H, Zhang X, Li Y, Liu Y, Yi X, et al. Multiomics Integration Reveals the Landscape of Prometastasis Metabolism in Hepatocellular Carcinoma. Molecular & cellular proteomics: MCP. 2018;17(4):607–18. Epub 2018/01/27. doi: 10.1074/mcp.RA118.000586 ; PubMed Central PMCID: PMC5880115. 29371291 PMC5880115

[86] Zeng C, Xing W, Liu Y. Identification of UGP2 as a progression marker that promotes cell growth and motility in human glioma. Journal of cellular biochemistry. 2019;120(8):12489–99. Epub 2019/03/01. doi: 10.1002/jcb.28515 . 30816613

[87] Wang L, Xiong L, Wu Z, Miao X, Liu Z, Li D, et al. Expression of UGP2 and CFL1 expression levels in benign and malignant pancreatic lesions and their clinicopathological significance. World journal of surgical oncology. 2018;16(1):11. Epub 2018/01/20. doi: 10.1186/s12957-018-1316-7 ; PubMed Central PMCID: PMC5774110. 29347944 PMC5774110

[88] Wolfe AL, Zhou Q, Toska E, Galeas J, Ku AA, Koche RP, et al. UDP-glucose pyrophosphorylase 2, a regulator of glycogen synthesis and glycosylation, is critical for pancreatic cancer growth. Proceedings of the National Academy of Sciences of the United States of America. 2021;118(31). Epub 2021/08/01. doi: 10.1073/pnas.2103592118 ; PubMed Central PMCID: PMC8346792. 34330832 PMC8346792

[89] Xu XX, Li ST, Wang N, Kitajima T, Yoko OT, Fujita M, et al. Structural and functional analysis of Alg1 beta-1,4 mannosyltransferase reveals the physiological importance of its membrane topology. Glycobiology. 2018;28(10):741–53. Epub 2018/06/26. doi: 10.1093/glycob/cwy060 . 29939232

[90] Tai VW, Imperiali B. Substrate specificity of the glycosyl donor for oligosaccharyl transferase. The Journal of organic chemistry. 2001;66(19):6217–28. Epub 2001/09/18. doi: 10.1021/jo0100345 . 11559166

[91] Kadirvelraj R, Yang JY, Sanders JH, Liu L, Ramiah A, Prabhakar PK, et al. Human N-acetylglucosaminyltransferase II substrate recognition uses a modular architecture that includes a convergent exosite. Proceedings of the National Academy of Sciences of the United States of America. 2018;115(18):4637–42. Epub 2018/04/19. doi: 10.1073/pnas.1716988115 ; PubMed Central PMCID: PMC5939069. 29666272 PMC5939069

[92] Reck F, Meinjohanns E, Springer M, Wilkens R, Van Dorst JA, Paulsen H, et al. Synthetic substrate analogues for UDP-GlcNAc: Man alpha 1-6R beta(1–2)-N-acetylglucosaminyltransferase II. Substrate specificity and inhibitors for the enzyme. Glycoconj J. 1994;11(3):210–6. Epub 1994/06/01. doi: 10.1007/BF00731220 . 7841796

[93] Yanagida K, Natsuka S, Hase S. Structural diversity of cytosolic free oligosaccharides in the human hepatoma cell line, HepG2. Glycobiology. 2006;16(4):294–304. Epub 2005/12/31. doi: 10.1093/glycob/cwj074 . 16381657

