# Supplementary material for: Increased levels of acidic free-N-glycans, including multi-antennary and fucosylated structures, in the urine of cancer patients
Source: PLoS One. 2022 Apr 12;17(4):e0266927. doi: 10.1371/journal.pone.0266927 (PMC9004742; doi:10.1371/journal.pone.0266927)
Supplement: S1 File — (PDF) [file pone.0266927.s001.pdf]

Supporting information (S1 File)

Increased levels of acidic free-*N*-glycans, including multi-antennary and fucosylated structures, in the urine of cancer patients

Ken Hanzawa[1], Miki Tanaka-Okamoto[1], Hiroko Murakami[1], Noriko Suzuki[2], Mikio Mukai[3], Hidenori Takahashi[4], Takeshi Omori[4], Kenji Ikezawa[5], Kazuyoshi Ohkawa[5], Masayuki Ohue[4], Shunji Natsuka[2], Yasuhide Miyamoto[1*]

[1] Department of Molecular Biology, Osaka International Cancer Institute, 3-1-69 Otemae, Chuo-ku, Osaka 541-8567, Japan

[2] Graduate School of Science and Technology, Niigata University, 8050 Ikarashi-nino-cho, Nishi-ku, Niigata, 950-2181, Japan

[3] Department of Medical Checkup, Osaka International Cancer Institute, 3-1-69 Otemae, Chuo-ku, Osaka 541-8567, Japan

[4] Department of Gastroenterological Surgery, Osaka International Cancer Institute, 3-1-69 Otemae, Chuo-ku, Osaka 541-8567, Japan

[5] Department of Hepatobiliary and Pancreatic Oncology, Osaka International Cancer Institute, 3-1-69 Otemae, Chuo-ku, Osaka 541-8567, Japan.

[*]Corresponding author
E-mail: miyamoto-ya@mc.pref.osaka.jp (YM)

Table A. HPLC conditions used in this study (p2, 3).

Table B. Standard glycans used for this study (p4–9).

Table C. Settings of SRM measurements of the PA-glycans by scheduled MRM (p10, 11).

Table D. Proposed structures of urinary free-glycans found in this study (p12–15).

Table E. Sialic acid-linkage composition of tri-/tetra-antennary glycans (p16).

Table F. Comparison between cancer patient groups and normal controls for each glycan level (p17–19).

References for Supporting information (p20)

Table A. HPLC conditions used in this study.

(1) Anion-exchange HPLC

Solvent A:	~0.3 mM aqueous ammonia (28% NH3 diluted with water into 1/500,000)
Solvent B:	1 M acetic acid adjusted to pH9.0 with aqueous NH3
Column:	TSKgel DEAE-5PW (10 µm, 7.5 × 75 mm; Tosoh)
Column temp. (°C):	25
Flow rate (mL/min):	0.8
Fluorescence Ex/Em (nm):	310/380

Gradient

Time (min)	% sol. B
0.00	0.0
0.50	0.0
18.00	14.0
30.00	100.0
30.01	0.0
55.00	0.0

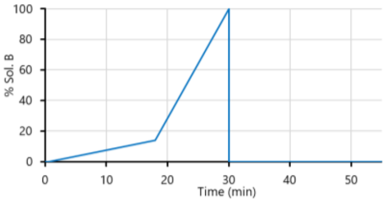

(2) Normal phase HPLC

Solvent A:	5:1 (v/v), Acetonitrile / 0.5M acetic acid+10% (v/v) acetonitrile, adjusted to pH7.3 with triethylamine
Solvent B:	4:5 (v/v), Acetonitrile / 0.5M acetic acid+10% (v/v) acetonitrile, adjusted to pH7.3 with triethylamine
Column:	TSKgel Amide-80 (5 µm, 2 × 250 mm; Tosoh)
Column temp. (°C):	40
Flow rate (mL/min):	0.2
Fluorescence Ex/Em(nm):	310/380

Gradient (2D-mapping)

Time (min)	% sol. B
0.00	0.0
100.00	100.0
100.01	0.0
120.00	0.0

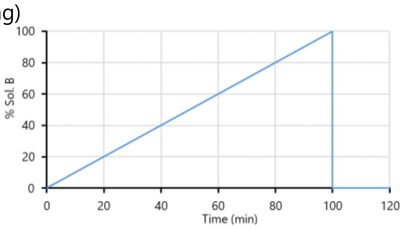

Gradient (fractionation)

Time (min)	% sol. B
0.00	0.0
5.00	20.0
79.00	60.7
90.00	100.0
100.00	100.0
100.01	15.0
120.00	15.0

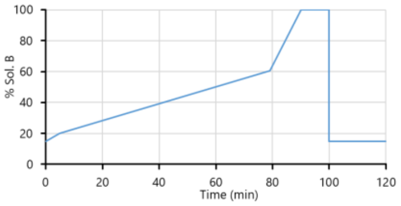

(3) Reversed phase HPLC for fractionation and 2D-mapping

Solvent A:	9:1 (v/v), Water / 0.5M acetic acid, adjusted to pH4.0 with triethylamine
Solvent B:	7:2:1 (v/v), Water / Acetonitrile / 0.5M acetic acid, adjusted to pH4.0 with triethylamine
Column:	Shim-pack Scepter C18-120 (3 µm, 2.1 × 150 mm; Shimadzu)
Column temp. (°C):	35
Flow rate (mL/min):	0.2
Fluorescence Ex/Em (nm)	315/400

Gradient

Time (min)	% sol. B
0.00	0.0
3.00	0.0
84.00	22.5
84.01	100.0
88.00	100.0
88.01	0.0
103.00	0.0

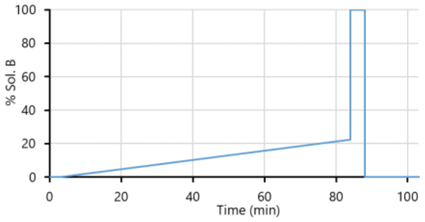

Table A. (continued)

(4) Reversed phase HPLC for routine MS analysis of PA-glycans

Solvent A:	0.4% (v/v) Formic acid / Water
Solvent B:	50% (v/v) Acetonitrile / Water
Column:	InertSustain AQ-C18 (3 µm, 1 × 100 mm; GL Sciences)
Column temp. (°C):	35
Flow rate (mL/min):	0.05

Gradient

Time (min)	% sol. B
0.00	0.0
2.00	0.0
4.00	100.0
5.50	100.0
6.00	0.0
16.00	0.0

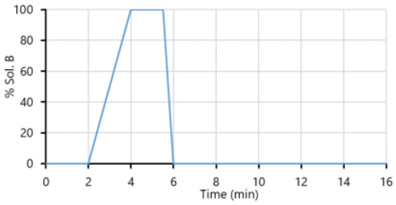

(5) Reversed phase HPLC for MS analysis of PA-glycans for detection as monovalent ions

Solvent A:	1/100 dilution of 0.5 M Acetic acid adjusted to pH6.0 with Triethylamine
Solvent B:	50% (v/v) Acetonitrile / Water
Column:	InertSustain AQ-C18 (3 µm, 1 × 100 mm; GL Sciences)
Column temp. (°C):	35
Flow rate (mL/min):	0.05

Gradient

Time (min)	% sol. B
0.00	0.0
2.00	0.0
4.00	100.0
5.50	100.0
6.00	0.0
16.00	0.0

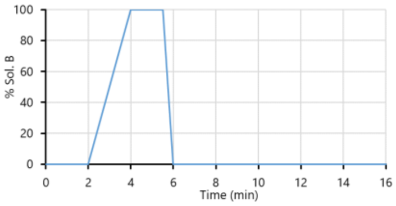

(6) Reversed phase HPLC for SRM experiments of PA-glycans

Solvent A:	0.2% (v/v) Formic acid in Water
Solvent B:	0.2% (v/v) Formic acid in 1:4:5 (v/v) of Acetonitrile / Methanol / Water
Column:	Shim-pack Scepter C18-120 (3 µm, 2.1 × 150 mm; Shimadzu)
Column temp. (°C):	45˚C
Flow rate (mL/min):	0.25
Post-column Addition:	Acetonitrile
Flow rate (mL/min):	0.2

Gradient

Time (min)	% sol. B
0.00	0.0
1.00	2.0
25.50	16.1
26.00	100.0
28.00	100.0
28.50	0.0
40.00	0.0

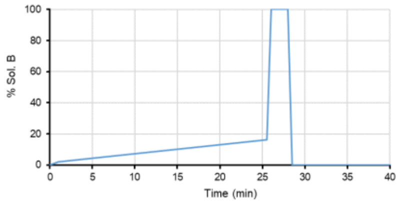

Table B. Standard glycans used for this study.

Standard glycan #	Corresponding glycan # [a]	Structure [b]	NP-GU Std. [c]	RP-GU Std. [d]	R-value Std. [e]	Composition	Notes (source, treatments, etc.)
**Monosaccharide**							
Glc			1.0	1.0	0.3	Hex1-PA	Takara Bio
Man			1.0	...[f]	...[f]	Hex1-PA	Takara Bio
Gal	R0		1.0	0.9	0.0	Hex1-PA	Takara Bio
GlcNAc	R1, ab8		1.0	3.1	4.3	HexNAc1-PA	Takara Bio
ManNAc			0.9	2.5	2.8	HexNAc1-PA	Takara Bio
GalNAc			0.9	3.0	3.9	HexNAc1-PA	Takara Bio
TalNAc			1.0	3.0	3.9	HexNAc1-PA	Sigma-Aldrich
**Di-saccharide**							
Lac-1		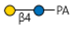	2.0	1.9	1.2	Hex2-PA	Takara Bio
Ln	c	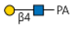	1.8	3.4	5.3	Hex1HexNAc1-PA	Sigma-Aldrich
Gn1-1	d	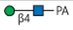	1.9	3.4	5.5	Hex1HexNAc1-PA	Urinary free-glycan, glycosidase
GGn	a7	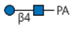	1.7	4.3	9.9	Hex1HexNAc1-PA	Enzymatic synthesis
Ldn	e	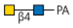	1.7	3.8	6.8	HexNAc2-PA	Enzymatic synthesis
GnF	R2	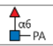	1.4	4.9	13.0	HexNAc1dHex1-PA	Tokyo Chemical Industry, glycosidase
Gn2-1	b7	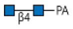	1.6	4.7	12.1	HexNAc2-PA	Tokyo Chemical Industry, glycosidase
**Gn2-core Neutral**							
Gn2-2		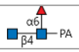	2.2	6.9	22.9	HexNAc2dHex1-PA	Tokyo Chemical Industry
Gn2-3	b6, sD-3	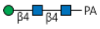	2.6	5.8	17.9	Hex1HexNAc2-PA	Bovine milk lactoferrin, hydrazinolysis, glycosidase
Gn2-4		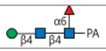	3.1	8.5	28.3	Hex1HexNAc2dHex1-PA	Human blood γ-globulin, glycosidase
Gn2-5		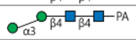	3.6	5.7	17.3	Hex2HexNAc2-PA	Takara Bio
Gn2-6		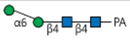	3.5	6.9	23.1	Hex2HexNAc2-PA	Human blood γ-globulin, hydrazinolysis, glycosidase
Gn2-7		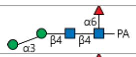	4.1	8.3	28.0	Hex2HexNAc2dHex1-PA	Human blood γ-globulin, hydrazinolysis, glycosidase
Gn2-8		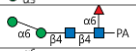	3.9	9.5	31.8	Hex2HexNAc2dHex1-PA	Human blood γ-globulin, hydrazinolysis, glycosidase
Gn2-9	b5	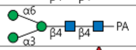	4.5	6.8	22.7	Hex3HexNAc2-PA	Takara Bio
Gn2-10		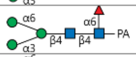	4.9	9.5	31.4	Hex3HexNAc2dHex1-PA	Seikagaku Corp.
Gn2-11		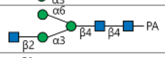	5.0	6.7	22.3	Hex3HexNAc3-PA	Glyence
Gn2-12		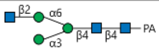	5.0	9.2	30.5	Hex3HexNAc3-PA	Seikagaku Corp.
Gn2-13		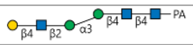	5.4	9.5	31.7	Hex3HexNAc3-PA	Human blood γ-globulin, hydrazinolysis, glycosidase
Gn2-14		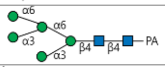		6.8	22.9	Hex5HexNAc2-PA	Takara Bio
Gn2-15		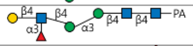	5.9	6.6	22.0	Hex3HexNAc3dHex1-PA	Human plasma-α1-AGP, hydrazinolysis, glycosidase
Gn2-16		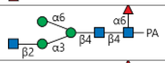	5.5	9.4	31.0	Hex3HexNAc3dHex1-PA	Seikagaku Corp.
Gn2-17		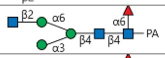	6.4	13.1	38.9	Hex3HexNAc3dHex1-PA	Human blood γ-globulin, hydrazinolysis, glycosidase
Gn2-18		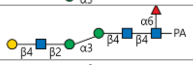	5.6	13.3	39.7	Hex3HexNAc3dHex1-PA	Human blood γ-globulin, hydrazinolysis, glycosidase
Gn2-19	sE-19	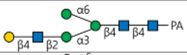	6.0	7.3	24.5	Hex4HexNAc3-PA	Human blood γ-globulin, hydrazinolysis, glycosidase
Gn2-20	sE-18	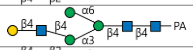	6.0	8.1	27.5	Hex4HexNAc3-PA	Human plasma-α1-AGP, hydrazinolysis, glycosidase
Gn2-21	sE-20	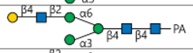	5.9	9.9	32.3	Hex4HexNAc3-PA	Human blood γ-globulin, hydrazinolysis, glycosidase
Gn2-22		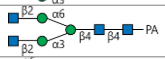	5.5	8.4	28.4	Hex3HexNAc4-PA	Takara Bio
Gn2-23	sD-1	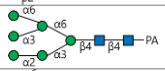	7.1	6.1	19.1	Hex6HexNAc2-PA	Takara Bio
Gn2-24		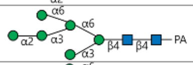	6.9	7.6	25.6	Hex6HexNAc2-PA	Takara Bio
Gn2-25		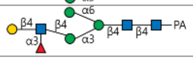	6.8	7.9	26.6	Hex4HexNAc3dHex1-PA	Human plasma-α1-AGP, hydrazinolysis, glycosidase

Table B. (continued)

Standard glycan #	Correspond-ing glycan # [a]	Structure [b]	NP-GU Std. [c]	RP-GU Std. [d]	R-value Std. [e]	Composition	Notes (source, treatments, etc.)
Gn2-26		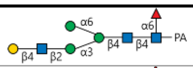	5.4	10.2	33.1	Hex4HexNAc3dHex1-PA	Human blood γ-globulin, hydrazinolysis, glycosidase
Gn2-27		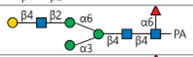	6.3	14.2	40.5	Hex4HexNAc3dHex1-PA	Human blood γ-globulin, hydrazinolysis, glycosidase
Gn2-28		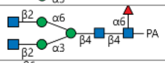	5.7	13.3	39.1	Hex3HexNAc4dHex1-PA	Human blood γ-globulin, hydrazinolysis
Gn2-29	R3	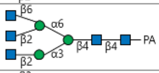	6.1	6.6	21.8	Hex3HexNAc5-PA	Mouse serum, hydrazinolysis, glycosidase
Gn2-30	b4	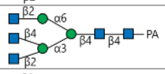	5.9	10.6	34.3	Hex3HexNAc5-PA	Takara Bio
Gn2-31		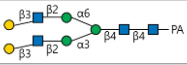	7.0	8.4	27.9	Hex5HexNAc4-PA	Bovine serum, hydrazinolysis, glycosidase
Gn2-32		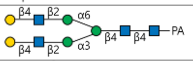	7.3	9.7	32.1	Hex5HexNAc4-PA	Takara Bio
Gn2-33	R4	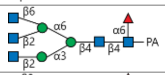	6.5	9.2	30.5	Hex3HexNAc5dHex1-PA	Mouse serum, hydrazinolysis, glycosidase
Gn2-34		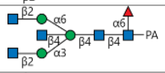	6.1	>20.0	48.6	Hex3HexNAc5dHex1-PA	Human blood γ-globulin, hydrazinolysis, glycosidase
Gn2-35	sE-14	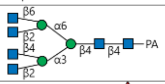	6.6	8.4	28.2	Hex3HexNAc6-PA	Takara Bio
Gn2-36		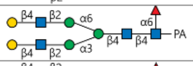	7.6	13.7	40.2	Hex5HexNAc4dHex1-PA	Takara Bio
Gn2-37		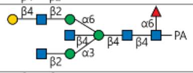	6.8	>20.0	50.7	Hex4HexNAc5dHex1-PA	Seikagaku Corp.
Gn2-38	R5	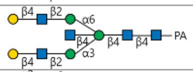	7.3	16.2	43.0	Hex5HexNAc5-PA	Human blood γ-globulin, hydrazinolysis, glycosidase
Gn2-39		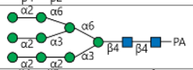	9.6	5.4	15.7	Hex9HexNAc2-PA	Takara Bio
Gn2-40	R6	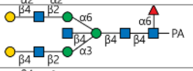	7.7	>20.0	51.9	Hex5HexNAc5dHex1-PA	Seikagaku Corp.
Gn2-41	sE-24	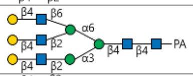	8.7	7.4	24.7	Hex6HexNAc5-PA	Mouse serum, hydrazinolysis, glycosidase
Gn2-42	b3, sE-1	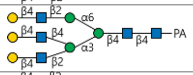	8.6	13.1	38.9	Hex6HexNAc5-PA	Takara Bio
Gn2-43		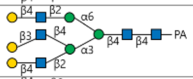	8.4	13.8	40.0	Hex6HexNAc5-PA	Takara Bio
Gn2-44		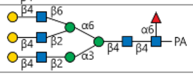	9.1	10.0	32.6	Hex6HexNAc5dHex1-PA	Mouse serum, hydrazinolysis, glycosidase
Gn2-45	b2, sE-3	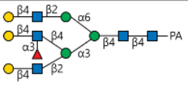	9.3	12.5	37.8	Hex6HexNAc5dHex1-PA	Takara Bio
Gn2-46	sE-2	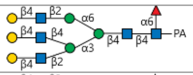	8.9	19.5	46.5	Hex6HexNAc5dHex1-PA	Takara Bio
Gn2-47		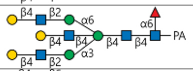	8.5	>20.0	54.3	Hex6HexNAc5dHex1-PA	Human blood γ-globulin, hydrazinolysis, glycosidase
Gn2-48		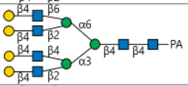	10.0	9.7	31.8	Hex7HexNAc6-PA	Takara Bio
Gn2-49		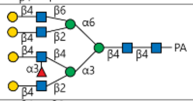	10.7	9.1	30.3	Hex7HexNAc6dHex1-PA	Takara Bio
Gn2-50		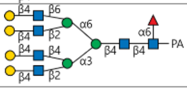	10.3	13.3	39.2	Hex7HexNAc6dHex1-PA	Takara Bio

Table B. (continued)

Standard glycan #	Corresponding glycan # [a]	Structure [b]	NP-GU Std. [c]	RP-GU Std. [d]	R-value Std. [e]	Composition	Notes (source, treatments, etc.)
**Gn2-core Acidic**							
Gn2-51		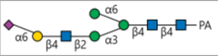	6.4	9.3	30.9	Hex4HexNAc3NeuAc1-PA	Human blood γ-globulin, hydrazinolysis, glycosidase
Gn2-52		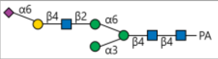	6.3	16.1	43.4	Hex4HexNAc3NeuAc1-PA	Takara Bio, glycosidase
Gn2-53		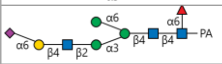	6.8	13.6	39.9	Hex4HexNAc3dHex1NeuAc1-PA	Human blood γ-globulin, hydrazinolysis, glycosidase
Gn2-54		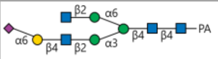	6.8	11.2	35.7	Hex4HexNAc4NeuAc1-PA	Human blood γ-globulin, hydrazinolysis, glycosidase
Gn2-55		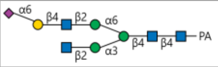	6.7	13.7	40.2	Hex4HexNAc4NeuAc1-PA	Takara Bio, glycosidase
Gn2-56		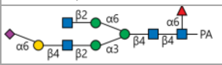	7.2	17.0	44.3	Hex4HexNAc4dHex1NeuAc1-PA	Human blood γ-globulin, hydrazinolysis, glycosidase
Gn2-57	24	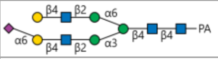	7.7	12.1	37.5	Hex5HexNAc4NeuAc1-PA	Takara Bio
Gn2-58	25	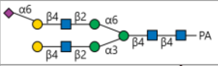	7.6	15.2	41.8	Hex5HexNAc4NeuAc1-PA	Takara Bio
Gn2-59	41	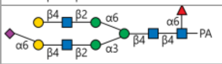	7.9	18.4	45.4	Hex5HexNAc4dHex1NeuAc1-PA	Human blood γ-globulin, hydrazinolysis
Gn2-60	26	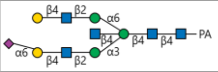	7.7	19.0	46.0	Hex5HexNAc5NeuAc1-PA	Human blood γ-globulin, hydrazinolysis, glycosidase
Gn2-61	46	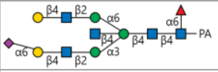	8.1	>20.0	55.0	Hex5HexNAc5dHex1NeuAc1-PA	Human blood γ-globulin, hydrazinolysis
Gn2-62	51	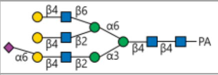	9.0	9.2	30.9	Hex6HexNAc5NeuAc1-PA	Mouse serum, hydrazinolysis, glycosidase
Gn2-63		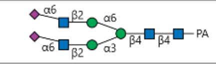	5.9	>20.0	47.1	Hex3HexNAc4NeuAc2-PA	Bovine serum, hydrazinolysis, glycosidase
Gn2-64		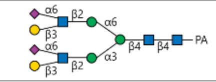	7.3	15.9	42.9	Hex5HexNAc4NeuAc2-PA	Bovine serum, hydrazinolysis, glycosidase
Gn2-65	42	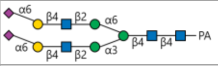	7.9	18.9	46.0	Hex5HexNAc4NeuAc2-PA	Takara Bio
Gn2-66	43	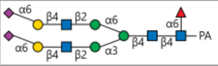	8.2	>20.0	52.0	Hex5HexNAc4dHex1NeuAc2-PA	Human blood γ-globulin, hydrazinolysis
Gn2-67		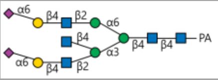	8.3	>20.0	49.7	Hex5HexNAc5NeuAc2-PA	Human plasma-α1-AGP, hydrazinolysis, glycosidase
Gn2-68	45	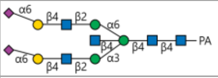	8.0	>20.0	54.9	Hex5HexNAc5NeuAc2-PA	Human blood γ-globulin, hydrazinolysis, glycosidase
Gn2-69	47	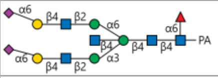	8.3	>20.0	61.2	Hex5HexNAc5dHex1NeuAc2-PA	Human blood γ-globulin, hydrazinolysis
Gn2-70	60	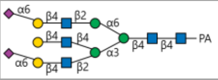	9.0	>20.0	50.5	Hex6HexNAc5NeuAc2-PA	Human plasma-α1-AGP, hydrazinolysis, glycosidase
Gn2-71	72, b1	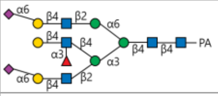	9.7	>20.0	48.7	Hex6HexNAc5dHex1NeuAc2-PA	Human plasma-α1-AGP, hydrazinolysis, glycosidase
Gn2-72		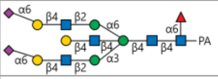	9.1	>20.0	62.3	Hex6HexNAc5dHex1NeuAc2-PA	Human blood γ-globulin, hydrazinolysis
Gn2-73		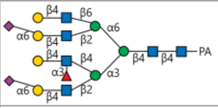	10.9	14.6	42.4	Hex7HexNAc6dHex1NeuAc2-PA	Human plasma-α1-AGP, hydrazinolysis, glycosidase
Gn2-74		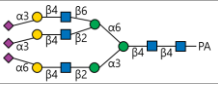	8.7	>20.0	48.1	Hex6HexNAc5NeuAc3-PA	Mouse serum, hydrazinolysis, glycosidase

Table B. (continued)

Standard glycan #	Correspond-ing glycan # [a]	Structure [b]	NP-GU Std. [c]	RP-GU Std. [d]	R-value Std. [e]	Composition	Notes (source, treatments, etc.)
Gn2-75	65	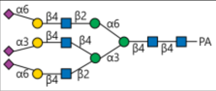	8.9	>20.0	57.0	Hex6HexNAc5NeuAc3-PA	Human plasma-α1-AGP, hydrazinolysis
Gn2-76	66	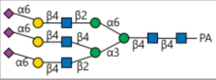	9.3	>20.0	59.3	Hex6HexNAc5NeuAc3-PA	Takara Bio
Gn2-77	62	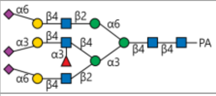	9.4	>20.0	55.1	Hex6HexNAc5dHex1NeuAc3-PA	Human plasma-α1-AGP, hydrazinolysis
Gn2-78		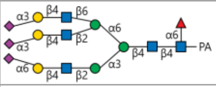	9.0	>20.0	55.9	Hex6HexNAc5dHex1NeuAc3-PA	Mouse serum, hydrazinolysis, glycosidase
Gn2-79		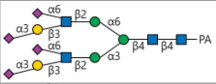	7.2	>20.0	53.8	Hex5HexNAc4NeuAc4-PA	Bovine serum, hydrazinolysis
Gn2-80	75	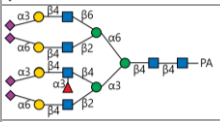	10.4	>20.0	53.8	Hex7HexNAc6dHex1NeuAc4-PA	Human plasma-α1-AGP, hydrazinolysis
**Gn2-core modified**							
EA-Gn2-60	R7	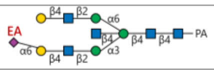	…	>20.0	61.1	Hex5HexNAc5-(ethylamide-)NeuAc1-PA	Human blood γ-globulin, hydrazinolysis, glycosidase, ethylamidation
EA-Gn2-61	R8	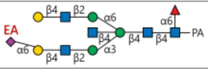	…	>20.0	70.0	Hex5HexNAc5dHex1-(ethylamide-)NeuAc1-PA	Human blood γ-globulin, hydrazinolysis, ethylamidation
D4-std	D4-std	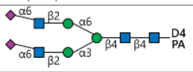	…	…	…	Hex3HexNAc4NeuAc2-D4PA	Bovine serum, hydrazinolysis, D_4-PA, glycosidase
**Gn1-core Neutral**							
Gn1-2		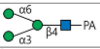	4.0	2.5	2.7	Hex3HexNAc1-PA	Sialylglycopeptide, glycosidase
Gn1-3	sC-2	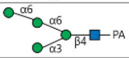	5.0	2.5	2.3	Hex4HexNAc1-PA	Urinary free-glycan [s1]
Gn1-4	sC-3	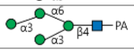	4.8	3.0	3.4	Hex4HexNAc1-PA	Bovine milk lactoferrin, glycosidase
Gn1-5	b9	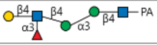	5.2	5.1	14.0	Hex3HexNAc2dHex1-PA	Human plasma-α1-AGP, glycosidase
Gn1-6		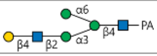	5.5	3.0	3.8	Hex4HexNAc2-PA	Sialylglycopeptide, glycosidase
Gn1-7		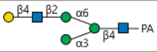	5.6	3.6	6.1	Hex4HexNAc2-PA	Sialylglycopeptide, glycosidase
Gn1-8		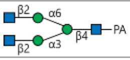	5.1	3.3	5.2	Hex3HexNAc3-PA	Sialylglycopeptide, glycosidase
Gn1-9		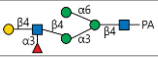	6.4	4.0	7.3	Hex4HexNAc2dHex1-PA	Human plasma-α1-AGP, glycosidase
Gn1-10		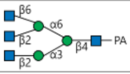	5.8	2.8	3.3	Hex3HexNAc4-PA	Mouse serum, hydrazinolysis-peeling, glycosidase
Gn1-11		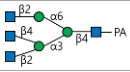	5.5	4.6	11.4	Hex3HexNAc4-PA	Human plasma-α1-AGP, glycosidase
Gn1-12	sC-1	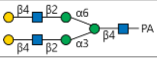	7.0	4.0	7.6	Hex5HexNAc3-PA	Sialylglycopeptide, glycosidase
Gn1-13		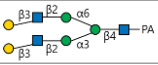	6.8	4.1	7.8	Hex5HexNAc3-PA	Mouse serum, hydrazinolysis-peeling, glycosidase
Gn1-14	sE-23	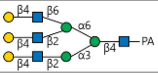	8.6	3.6	5.9	Hex6HexNAc4-PA	Mouse serum, hydrazinolysis-peeling, glycosidase
Gn1-15	sE-21	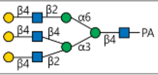	8.3	5.6	16.7	Hex6HexNAc4-PA	Human plasma-α1-AGP, glycosidase
Gn1-16	sE-22	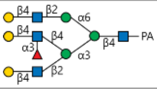	9.1	5.5	16.3	Hex6HexNAc4dHex1-PA	Human plasma-α1-AGP, glycosidase

Table B. (continued)

Standard glycan #	Correspond-ing glycan # [a]	Structure [b]	NP-GU Std. [c]	RP-GU Std. [d]	R-value Std. [e]	Composition	Notes (source, treatments, etc.)
**Gn1-core Acidic**							
Gn1-17	6N-LnM2A'	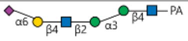	5.0	...	...	Hex3HexNAc2NeuAc1-PA	Sialylglycopeptide, glycosidase
Gn1-18	6N-LnM2B'	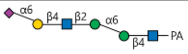	5.3	...	...	Hex3HexNAc2NeuAc1-PA	Sialylglycopeptide, glycosidase
Gn1-19	3N-LnM2A'	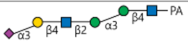	4.7	...	...	Hex3HexNAc2NeuAc1-PA	Urinary free-glycan [s2]
Gn1-20		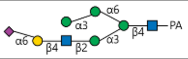	6.8	5.1	14.2	Hex5HexNAc2NeuAc1-PA	Bovine milk lactoferrin, glycosidase
Gn1-21		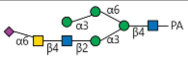	6.5	5.5	16.1	Hex4HexNAc3NeuAc1-PA	Bovine milk lactoferrin, glycosidase
Gn1-22	15	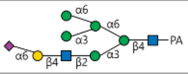	7.7	4.8	12.9	Hex6HexNAc2NeuAc1-PA	Bovine milk lactoferrin, glycosidase
Gn1-23		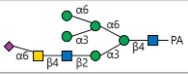	7.3	5.3	15.1	Hex5HexNAc3NeuAc1-PA	Bovine milk lactoferrin, glycosidase
Gn1-24	5	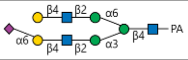	7.4	5.7	17.5	Hex5HexNAc3NeuAc1-PA	Sialylglycopeptide, glycosidase
Gn1-25	6	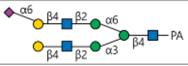	7.4	6.0	18.9	Hex5HexNAc3NeuAc1-PA	Sialylglycopeptide, glycosidase
Gn1-26	20	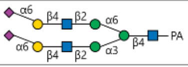	7.8	8.0	27.0	Hex5HexNAc3NeuAc2-PA	Sialylglycopeptide, glycosidase
Gn1-27	9	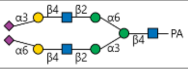	7.4	8.2	27.3	Hex5HexNAc3NeuAc2-PA	Urinary free-glycan [s2]
Gn1-28	12	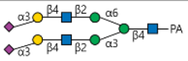	7.0	8.9	29.7	Hex5HexNAc3NeuAc2-PA	Urinary free-glycan [s2]
Gn1-29		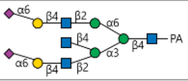	7.9	9.5	31.5	Hex5HexNAc4NeuAc2-PA	Human plasma-α1-AGP, glycosidase
Gn1-30	37	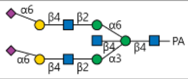	8.1	11.2	38.6	Hex5HexNAc4NeuAc2-PA	Human blood γ-globulin, hydrazinolysis-peeling
Gn1-31		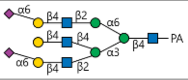	8.9	10.2	33.2	Hex6HexNAc4NeuAc2-PA	Human plasma-α1-AGP, glycosidase
Gn1-32		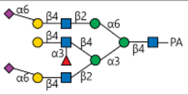	9.5	9.8	32.0	Hex6HexNAc4dHex1NeuAc2-PA	Human plasma-α1-AGP, glycosidase
Gn1-33	53	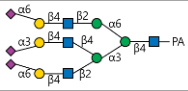	8.7	12.8	38.3	Hex6HexNAc4NeuAc3-PA	Human plasma-α1-AGP, glycosidase
Gn1-34	54	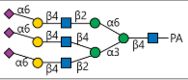	9.1	13.4	39.4	Hex6HexNAc4NeuAc3-PA	Human plasma-α1-AGP, glycosidase
Gn1-35	52	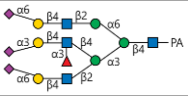	9.3	11.9	36.7	Hex6HexNAc4dHex1NeuAc3-PA	Human plasma-α1-AGP, glycosidase
Gn1-36		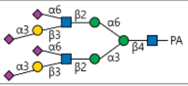	7.1	14.0	40.3	Hex5HexNAc3NeuAc4-PA	Bovine serum, hydrazinolysis-peeling
**Lactose-core**							
Lac-2		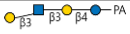	3.7	...	...	Hex3HexNAc1-PA	Takara Bio
Lac-3		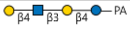	3.7	...	...	Hex3HexNAc1-PA	Takara Bio
Lac-4		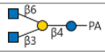	3.7	2.7	3.2	Hex2HexNAc2-PA	Carbosynth, glycosidase
Lac-5		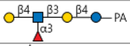	4.5	...	...	Hex3HexNAc1dHex1-PA	Takara Bio
Lac-6		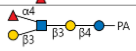	4.5	...	...	Hex3HexNAc1dHex1-PA	Takara Bio

Table B. (continued)

Standard glycan #	Corresponding glycan # [a]	Structure [b]	NP-GU Std. [c]	RP-GU Std. [d]	R-value Std. [e]	Composition	Notes (source, treatments, etc.)
Lac-7		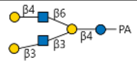	5.6	3.5	5.6	Hex4HexNAc2-PA	IsoSep AB, glycosidase
Lac-8		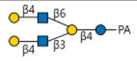	5.6	3.4	5.4	Hex4HexNAc2-PA	Carbosynth
Lac-9		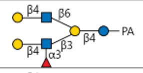	6.4	3.0	4.0	Hex4HexNAc2dHex1-PA	IsoSep AB, glycosidase
Lac-10		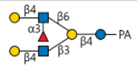	6.3	3.3	5.1	Hex4HexNAc2dHex1-PA	IsoSep AB, glycosidase
Lac-11		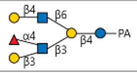	6.5	3.0	3.9	Hex4HexNAc2dHex1-PA	IsoSep AB, glycosidase
Lac-12		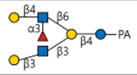	6.2	3.3	5.1	Hex4HexNAc2dHex1-PA	IsoSep AB, glycosidase
Lac-13		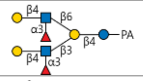	7.2	2.9	3.6	Hex4HexNAc2dHex2-PA	IsoSep AB
Lac-14		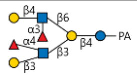	7.2	2.8	3.5	Hex4HexNAc2dHex2-PA	IsoSep AB
Lac-15		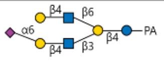	6.1	5.2	15.2	Hex4HexNAc2dHex1NeuAc1-PA	Urinary free-glycan [s2]
Lac-16		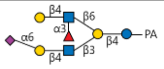	6.8	5.1	14.7	Hex4HexNAc2NeuAc1-PA	Urinary free-glycan [s2]
Lac-17		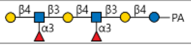	7.0	...	...	Hex4HexNAc2dHex2-PA	Glycosphingolipids of human colon cancer [s3]
Lac-18		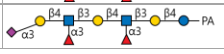	7.0	...	...	Hex4HexNAc2dHex2NeuAc1-PA	Glycosphingolipids of human colon cancer [s3]

a) Glycans corresponded with the standard glycans are indicated. Glycan numbers are from Fig 1, Supporting information, Fig A, C–E (S2 File).

b) Monosaccharide symbols are according to the symbol nomenclature for glycans (SNFG), and indicated as follows: blue circle, Glc; blue square, GlcNAc; green circle, Man; green square, ManNAc; yellow circle, Gal; yellow square, GalNAc; red triangle, Fuc; purple diamond, Sialic acid (NeuAc); circled "P", phosphate; boxed "Ac", acetate.

c) Calculated values from elution times of normal phase HPLC.

d) Calculated values from elution times of reversed phase HPLC.

e) Calculated values from elution times of reversed phase HPLC.

c–e) Representative data are shown.

f) "..." indicates no corresponding data are available.

Table C. Settings of SRM measurements of the PA-glycans by scheduled MRM mode

Targeted glycans #	Precursor (Q1) *m/z*	Ion species	Product (Q3) *m/z*	Ion species	CE (eV)	Q1 Resolution	Q3 Resolution	Retention time(min)	Window (sec)	Weight
1	778.26	+2H	503.23	+H	50	Low	Unit	6.00	150	3.0
1	778.26	+2H	405.08	+H	20	Low	Unit	6.00	150	3.0
**1**	**778.26**	**+2H**	**827.34**	**+H**	**25**	**Low**	**Low**	**6.00**	**150**	**3.0**
31	985.37	+2H	1604.59	+H	25	Low	Low	6.35	150	3.0
**31**	**985.37**	**+2H**	**1313.50**	**+H**	**35**	**Low**	**Low**	**6.35**	**150**	**3.0**
3, 15	883.83	+2H	624.26	+H	50	Low	Unit	6.48	177	3.0
**3, 15**	**883.83**	**+2H**	**1110.42**	**+H**	**30**	**Low**	**Low**	**6.48**	**177**	**3.0**
32	977.37	+2H	1151.45	+H	40	Low	Low	6.70	150	3.0
**32**	**977.37**	**+2H**	**1297.50**	**+H**	**30**	**Low**	**Low**	**6.70**	**150**	**3.0**
2	867.83	+2H	786.31	+H	25	Low	Unit	7.10	150	3.0
**2**	**867.83**	**+2H**	**1314.48**	**+H**	**15**	**Low**	**Low**	**7.10**	**150**	**3.0**
49	1086.91	+2H	1151.43	+H	55	Low	Low	7.25	150	3.0
**49**	**1086.91**	**+2H**	**1516.58**	**+H**	**35**	**Low**	**Low**	**7.25**	**150**	**3.0**
4	867.83	+2H	786.31	+H	40	Low	Unit	7.60	150	3.0
**4**	**867.83**	**+2H**	**1223.47**	**+H**	**25**	**Low**	**Low**	**7.60**	**150**	**3.0**
5, 6	904.34	+2H	624.26	+H	50	Low	Unit	7.75	282	1.0
**5, 6**	**904.34**	**+2H**	**1151.45**	**+H**	**30**	**Low**	**Low**	**7.75**	**282**	**1.0**
8	1013.38	+2H	421.18	+H	60	Low	Unit	7.80	150	3.0
**8**	**1013.38**	**+2H**	**1223.47**	**+H**	**25**	**Low**	**Low**	**7.80**	**150**	**3.0**
6N-LnM2A′, 3N-LnM2A′	640.75	+2H	624.26	+H	20	Low	Unit	8.10	150	1.0
**6N-LnM2A′, 3N-LnM2A′**	**640.75**	**+2H**	**989.39**	**+H**	**25**	**Low**	**Low**	**8.10**	**150**	**1.0**
33, 34	1122.92	+2H	624.26	+H	45	Low	Unit	8.35	150	3.0
**33, 34**	**1122.92**	**+2H**	**1588.60**	**+H**	**30**	**Low**	**Low**	**8.35**	**150**	**3.0**
9, 12, 20	1049.89	+2H	624.26	+H	60	Low	Unit	8.90	180	1.0
**9, 12, 20**	**1049.89**	**+2H**	**1442.54**	**+H**	**35**	**Low**	**Low**	**8.90**	**180**	**1.0**
10, 21	1070.40	+2H	1442.54	+H	30	Low	Low	9.28	159	3.0
**10, 21**	**1070.40**	**+2H**	**698.26**	**+H**	**30**	**Low**	**Unit**	**9.28**	**159**	**3.0**
**11**	**1090.91**	**+2H**	**1483.57**	**+H**	**30**	**Low**	**Low**	**9.30**	**150**	**3.0**
11	1090.91	+2H	698.26	+H	30	Low	Unit	9.30	150	3.0
50	1106.38	+2H	827.34	+H	55	Low	**Low**	9.90	150	3.0
**50**	**1106.38**	**+2H**	**1555.52**	**+H**	**30**	**Low**	**Low**	**9.90**	**150**	**3.0**
52	967.69	+3H	624.26	+H	55	Low	Unit	10.40	150	3.0
**52**	**967.69**	**+3H**	**1305.48**	**+2H**	**20**	**Low**	**Low**	**10.40**	**150**	**3.0**
23, 37	1151.43	+2H	462.21	+H	70	Low	Low	11.13	225	3.0
**23, 37**	**767.95**	**+3H**	**1005.88**	**+2H**	**15**	**Low**	**Low**	**11.13**	**225**	**3.0**
37, 53, 54	919.00	+3H	624.26	+H	55	Low	Unit	11.43	255	3.0
**37, 53, 54**	**919.00**	**+3H**	**1049.89**	**+2H**	**20**	**Low**	**Low**	**11.43**	**255**	**3.0**
51	792.63	+3H	503.23	+H	50	Low	Unit	11.95	150	3.0
**51**	**792.63**	**+3H**	**1005.88**	**+2H**	**15**	**Low**	**Low**	**11.95**	**150**	**3.0**
**73**	**1205.44**	**+3H**	**1479.54**	**+2H**	**20**	**Low**	**Low**	**13.70**	**150**	**3.0**
73	904.33	+4H	1108.41	+3H	15	Low	Low	13.70	150	3.0
56,59	986.70	+3H	462.21	+H	65	Low	**Low**	14.28	213	3.0
**56,59**	**986.70**	**+3H**	**1333.99**	**+2H**	**20**	**Low**	**Low**	**14.28**	**213**	**3.0**
**39, 40**	**1224.46**	**+2H**	**1645.62**	**+H**	**35**	**Low**	**Low**	**14.60**	**150**	**3.0**
39, 40	1224.46	+2H	803.29	+H	35	Low	Unit	14.60	150	3.0
24, 25	1005.88	+2H	827.34	+H	55	Low	Low	14.95	240	1.0
**24, 25**	**1005.88**	**+2H**	**1354.53**	**+H**	**35**	**Low**	**Low**	**14.95**	**240**	**1.0**
**75**	**1254.13**	**+3H**	**1552.57**	**+2H**	**20**	**Low**	**Low**	**15.20**	**150**	**3.0**
75	940.85	+4H	1157.09	+3H	15	Low	Low	15.20	150	3.0
D4-std	991.39	+2H	507.26	+H	60	Low	Unit	15.25	150	3.0
**D4-std**	**991.39**	**+2H**	**1487.59**	**+H**	**25**	**Low**	**Low**	**15.25**	**150**	**3.0**
Ac-1	1070.89	+2H	1151.43	+H	45	Low	Low	15.70	150	3.0
**Ac-1**	**1070.89**	**+2H**	**699.25**	**+H**	**30**	**Low**	**Unit**	**15.70**	**150**	**3.0**
**74**	**1084.07**	**+3H**	**1224.46**	**+2H**	**20**	**Low**	**Low**	**16.05**	**150**	**3.0**
74	1084.07	+3H	803.29	+H	25	Low	Unit	16.05	150	3.0
13, 27, 42	1151.43	+2H	827.34	+H	60	Low	Unit	16.43	249	1.0
**13, 27, 42**	**1151.43**	**+2H**	**1645.62**	**+H**	**35**	**Low**	**Low**	**16.43**	**249**	**1.0**

Table C. (continued)

Targeted glycans #	Precursor (Q1) *m/z*	Ion species	Product (Q3) *m/z*	Ion species	CE (eV)	Q1 Resolution	Q3 Resolution	Retention time(min)	Window (sec)	Weight
60	889.67	+3H	503.23	+H	55	Low	Unit	17.30	150	3.0
**60**	**889.67**	**+3H**	**1151.43**	**+2H**	**20**	**Low**	**Low**	**17.30**	**150**	**3.0**
62	1035.38	+3H	1354.53	+H	45	Low	Low	17.30	150	3.0
**62**	**1035.38**	**+3H**	**1151.43**	**+2H**	**20**	**Low**	**Low**	**17.30**	**150**	**3.0**
**77**	**1021.71**	**+3H**	**1203.94**	**+2H**	**20**	**Low**	**Low**	**17.55**	**150**	**5.0**
77	1021.71	+3H	1313.50	+H	45	Low	Low	17.55	150	5.0
**57, 61, (72)**	**938.35**	**+3H**	**1261.48**	**+2H**	**15**	**Low**	**Low**	**17.60**	**282**	**3.0**
**72, (57, 61)**	**938.35**	**+3H**	**1151.43**	**+2H**	**20**	**Low**	**Low**	**17.60**	**282**	**3.0**
Ac-2, Ac-3	781.96	+3H	1026.89	+2H	15	Low	Low	18.00	192	3.0
**Ac-2, Ac-3**	**781.96**	**+3H**	**408.15**	**+H**	**30**	**Low**	**Unit**	**18.00**	**192**	**3.0**
28, 29, 41	1078.91	+2H	446.21	+H	55	Low	Unit	18.13	243	1.0
**28, 29, 41**	**1078.91**	**+2H**	**1500.58**	**+H**	**30**	**Low**	**Low**	**18.13**	**243**	**1.0**
Ac-4	1070.89	+2H	828.32	+H	60	Low	Low	18.15	150	3.0
**Ac-4**	**1070.89**	**+2H**	**1484.55**	**+H**	**30**	**Low**	**Low**	**18.15**	**150**	**3.0**
65, 66	986.70	+3H	1354.53	+H	40	Low	Low	18.25	204	3.0
**65, 66**	**986.70**	**+3H**	**1151.43**	**+2H**	**20**	**Low**	**Low**	**18.25**	**204**	**3.0**
30, 43, 44	1224.46	+2H	446.21	+H	65	Low	Unit	19.03	261	1.0
**30, 43, 44**	**1224.46**	**+2H**	**1791.68**	**+H**	**35**	**Low**	**Low**	**19.03**	**261**	**1.0**
78	1084.07	+3H	446.21	+H	60	Low	Unit	19.65	150	3.0
**78**	**1084.07**	**+3H**	**1224.46**	**+2H**	**20**	**Low**	**Low**	**19.65**	**150**	**3.0**
64	938.35	+3H	446.21	+H	50	Low	Unit	19.65	150	3.0
**64**	**938.35**	**+3H**	**1224.46**	**+2H**	**20**	**Low**	**Low**	**19.65**	**150**	**3.0**
46	787.30	+3H	1176.48	+H	25	Low	Low	20.80	150	3.0
**46**	**787.30**	**+3H**	**997.88**	**+2H**	**15**	**Low**	**Low**	**20.80**	**150**	**3.0**
48, 67, 68, 79	1035.38	+3H	446.21	+H	60	Low	Unit	21.15	258	3.0
**48, 67, 68, 79**	**1035.38**	**+3H**	**1224.46**	**+2H**	**20**	**Low**	**Low**	**21.15**	**258**	**3.0**
47	884.33	+3H	1176.48	+H	30	Low	Low	21.35	150	3.0
**47**	**884.33**	**+3H**	**1180.45**	**+2H**	**20**	**Low**	**Low**	**21.35**	**150**	**3.0**
14	733.28	+3H	1176.48	+H	25	Low	Low	22.15	150	3.0
**14**	**733.28**	**+3H**	**997.88**	**+2H**	**15**	**Low**	**Low**	**22.15**	**150**	**3.0**
69	1006.04	+3H	446.21	+H	50	Low	Unit	23.30	150	3.0
**69**	**1006.04**	**+3H**	**1180.45**	**+2H**	**20**	**Low**	**Low**	**23.30**	**150**	**3.0**
**Ac-5**	**1172.43**	**+2H**	**699.25**	**+H**	**35**	**Low**	**Unit**	**23.33**	**225**	**3.0**
Ac-5	1172.43	+2H	1687.63	+H	35	Low	Low	23.33	225	3.0
80	918.84	+4H	731.27	+H	25	Low	Low	23.95	150	3.0
**80**	**918.84**	**+4H**	**1127.75**	**+3H**	**15**	**Low**	**Low**	**23.95**	**150**	**3.0**
70, 81	1103.08	+3H	1326.00	+2H	20	Low	Low	24.23	207	3.0
**70, 81**	**827.56**	**+4H**	**1006.04**	**+3H**	**15**	**Low**	**Low**	**24.23**	**207**	**3.0**

Mass values of Q1 (precursor) and Q3 (product) ions, collision energy (CE), resolutions of ion isolation, expected elution time on reversed phase HPLC with formic acid eluents, transition window, and transition weighting values are shown, respectively. Declustering potential (DP), Entrance potential (EP) and Collision cell exit potential were fixed values at 40, 10 and 15, respectively. Minimum and maximum dwell time were set to 10 ms and 50 ms, respectively. Target scan time was set for 1 sec. Glycans are sorted by elution time. Structurally similar isomers were measured at the same transition, and the average of position of these glycans was used for the elution time setting. For most glycans and groups of glycans, measurements were performed by multiple transitions. One such measurement that was used for quantification is shown in bold and colored.

Table D. Proposed structures of urinary free-glycans found in this study

Glycan #[a]	Proposed Structure [b]	NP-GU Exp.[c]	NP-GU Std.	RP-GU Exp.[d]	RP-GU Std.	*R* value Exp.[e]	*R* value Std.	Mass (*m/z*) Exp.[f]	Mass (*m/z*) Theo.	Ion species	Estimated Composition	Note
1	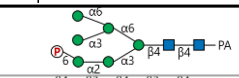	7.2	...[g]	4.5	...[g]	10.9	...[g]	777.97	778.26	M+2H	Hex6HexNAc2Phosphate1-PA	Free-*N*-glycan, Oligomannose, Gn2
2	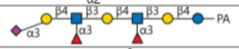	7.0	7.0	5.2	...	14.7	...	579.04	578.89	M+3H	Hex4HexNAc2dHex2NeuAc1-PA	Lactose-core glycan
3	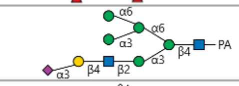	7.2	...	5.3	...	15.3	...	883.96	883.83	M+2H	Hex6HexNAc2NeuAc1-PA	Free-*N*-glycan, Hybrid, Gn1
4	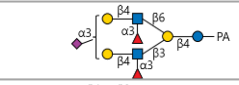	7.2	...	5.4	...	15.8	...	868.06	867.83	M+2H	Hex4HexNAc2dHex2NeuAc1-PA	Lactose-core glycan
5	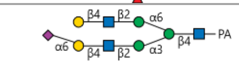	7.3	7.4	5.7	5.7	17.5	17.5	904.23	904.34	M+2H	Hex5HexNAc3NeuAc1-PA	Free-*N*-glycan, Complex, Bi-antennary, Gn1
6	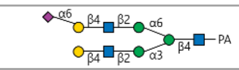	7.4	7.4	6.0	6.0	18.8	18.9	904.21	904.34	M+2H	Hex5HexNAc3NeuAc1-PA	Free-*N*-glycan, Complex, Bi-antennary, Gn1
7	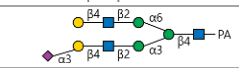	7.0	7.0	6.3	6.3	20.6	20.4	904.37	904.34	M+2H	Hex5HexNAc3NeuAc1-PA	Free-*N*-glycan, Complex, Bi-antennary, Gn1
8	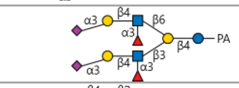	7.0	...	7.1	...	24.1	...	1013.24	1013.38	M+2H	Hex4HexNAc2dHex2NeuAc2-PA	Lactose-core glycan
9	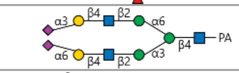	7.5	7.4	8.0	8.2	27.4	27.3	700.36	700.26	M+3H	Hex5HexNAc3NeuAc2-PA	Free-*N*-glycan, Complex, Bi-antennary, Gn1
10	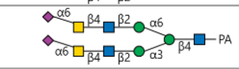	7.2	...	8.7	...	29.1	...	727.73	727.61	M+3H	Hex3HexNAc5NeuAc2-PA	Free-*N*-glycan, Complex, Bi-antennary, Gn1
11	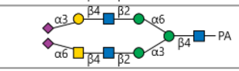	7.0	...	8.8	...	29.4	...	714.08	713.94	M+3H	Hex4HexNAc4NeuAc2-PA	Free-*N*-glycan, Complex, Bi-antennary, Gn1
12	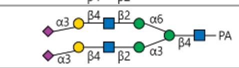	6.9	7.0	8.9	8.9	29.9	29.7	700.47	700.26	M+3H	Hex5HexNAc3NeuAc2-PA	Free-*N*-glycan, Complex, Bi-antennary, Gn1
13	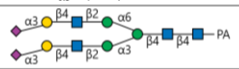	7.2	...	>20	...	52.3	...	768.29	767.95	M+3H	Hex5HexNAc4NeuAc2-PA	Free-*N*-glycan, Complex, Bi-antennary, Gn2
14	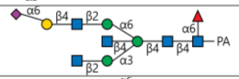	7.2	...	>20	...	58.2	...	733.35	733.28	M+3H	Hex4HexNAc5dHex1NeuAc1-PA	Free-*N*-glycan, Complex, Bi-antennary, Gn2
15	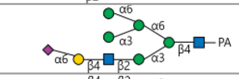	7.7	7.7	4.8	4.8	12.9	12.9	883.67	883.83	M+2H	Hex6HexNAc2NeuAc1-PA	Free-*N*-glycan, Hybrid, Gn1
16	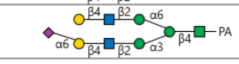	7.6	...	5.3	...	15.4	...	904.85	904.34	M+2H	Hex5HexNAc3NeuAc1-PA	Free-*N*-glycan, C-2 epimer of #5
17	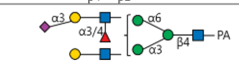	7.8	...	5.6	...	16.7	...	977.57	977.37	M+2H	Hex5HexNAc3dHex1NeuAc1-PA	Free-*N*-glycan, Complex, Bi-antennary, Gn1
18	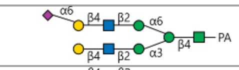	7.6	...	5.9	...	18.3	...	904.19	904.34	M+2H	Hex5HexNAc3NeuAc1-PA	Free-*N*-glycan, C-2 epimer of #6
19	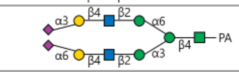	7.5	...	7.6	...	25.8	...	700.49	700.26	M+3H	Hex5HexNAc3NeuAc2-PA	Free-*N*-glycan, C-2 epimer of #9
20	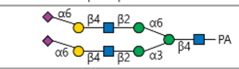	7.7	7.8	8.0	8.0	27.1	27.0	700.48	700.26	M+3H	Hex5HexNAc3NeuAc2-PA	Free-*N*-glycan, Complex, Bi-antennary, Gn1
21	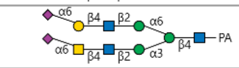	7.4	...	8.7	...	29.1	...	714.21	713.94	M+3H	Hex4HexNAc4NeuAc2-PA	Free-*N*-glycan, Complex, Bi-antennary, Gn1
22	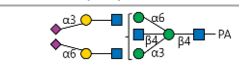	7.4	...	10.5	...	34.1	...	768.02	767.95	M+3H	Hex5HexNAc4NeuAc2-PA	Free-*N*-glycan, Complex, Bi-antennary, Gn1
23	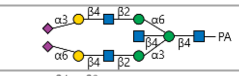	7.5	...	11.5	...	36.3	...	768.18	767.95	M+3H	Hex5HexNAc4NeuAc2-PA	Free-*N*-glycan, Complex, Bi-antennary, Gn1
24	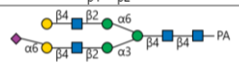	7.6	7.7	12.1	12.1	37.4	37.5	671.09	670.92	M+3H	Hex5HexNAc4NeuAc1-PA	Free-*N*-glycan, Complex, Bi-antennary, Gn2
25	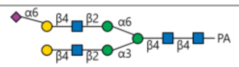	7.6	7.6	15.0	15.2	42.0	41.8	671.00	670.92	M+3H	Hex5HexNAc4NeuAc1-PA	Free-*N*-glycan, Complex, Bi-antennary, Gn2
26	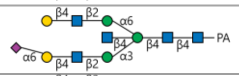	7.8	7.7	19.0	19.0	46.2	46.0	738.44	738.62	M+3H	Hex5HexNAc5NeuAc1-PA	Free-*N*-glycan, Complex, Bi-antennary, Gn2
27	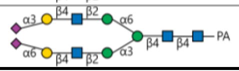	7.6	...	>20	...	47.4	...	768.08	767.95	M+3H	Hex5HexNAc4NeuAc2-PA	Free-*N*-glycan, Complex, Bi-antennary, Gn2

Table D. (continued)

Glycan #[a]	Proposed Structure [b]	NP-GU Exp.[c]	NP-GU Std.	RP-GU Exp.[d]	RP-GU Std.	R value Exp.[e]	R value Std.	Mass (m/z) Exp. [f]	Mass (m/z) Theo.	Ion species	Estimated Composition	Note
28	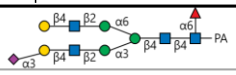	7.5	...	>20	...	49.7	...	1078.74	1078.91	M+2H	Hex5HexNAc4dHex1NeuAc1-PA	Free-*N*-glycan, Complex, Bi-antennary, Gn2
29	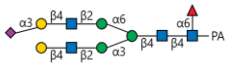	...	...	>20	...	49.7	...	...	...	...	Hex5HexNAc4dHex1NeuAc1-PA	Free-*N*-glycan, Complex, Bi-antennary, Gn2, Overlapped with #28, minor composition
30	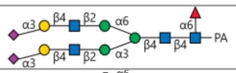	7.5	...	>20	...	59.1	...	816.75	816.64	M+3H	Hex5HexNAc4dHex1NeuAc2-PA	Free-*N*-glycan, Complex, Bi-antennary, Gn2
31	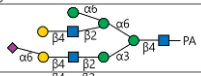	8.2	...	4.7	...	12.3	...	985.43	985.37	M+2H	Hex6HexNAc3NeuAc1-PA	Free-*N*-glycan, Hybrid, Gn1
32	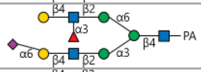	8.1	...	5.1	...	14.2	...	977.49	977.37	M+2H	Hex5HexNAc3dHex1NeuAc1-PA	Free-*N*-glycan, Complex, Bi-antennary, Gn1
33	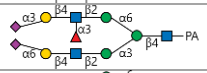	8.1	...	7.6	...	25.8	...	749.31	748.95	M+3H	Hex5HexNAc3dHex1NeuAc2-PA	Free-*N*-glycan, Complex, Bi-antennary, Gn1
34	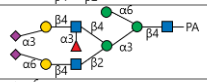	7.9	...	7.8	...	26.2	...	748.81	748.95	M+3H	Hex5HexNAc3dHex1NeuAc2-PA	Free-*N*-glycan, Complex, Bi-antennary, Gn1
35	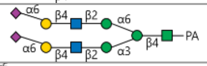	8.0	...	7.7	...	26.2	...	700.21	700.26	M+3H	Hex5HexNAc3NeuAc2-PA	Free-*N*-glycan, C-2 epimer of #20
36	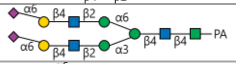	7.9	...	11.4	...	36.1	...	768.26	767.95	M+3H	Hex5HexNAc4NeuAc2-PA	Free-*N*-glycan, C-2 epimer of #42
37	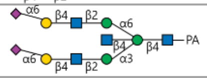	8.1	8.1	11.9	11.2	37.0	36.8	768.22	767.95	M+3H	Hex5HexNAc4NeuAc2-PA	Free-*N*-glycan, Complex, Bi-antennary, Gn1
38	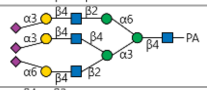	8.3	...	13.2	...	39.3	...	919.22	919.00	M+3H	Hex6HexNAc4NeuAc3-PA	Free-*N*-glycan, Complex, Tri-antennary, Gn1
39	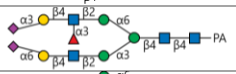	8.3	...	15.7	...	43.1	...	816.94	816.64	M+3H	Hex5HexNAc4dHex1NeuAc2-PA	Free-*N*-glycan, Complex, Bi-antennary, Gn2
40	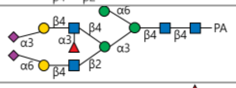	8.3	...	16.6	...	44.1	...	816.99	816.64	M+3H	Hex5HexNAc4dHex1NeuAc2-PA	Free-*N*-glycan, Complex, Bi-antennary, Gn2
41	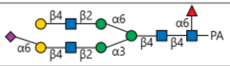	8.0	7.9	18.4	18.4	45.6	45.4	719.60	719.61	M+3H	Hex5HexNAc4dHex1NeuAc1-PA	Free-*N*-glycan, Complex, Bi-antennary, Gn2
42	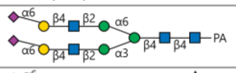	8.0	7.9	19.0	18.9	46.1	46.0	767.77	767.95	M+3H	Hex5HexNAc4NeuAc2-PA	Free-*N*-glycan, Complex, Bi-antennary, Gn2
43	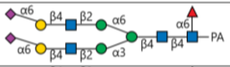	8.3	8.2	>20	24.5	52.1	52.0	816.83	816.64	M+3H	Hex5HexNAc4dHex1NeuAc2-PA	Free-*N*-glycan, Complex, Bi-antennary, Gn2
44	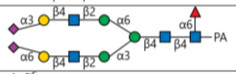	8.0	...	>20	...	54.7	...	816.80	816.64	M+3H	Hex5HexNAc4dHex1NeuAc2-PA	Free-*N*-glycan, Complex, Bi-antennary, Gn2
45	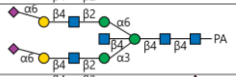	8.1	8.0	>20	27.1	55.1	54.9	835.85	835.65	M+3H	Hex5HexNAc5NeuAc2-PA	Free-*N*-glycan, Complex, Bi-antennary, Gn2
46	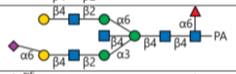	8.2	8.1	>20	27.2	55.2	55.0	787.35	787.30	M+3H	Hex5HexNAc5dHex1NeuAc1-PA	Free-*N*-glycan, Complex, Bi-antennary, Gn2
47	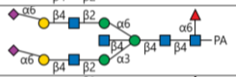	8.3	8.3	>20	32.7	61.4	61.2	884.60	884.33	M+3H	Hex5HexNAc5dHex1NeuAc2-PA	Free-*N*-glycan, Complex, Bi-antennary, Gn2
48	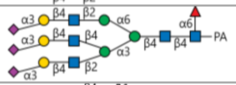	8.5	...	>20	...	71.3	...	1035.53	1035.38	M+3H	Hex6HexNAc5dHex1NeuAc3-PA	Free-*N*-glycan, Complex, Tri-antennary, Gn2
49	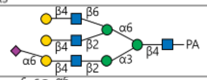	8.8	...	5.4	...	16.1	...	725.11	724.94	M+3H	Hex6HexNAc4NeuAc1-PA	Free-*N*-glycan, Complex, Tri-antennary, Gn1
50	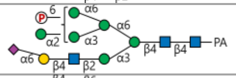	8.8	...	8.8	...	29.9	...	738.25	737.92	M+3H	Hex7HexNAc3NeuAc1-Phosphate1-PA	Free-*N*-glycan, Hybrid, Gn2
51	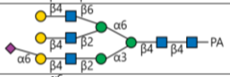	9.0	9.0	9.2	9.2	31.0	30.9	792.76	792.63	M+3H	Hex6HexNAc5NeuAc1-PA	Free-*N*-glycan, Complex, Tri-antennary, Gn2
52	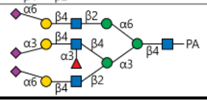	9.2	9.3	11.9	11.9	36.7	36.7	967.97	967.69	M+3H	Hex6HexNAc4dHex1NeuAc3-PA	Free-*N*-glycan, Complex, Tri-antennary, Gn1

Table D. (continued)

Glycan #[a]	Proposed Structure [b]	NP-GU Exp.[c]	NP-GU Std.	RP-GU Exp.[d]	RP-GU Std.	*R* value Exp.[e]	*R* value Std.	Mass (*m/z*) Exp.[f]	Mass (*m/z*) Theo.	Ion species	Estimated Composition	Note
53	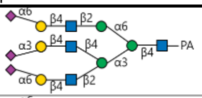	8.6	8.7	12.9	12.8	38.6	38.3	919.05	919.00	M+3H	Hex6HexNAc4NeuAc3-PA	Free-*N*-glycan, Complex, Tri-antennary, Gn1
54	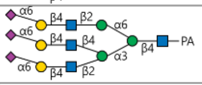	9.1	9.1	13.4	13.4	39.4	39.4	919.25	919.00	M+3H	Hex6HexNAc4NeuAc3-PA	Free-*N*-glycan, Complex, Tri-antennary, Gn1
55	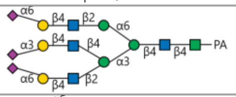	9.0	...	>20	...	47.5	...	986.90	986.70	M+3H	Hex6HexNAc5NeuAc3-PA	Free-*N*-glycan, C-2 epimer of #65
56	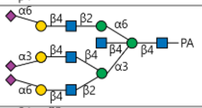	8.7	...	>20	...	47.6	...	986.62	986.70	M+3H	Hex6HexNAc5NeuAc3-PA	Free-*N*-glycan, Complex, Tri-antennary, Gn1
57	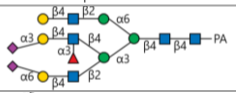	9.3	...	>20	...	48.2	...	938.04	938.35	M+3H	Hex6HexNAc5dHex1NeuAc2-PA	Free-*N*-glycan, Complex, Tri-antennary, Gn2
58	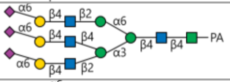	9.5	...	>20	...	49.4	...	986.35	986.70	M+3H	Hex6HexNAc5NeuAc3-PA	Free-*N*-glycan, C-2 epimer of #66
59	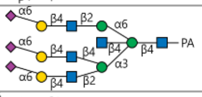	9.3	...	>20	...	49.7	...	986.88	986.70	M+3H	Hex6HexNAc5NeuAc3-PA	Free-*N*-glycan, Complex, Tri-antennary, Gn1
60	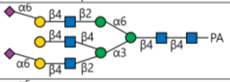	9.0	9.0	>20	>20	50.4	50.5	889.75	889.67	M+3H	Hex6HexNAc5NeuAc2-PA	Free-*N*-glycan, Complex, Tri-antennary, Gn2
61	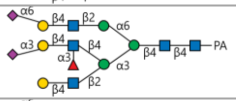	9.3	...	>20	...	55.3	...	937.84	938.35	M+3H	Hex6HexNAc5dHex1NeuAc2-PA	Free-*N*-glycan, Complex, Tri-antennary, Gn2
62	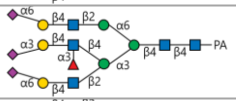	9.5	9.4	>20	27.1	55.3	55.1	1035.17	1035.38	M+3H	Hex6HexNAc5dHex1NeuAc3-PA	Free-*N*-glycan, Complex, Tri-antennary, Gn2
63	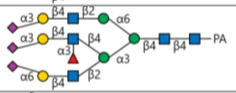	9.2	...	>20	...	56.4	...	1035.74	1035.38	M+3H	Hex6HexNAc5dHex1NeuAc3-PA	Free-*N*-glycan, Complex, Tri-antennary, Gn2
64	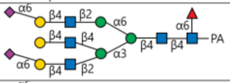	9.5	...	>20	...	56.6	...	938.48	938.35	M+3H	Hex6HexNAc5dHex1NeuAc2-PA	Free-*N*-glycan, Complex, Tri-antennary, Gn2
65	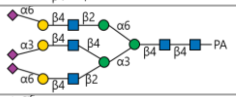	8.9	8.9	>20	28.7	57.2	57.0	986.92	986.70	M+3H	Hex6HexNAc5NeuAc3-PA	Free-*N*-glycan, Complex, Tri-antennary, Gn2
66	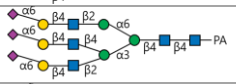	9.3	9.3	>20	30.9	59.5	59.3	986.84	986.70	M+3H	Hex6HexNAc5NeuAc3-PA	Free-*N*-glycan, Complex, Tri-antennary, Gn2
67	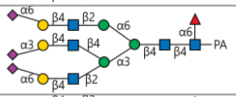	9.2	...	>20	...	63.2	...	1035.55	1035.38	M+3H	Hex6HexNAc5dHex1NeuAc3-PA	Free-*N*-glycan, Complex, Tri-antennary, Gn2
68	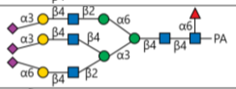	8.8	...	>20	...	65.6	...	1035.39	1035.38	M+3H	Hex6HexNAc5dHex1NeuAc3-PA	Free-*N*-glycan, Complex, Tri-antennary, Gn2
69	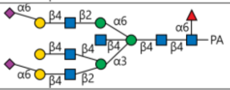	9.3	...	>20	...	66.7	...	1006.14	1006.04	M+3H	Hex6HexNAc6dHex1NeuAc2-PA	Free-*N*-glycan, Complex, Tri-antennary, Gn2
70	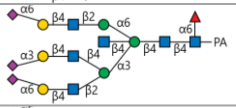	9.1	...	>20	...	73.0	...	1103.06	1103.08	M+3H	Hex6HexNAc6dHex1NeuAc3-PA	Free-*N*-glycan, Complex, Tri-antennary, Gn2
71	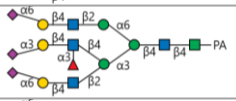	9.5	...	18.0	...	45.3	...	1035.52	1035.38	M+3H	Hex6HexNAc5dHex1NeuAc3-PA	Free-*N*-glycan, C-2 epimer of #62
72	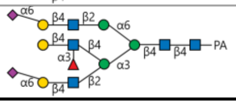	9.6	9.7	>20	21.3	48.7	48.7	938.50	938.35	M+3H	Hex6HexNAc5dHex1NeuAc2-PA	Free-*N*-glycan, Complex, Tri-antennary, Gn2

Table D. (continued)

Glycan #[a]	Proposed Structure [b]	NP-GU Exp.[c]	NP-GU Std.	RP-GU Exp.[d]	RP-GU Std.	*R* value Exp.[e]	*R* value Std.	Mass (*m/z*) Exp.[f]	Mass (*m/z*) Theo.	Ion species	Estimated Composition	Note
73	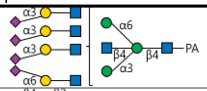	9.5	...	>20	...	51.4	...	1205.74	1205.44	M+3H	Hex7HexNAc6NeuAc4-PA	Free-*N*-glycan, Complex, Tetra-antennary, Gn1
74	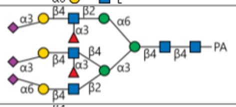	9.8	...	>20	...	51.7	...	1084.26	1084.07	M+3H	Hex6HexNAc5dHex2NeuAc3-PA	Free-*N*-glycan, Complex, Tri-antennary, Gn2
75	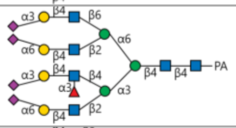	10.4	10.4	>20	28.7	53.8	53.8	940.55	940.85	M+4H	Hex7HexNAc6dHex1NeuAc4-PA	Free-*N*-glycan, Complex, Tetra-antennary,Gn2
76	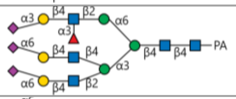	9.8	...	>20	...	56.1	...	1035.01	1035.38	M+3H	Hex6HexNAc5dHex1NeuAc3-PA	Free-*N*-glycan, Complex, Tri-antennary, Gn2
77	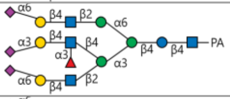	9.8	...	>20	...	56.8	...	1022.04	1021.71	M+3H	Hex7HexNAc4dHex1NeuAc3-PA	Free-*N*-glycan, Complex, Tri-antennary, Glcβ1-4GlcNAc
78	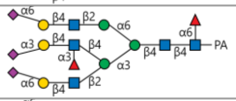	9.7	...	>20	...	61.0	...	1084.13	1084.07	M+3H	Hex6HexNAc5dHex2NeuAc3-PA	Free-*N*-glycan, Complex, Tri-antennary, Gn2
79	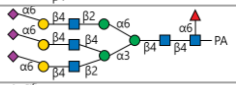	9.6	...	>20	...	65.4	...	1035.67	1035.38	M+3H	Hex6HexNAc5dHex1NeuAc3-PA	Free-*N*-glycan, Complex, Tri-antennary, Gn2
80	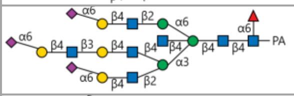	10.7	...	>20	...	72.6	...	919.21	918.84	M+4H	Hex7HexNAc7dHex1NeuAc3-PA	Free-*N*-glycan, Complex, Tri-antennary, Gn2
81	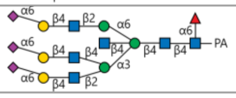	9.5	...	>20	...	75.9	...	1103.14	1103.08	M+3H	Hex6HexNAc6dHex1NeuAc3-PA	Free-*N*-glycan, Complex, Tri-antennary, Gn2
Ac-1	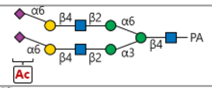	7.2	...	19.9	...	47.4	...	714.53	714.26	M+3H	Hex5HexNAc3NeuAc2Ac1-PA	Free-*N*-glycan, *O*-acetylated #20
Ac-2	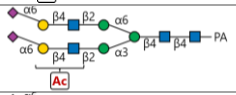	7.3	...	>20	...	49.5	...	782.10	781.96	M+3H	Hex5HexNAc4NeuAc2Ac1-PA	Free-*N*-glycan, *O*-acetylated #42
Ac-3	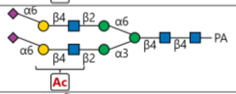	7.1	...	>20	...	50.8	...	782.19	781.96	M+3H	Hex5HexNAc4NeuAc2Ac1-PA	Free-*N*-glycan, *O*-acetylated #42
Ac-4	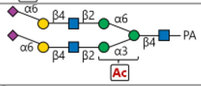	7.1	...	>20	...	52.1	...	714.57	714.26	M+3H	Hex5HexNAc3NeuAc2Ac1-PA	Free-*N*-glycan, *O*-acetylated #20
Ac-5	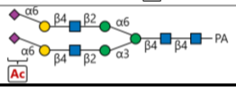	7.0	...	>20	...	65.9	...	782.07	781.96	M+3H	Hex5HexNAc4NeuAc2Ac1-PA	Free-*N*-glycan, *O*-acetylated #42

a) Glycan numbers are from Fig 1.

b) Monosaccharide symbols are according to the symbol nomenclature for glycans (SNFG), and indicated as follows: blue circle, Glc; blue square, GlcNAc; green circle, Man; green square, ManNAc; yellow circle, Gal; yellow square, GalNAc; red triangle, Fuc; purple diamond, Sialic acid (NeuAc); circled "P", phosphate; boxed "Ac", acetate.

c) Calculated values from elution times of normal phase HPLC.

d) Calculated values from elution times of reversed phase HPLC.

e) Calculated values from elution times of reversed phase HPLC.

f) Data obtained by LTQ-XL mass spectrometer.

c–f) Representative data are shown.

g) "..." indicates no corresponding data are available.

Table E. Sialic acid-linkage composition of tri-/tetra-antennary glycans

	Linkage composition[b]		Mass (*m/z*)		
Glycan #[a]	α2,3 (MA)	α2,6 (iPA)	Exp.[c]	Theo.	Ion species
38	2	1	941.96	941.38	M+3H
48	3	0	1048.82	1048.41	M+3H
52	1	2	999.26	999.41	M+3H
53	1	2	951.05	950.72	M+3H
54	0	3	960.57	960.07	M+3H
56	1	2	1018.77	1018.42	M+3H
57	1	1	956.52	956.38	M+3H
58	3	0	1027.40	1027.76	M+3H
61	1	1	956.83	956.38	M+3H
62	1	2	1067.72	1067.10	M+3H
63	2	1	1057.80	1057.76	M+3H
64	0	2	965.40	965.73	M+3H
65	1	2	1018.41	1018.42	M+3H
66	0	3	1027.46	1027.76	M+3H
67	1	2	1067.52	1067.10	M+3H
68	2	1	1058.24	1057.76	M+3H
69	0	2	1033.61	1033.42	M+3H
70	1	2	1135.12	1134.80	M+3H
71	1	2	1067.43	1067.10	M+3H
72	0	2	965.55	965.73	M+3H
73	3	1	1232.73	1232.16	M+3H
74	2	1	1106.41	1106.44	M+3H
75	2	2	1290.57	1290.19	M+3H
76	1	2	1067.25	1067.10	M+3H
77	1	2	1053.96	1053.43	M+3H
78	1	2	1115.93	1115.79	M+3H
79	0	3	1076.82	1076.45	M+3H
80	0	3	950.08	949.64	M+4H
81	0	3	1144.43	1144.14	M+3H

a) Glycan numbers are from Fig 1.

b) The linkage compositions by sialic acid linkage-specific alkylamidation. The amidation with methylamine (MA) and isopropylamine (iPA) were assigned as α2,3-linakge and α2,6-linakge, respectively.

c) Data obtained by LTQ-XL mass spectrometer.

Table F. Comparison between cancer patient groups and normal controls for each glycan level

Glycan #	Proposed Structures	Gastric cancer Fold change[b]	Gastric cancer *p*-values[a]	Pancreatic cancer Fold change[b]	Pancreatic cancer *p*-values[a]	Cholangiocacinoma Fold change[b]	Cholangiocacinoma *p*-values[a]	colorectal cancer Fold change[b]	colorectal cancer *p*-values[a]
1	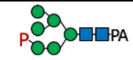	1.5	0.0177	1.1	0.2008	1.3	0.0814	1.1	0.5022
2	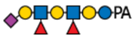	1.9	0.0751	2.4	0.0004	2.4	0.0568	1.9	0.0254
3	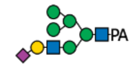	1.4	0.0886	1.4	0.0077	1.8	0.0060	1.9	0.0889
4	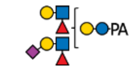	1.7	0.0270	2.5	< 0.0001	2.6	0.0149	1.9	0.0213
5	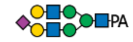	1.6	0.0100	1.6	0.0042	3.4	0.0028	3.9	0.0007
6	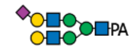	1.5	0.0270	1.3	0.0036	2.5	0.0019	2.7	0.0053
8	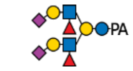	1.9	0.0061	2.3	0.0026	2.1	0.0191	1.6	0.0620
9	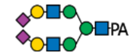	2.2	0.0011	1.6	< 0.0001	3.2	0.0002	3.0	0.0027
10	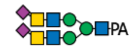	5.9	< 0.0001	1.5	0.0066	4.3	0.0002	1.7	0.0059
11	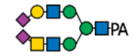	5.5	0.0126	1.3	0.0793	1.6	0.0060	1.5	0.0134
12	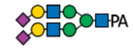	1.2	0.9801	1.1	0.4906	1.5	0.1134	1.4	0.2777
13	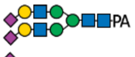	1.2	0.4692	1.1	0.6303	1.0	0.6515	0.9	0.0417
14	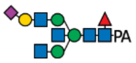	1.7	0.0005	1.5	0.0057	1.9	0.0191	1.8	0.0030
15	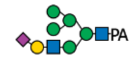	2.2	0.0009	1.7	< 0.0001	2.8	0.0002	2.1	0.0061
20	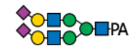	2.3	0.0007	1.8	< 0.0001	3.3	0.0002	3.4	0.0006
21	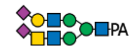	4.1	0.0529	1.4	0.0077	2.4	0.0084	1.7	0.0150
23	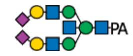	8.3	0.0003	1.5	0.0793	2.2	0.0011	1.3	0.1592
24	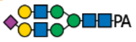	1.2	0.2617	1.3	0.0116	1.3	0.2615	1.5	0.0019
25	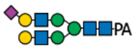	1.3	0.0751	1.3	0.0132	1.3	0.5431	1.6	0.0009
27	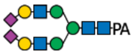	1.6	0.0069	1.4	0.0009	1.5	0.2032	1.3	0.0388
28	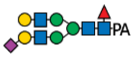	1.0	0.6585	1.0	0.9791	1.1	0.6945	0.9	0.4134
29	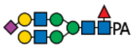	1.1	0.9217	1.1	0.1352	1.2	0.2952	1.2	0.3383
30	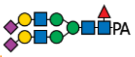	1.2	0.1992	1.1	0.1723	1.1	0.9146	0.9	0.1311
31	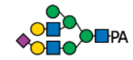	2.3	0.0100	1.6	0.0311	4.0	0.0307	5.0	0.0007
32	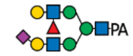	2.1	< 0.0001	2.0	0.0003	5.1	0.0002	5.5	0.0002
33+34	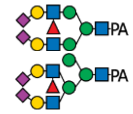	3.2	< 0.0001	2.0	0.0194	3.9	0.0011	3.8	0.0016
37	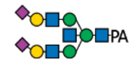	2.7	0.0001	1.5	0.0013	2.2	0.0019	1.6	0.0003
38	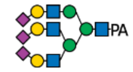	2.3	0.0002	1.7	0.0001	1.9	0.0002	1.3	0.0162
39+40	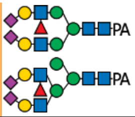	3.4	0.0002	1.9	0.0871	2.8	0.0028	2.9	0.0059

Table F. (continued)

Glycan #	Proposed Structures	Gastric cancer Fold change[b]	Gastric cancer *p*-values[a]	Pancreatic cancer Fold change[b]	Pancreatic cancer *p*-values[a]	Cholangiocacinoma Fold change[b]	Cholangiocacinoma *p*-values[a]	colorectal cancer Fold change[b]	colorectal cancer *p*-values[a]
41	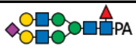	1.3	0.0401	1.3	0.0088	1.6	0.0084	1.5	0.0277
42	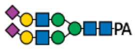	1.5	0.0089	1.4	0.0003	1.5	0.0060	1.3	0.0213
43	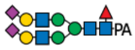	1.8	0.0011	1.6	0.0002	2.1	0.0568	1.7	0.0024
44	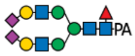	1.6	0.0002	1.5	0.0001	1.7	0.0028	1.4	0.0456
46	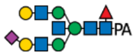	1.7	0.0011	1.5	0.0101	1.9	0.0191	1.8	0.0034
47	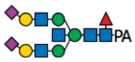	2.4	< 0.0001	1.8	0.0036	2.3	0.0060	2.1	0.0011
48	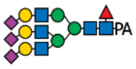	1.1	0.0961	1.1	0.5725	1.0	0.4515	0.7	0.0002
49	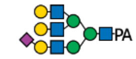	1.5	0.0158	1.4	0.0116	3.3	0.0003	3.9	0.0034
50	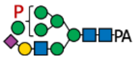	3.1	0.0011	1.9	< 0.0001	2.1	0.0112	2.1	0.0081
51	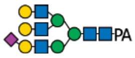	1.3	0.1307	1.4	0.0116	1.5	0.0112	1.4	0.0134
52	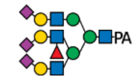	3.9	< 0.0001	2.2	0.0101	3.4	0.0006	3.5	0.0010
53	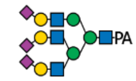	2.4	0.0270	1.9	0.0311	2.0	0.1134	1.1	0.5226
54	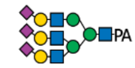	3.1	< 0.0001	2.0	0.0004	2.5	0.0060	2.3	0.0003
56	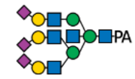	2.9	0.0011	1.5	0.0048	1.5	0.1504	1.3	0.0251
57	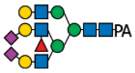	3.9	< 0.0001	2.3	0.0004	2.7	0.0028	3.7	< 0.0001
59	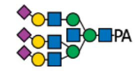	3.3	< 0.0001	1.7	0.0116	2.2	0.0084	2.0	0.0004
60	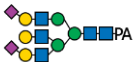	1.1	0.6416	1.5	0.0720	1.1	0.6945	1.2	0.2922
61	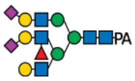	4.1	0.0002	2.3	0.0049	2.4	0.0245	3.7	0.0002
62	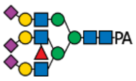	5.8	< 0.0001	2.5	0.0101	2.7	0.0019	3.3	0.0050
64	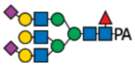	1.9	0.0010	1.8	< 0.0001	2.1	0.0681	1.7	0.0019
65	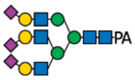	1.4	0.2898	1.7	0.0277	1.2	0.6945	1.1	0.3547
66	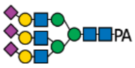	3.0	0.0122	2.0	0.0019	2.0	0.0084	2.0	0.0134
67	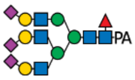	1.7	0.0112	1.9	0.0002	1.9	0.1537	1.2	0.1323
68	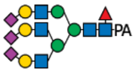	1.5	0.0011	1.4	0.0001	1.6	0.0112	1.1	0.2777
69	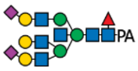	3.2	< 0.0001	1.8	0.0011	3.1	0.0381	2.2	0.0010
70	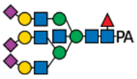	1.9	0.0008	1.6	0.0016	2.0	0.0112	1.2	0.1019

Table F. (continued)

Glycan #	Proposed Structures	Gastric cancer		Pancreatic cancer		Cholangiocacinoma		colorectal cancer	
		Fold change[b]	*p*-values[a]	Fold change[b]	*p*-values[a]	Fold change[b]	*p*-values[a]	Fold change[b]	*p*-values[a]
72	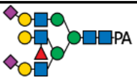	4.6	0.0002	2.3	0.0036	3.2	0.0112	3.7	0.0003
73	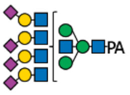	33.6	0.0041	4.1	0.0003	2.8	0.0530	2.8	0.0263
74	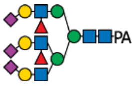	8.7	< 0.0001	2.7	0.0311	3.6	0.0043	4.5	0.0021
75	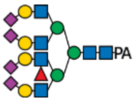	7.4	< 0.0001	2.7	0.0011	2.7	0.0006	3.4	0.0019
77	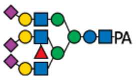	40.5	< 0.0001	15.4	0.0001	12.0	0.0002	20.9	< 0.0001
78	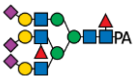	4.5	< 0.0001	2.6	0.0009	4.2	0.0043	3.4	0.0008
79	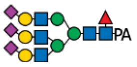	3.0	0.0001	2.0	< 0.0001	2.9	0.0307	2.0	0.0034
80	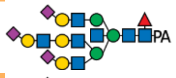	2.5	< 0.0001	1.7	0.0057	2.2	0.0307	1.8	0.0059
81	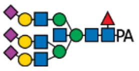	3.6	< 0.0001	2.1	0.0066	3.1	0.0191	2.2	0.0042
3N-LnM2A′	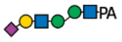	1.2	0.5733	1.1	0.3916	1.4	0.0307	0.9	0.3353
6N-LnM2A′	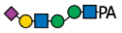	3.5	0.0002	1.8	0.0004	3.9	0.0002	1.5	0.0134
Ac-1	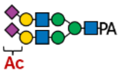	2.2	0.0041	1.5	0.0005	3.2	0.0006	3.2	0.0059
Ac-2	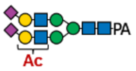	1.5	0.0904	1.3	0.0482	1.4	0.0381	1.2	0.1592
Ac-3	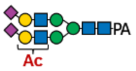	1.4	0.0886	1.2	0.0954	1.3	0.0568	1.2	0.2373
Ac-4	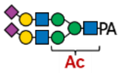	2.2	0.0018	1.6	0.0003	3.2	0.0003	3.1	0.0005
Ac-5	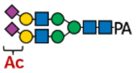	1.4	0.0961	1.3	0.0194	1.4	0.0153	1.3	0.0413
Ac-5b	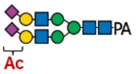	1.4	0.1124	1.2	0.0482	1.3	0.0245	1.2	0.1241

Related to Fig 6 and Fig G (in S2 File), the glycan levels of the groups of patients with gastric cancer (n=12), pancreatic cancer (n=10), cholangiocarcinoma (n=4) and colorectal cancer (n=15) and normal controls (n=21) are compared. In this table, the number and values of the glycans that meet simultaneously fold change ≥ 3.0 and p-value < 0.05 are labeled with an orange background.

a) The *p*-values were calculated by Mann−Whitney *U*-test.

b) The ratio of mean values of each cancer group to normal controls.

References for Supporting information

s1. Tanaka-Okamoto M, Hanzawa K, Murakami H, Mukai M, Miyamoto Y. Identification of β1-3 galactosylglucose-core free-glycans in human urine. Anal Biochem. 2021:114427. Epub 2021/10/25. doi: 10.1016/j.ab.2021.114427. PubMed PMID: 34688604.

s2. Hanzawa K, Tanaka-Okamoto M, Murakami H, Mukai M, Takahashi H, Omori T, et al. Investigation of acidic free-glycans in urine and their alteration in cancer. Glycobiology. 2021;31(4):391-409. Epub 2020/11/03. doi: 10.1093/glycob/cwaa100. PubMed PMID: 33135073; PubMed Central PMCID: PMCPMC8091460.

s3. Korekane H, Tsuji S, Noura S, Ohue M, Sasaki Y, Imaoka S, et al. Novel fucogangliosides found in human colon adenocarcinoma tissues by means of glycomic analysis. Anal Biochem. 2007;364(1):37-50. Epub 2007/03/14. doi: 10.1016/j.ab.2007.01.034. PubMed PMID: 17350584.
