# Supplementary material for: Increased levels of acidic free-N-glycans, including multi-antennary and fucosylated structures, in the urine of cancer patients
Source: PLoS One. 2022 Apr 12;17(4):e0266927. doi: 10.1371/journal.pone.0266927 (PMC9004742; doi:10.1371/journal.pone.0266927)
Supplement: S2 File — (PDF) [file pone.0266927.s002.pdf]

Supporting information (S2 File)

Increased levels of acidic free-*N*-glycans, including multi-antennary and fucosylated structures, in the urine of cancer patients

Ken Hanzawa[1], Miki Tanaka-Okamoto[1], Hiroko Murakami[1], Noriko Suzuki[2], Mikio Mukai[3], Hidenori Takahashi[4], Takeshi Omori[4], Kenji Ikezawa[5], Kazuyoshi Ohkawa[5], Masayuki Ohue[4], Shunji Natsuka[2], Yasuhide Miyamoto[1*]

[1] Department of Molecular Biology, Osaka International Cancer Institute, 3-1-69 Otemae, Chuo-ku, Osaka 541-8567, Japan

[2] Graduate School of Science and Technology, Niigata University, 8050 Ikarashi-nino-cho, Nishi-ku, Niigata, 950-2181, Japan

[3] Department of Medical Checkup, Osaka International Cancer Institute, 3-1-69 Otemae, Chuo-ku, Osaka 541-8567, Japan

[4] Department of Gastroenterological Surgery, Osaka International Cancer Institute, 3-1-69 Otemae, Chuo-ku, Osaka 541-8567, Japan

[5] Department of Hepatobiliary and Pancreatic Oncology, Osaka International Cancer Institute, 3-1-69 Otemae, Chuo-ku, Osaka 541-8567, Japan.

[*]Corresponding author
E-mail: miyamoto-ya@mc.pref.osaka.jp (YM)

Fig A. Standardization of elution positions of reversed phase HPLC (p2).

Fig B. HPLC fractionation of urinary glycans (p3).

Fig C. Structural analysis of an unusual hybrid-type free-*N*-glycan containing $Man_4GlcNAc_1$ and bi-antennary structures (p4).

Fig D. Structural analysis of a phosphorylated oligo-mannose-type free-*N*-glycan (p5).

Fig E. Structural analysis of tri-antennary free-*N*-glycans by 2D-HPLC mapping (p6, 7).

Fig F. Extracted ion chromatograms of reversed phase liquid chromatography / selected reaction monitoring for PA-labeled urinary free-glycans (p8, 9).

Fig G. Supporting data of levels of representative urinary free-glycans from SRM (p10, 11).

Fig H. Principal component analysis plots of glycan levels of cancer patients and normal controls (p12, 13).

Supporting Results. Principal component analysis plots of glycan levels (p13).

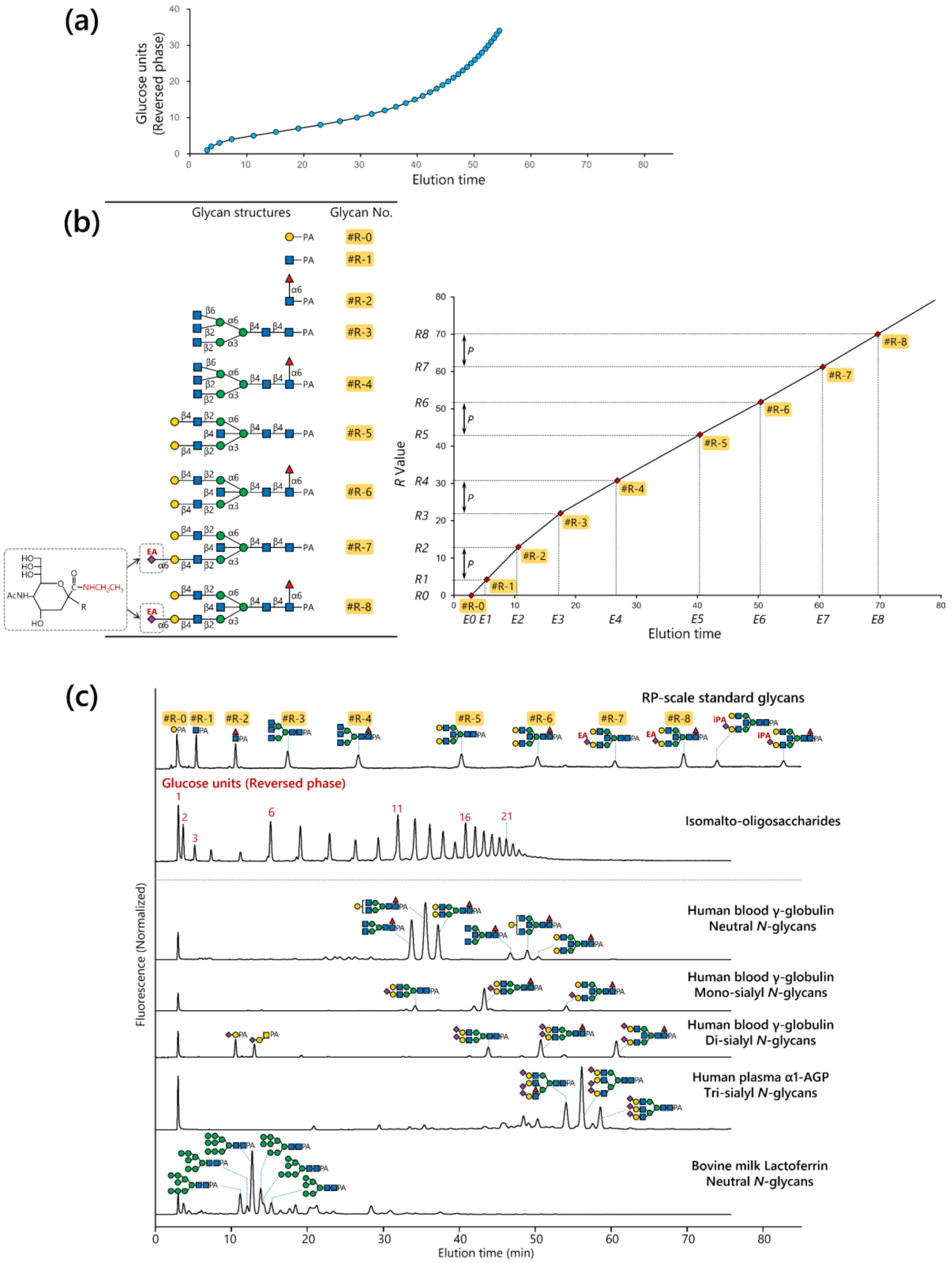

Fig A. Standardization of elution positions of PA-glycans on reversed phase HPLC. (a) Glucose units based on PA-labeled isomalto-oligosaccharides by reversed phase HPLC. (b) Standard PA-glycans for a modified reversed phase scale used in this study. *R* values were set to “0” for the elution position of PA-Gal (#R-0) and “70” for that of glycan #R-8. (c) Chromatographic comparison of the reversed phase standards, isomalto-oligosaccharides (GU1–22) and representative PA-glycans prepared from glycoproteins.

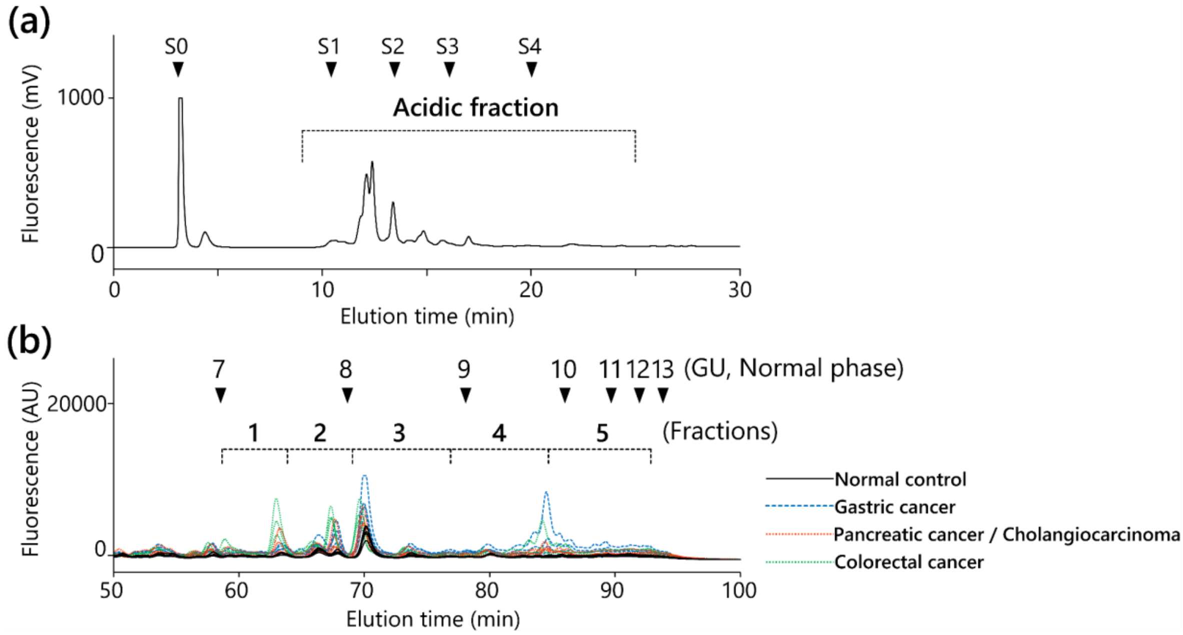

Fig B. HPLC fractionation of urinary glycans. The PA-labeled glycans were fractionated by anion-exchange HPLC and then normal phase (NP-) HPLC. (a) The elution profile of anion-exchange HPLC using a TSKgel DEAE-5PW column. Representative chromatogram of a normal control (obtained from N8) is shown. Arrowheads S0–S4 indicate the elution positions of standard PA-N-glycans with 0–4 neuraminic acids (NeuAc). (b) The elution profiles of NP- (amide-HILIC-mode) HPLC using a TSK-gel Amide-80 column. The acidic fraction from anion-exchange HPLC was fractionated in the range of GU 7–12.5. Representative overlaid chromatograms are shown from five normal controls (N5, N7, N8, N10 and N11), black line; four gastric cancer patients (G2, G8, G10 and G11), blue dotted line; two pancreatic cancer patients (P3 and P5) and two cholangiocarcinoma patients (B2 and B4), orange dotted line; three colorectal cancer patients (C6, C11 and C12), green dotted line.

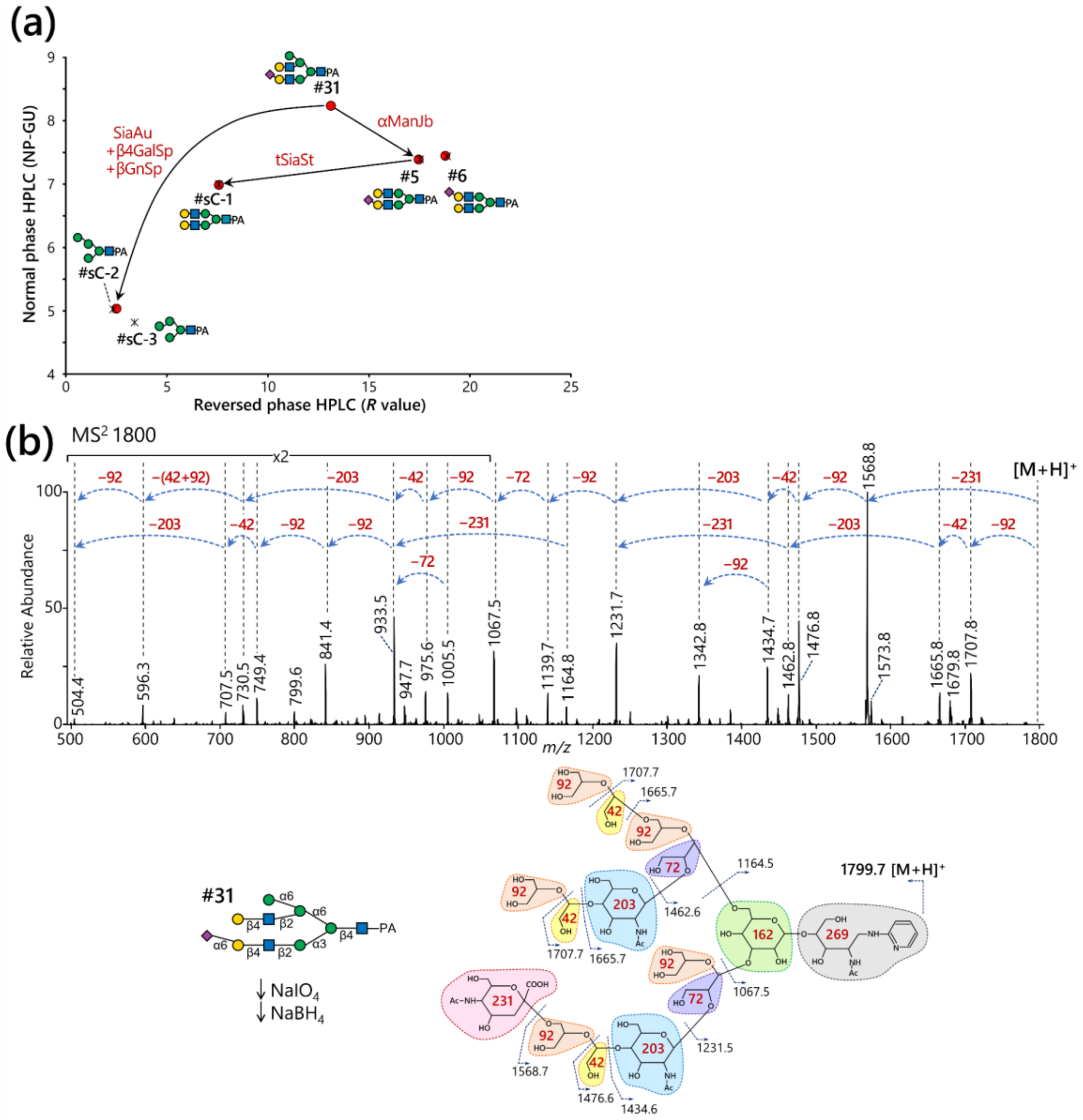

Fig C. Structural analysis of an unusual hybrid-type free-*N*-glycan containing $Man_4GlcNAc_1$ and bi-antennary structures. (a) Two-dimensional HPLC mapping analysis. The standardized elution positions are shown. Red circles and asterisks indicate the positions of the sample glycans and standard glycans, respectively. Solid arrows indicate shifts of the glycans following enzymatic digestion. Enzymes used in this analysis are indicated as follows: tSiaSt, neuraminidase for non-reducing terminal α2,3/6-linkages from *S. typhimurium*; neuraminidase with broad specificity from *A. ureafaciens*; β4GalSp, β1,4-galactosidase from *S. pneumoniae*; βGnSp, β-*N*-acetylglucosaminidase from *S. pneumoniae*; αManJb, α-mannosidase from jack bean. The hybrid-type glycan #31 was shifted to bi-antennary complex-type glycan #5 by digestion with αManJb, and then to #sC-1 with tSiaSt. On the other hand, #31 was shifted to #sC-2, an oligomannose-type glycan (Manα1-6Manα1-6(Manα1-3)Manβ1-4GlcNAc-PA) by sequential digestion with SiaAu, β4GalSp and β4GnSp. (b) MS^2 spectrum of the periodate-cleaved product of glycan #31 from the protonated ion at *m/z* 1800 $[M+H]^+$ (Hex6HexNAc3NeuAc1-PA +5×2H −6×CH_2O). This spectrum is consistent with the notion that glycan #31 possesses two β1,2-branches on both α1,3- and α1,6-mannose-arms.

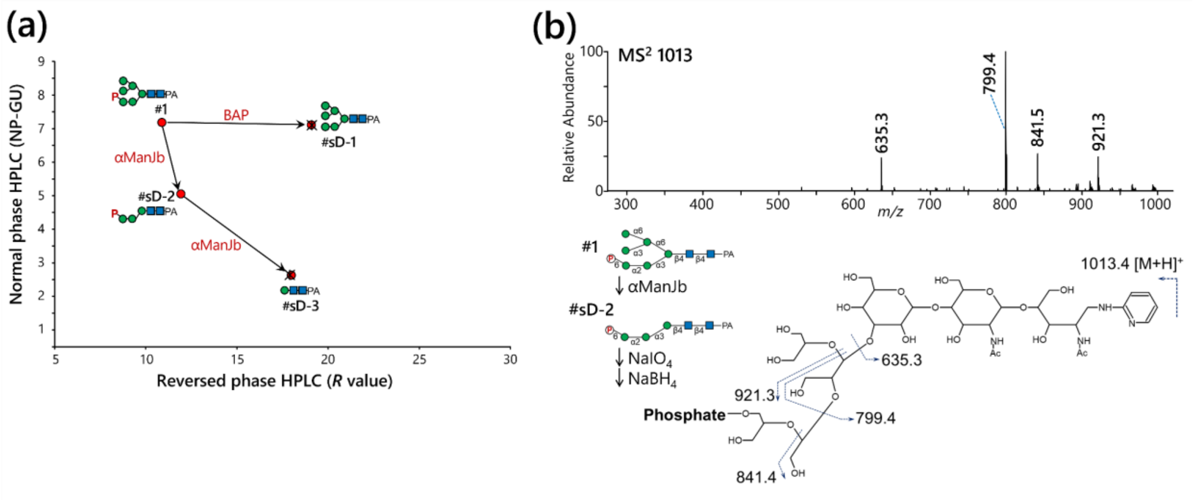

Fig D. Structural analysis of a phosphorylated oligo-mannose-type free-*N*-glycan. (a) Two-dimensional HPLC mapping analysis. The standardized elution positions are shown. Red circles and asterisks indicate the positions of the sample glycans and standard glycans, respectively. Solid arrows indicate shifts of the glycans following enzymatic digestion. The enzymes used are indicated as follows: BAP, alkaline phosphatase from *E. coli* C75; αManJb, α-mannosidase from jack bean. Glycan #1 (Figure 1, Line Fr 1) was shift to #s4-1, which corresponded to a standard $Man_6GlcNAc_2$ (#Gn2-23) by treatment with alkaline phosphatase. Also, α-mannosidase digestion of #1 resulted in #s4-2 ($Hex_3HexNAc_2Phosphate_1$-PA) and #s4-3, which corresponded to a standard $Man_1GlcNAc_2$ (#Gn2-3), by prolonged incubation. (b) MS^2 spectrum of the periodate-cleaved product of #s4-2 from the protonated ion at *m/z* 1013 $[M+H]^+$ ($Hex_3HexNAc_2Phosohate_1$-PA $+2\times2H$ $-2\times CH_2O$). The spectrum suggested that #1 contained a 6-phosphorylation at the α1,2-Man residue.

Fig E.

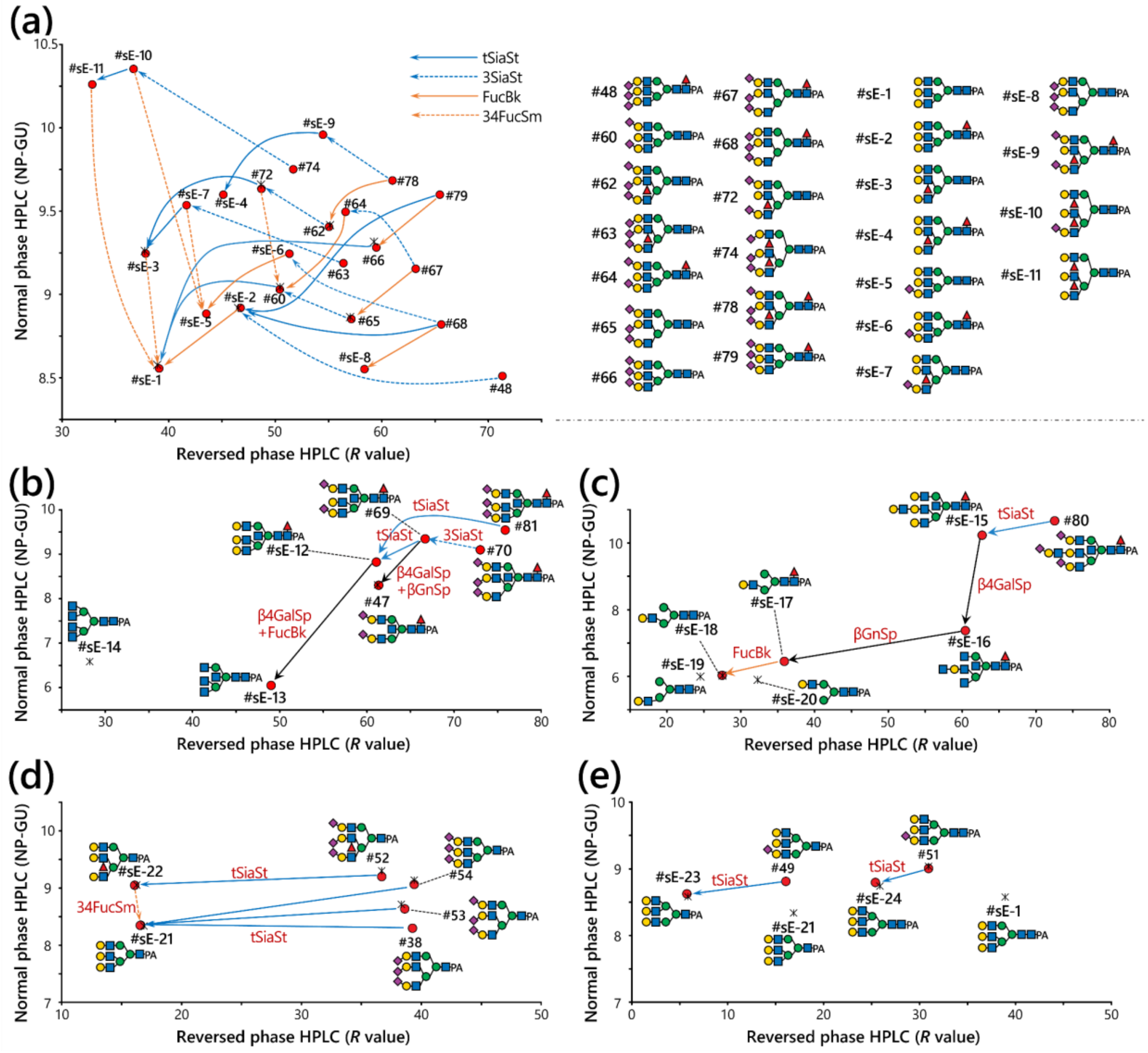

(a)
(b)
(c)
(d)
(e)
Normal phase HPLC (NP-GU)
Reversed phase HPLC (R value)
tSiaSt
3SiaSt
FucBk
34FucSm
β4GalSp
βGnSp
β4GalSp +βGnSp
β4GalSp +FucBk

Fig E. Structural analysis of tri-antennary free-*N*-glycans by 2D-HPLC mapping. The elution positions of PA-glycans on NP- and RP-HPLC were converted into NP-glucose units (GU) and *R*-values, respectively. The positions of sample glycans are indicated as red circles, and those of standard glycans are indicated as asterisks. Arrows indicate shifts of the elution positions of the glycans by glycosidases. The glycosidases used were indicated as follows: 3SiaSt, α-neuraminidase under the conditions for non-reducing terminal α2,3-linkages from *S. typhimurium*; tSiaSt, α-neuraminidase for non-reducing terminal α2,3/6-linkages from *S. typhimurium*; FucBk, α-fucosidase from bovine kidney, which act on the core-α1,6-fucose, but very slowly on the asialo-antennal α1,3-fucose; 34FucSm, α1,3/4-fucosidase from *Streptomyces* sp. 142; β4GalSp, β1,4-galactosidase from *S. pneumoniae*; βGnSp, β-*N*-acetylglucosaminidase from *S. pneumoniae*. Shifts following treatment with tSiaSt, 3SiaSt, FucBk and 34FucSm are indicated by blue solid arrows, blue dotted arrows, orange solid arrows and orange dotted arrows, respectively. (a) Representative mapping of sialyl tri-antennary glycans containing 2,4,2'-backbone (β1,4-branched) and Gn2-core with/without fucosylation. (b) Analysis of tri-antennary glycans with bisecting GlcNAc. Glycans #69, 70 and 81 shared the neutral backbone structure (#s5-12), and the di-sialyl glycan #69 was shifted to the di-sialyl bi-antennary glycan with bisecting GlcNAc (#47) by digestion with β1,4-galactosidase + β-*N*-acetylglucosaminidase. (c) Analysis of a LacNAc-extension of the β1,4-branch of glycan #80. The glycan was sequentially digested by α-neuraminidase, β1,4-galactosidase, β-*N*-acetylglucosaminidase and α-fucosidase, which generated a mono-antennary glycan containing a β1,4-branch (#s5-18). (d) Representative mapping of sialyl tri-antennary glycans containing 2,4,2'-backbone (β1,4-branched) and Gn1-core with/without fucosylation. (e) Representative mapping of mono-sialyl tri-antennary glycans containing 2,2',6'-backbone (β1,6-branched). Neuraminidase digests of the Gn1-type #49 and the Gn2-type #51 were shifted into the positions corresponding to 2,2',6'-branched neutral glycans.

Fig F.

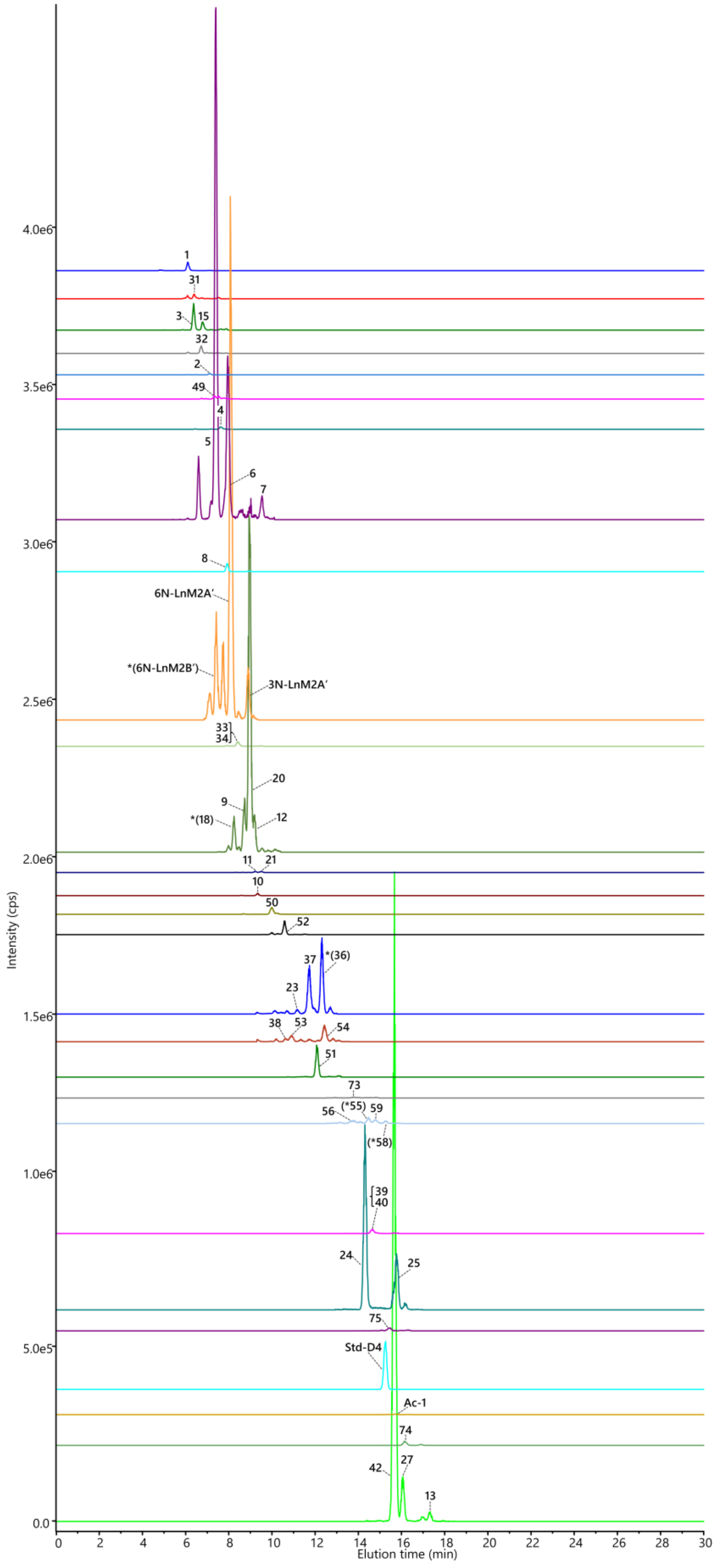
1
31
3
15
32
2
49
4
5
6
7
8
6N-LnM2A'
*(6N-LnM2B')
3N-LnM2A'
33
34
20
9
*(18)
12
11
21
10
50
52
37
*(36)
23
38
53
54
51
73
56
(*55)
59
(*58)
39
40
24
25
75
Std-D4
Ac-1
74
42
27
13
4.0e6
3.5e6
3.0e6
2.5e6
2.0e6
1.5e6
1.0e6
5.0e5
0.0
Intensity (cps)
0 2 4 6 8 10 12 14 16 18 20 22 24 26 28 30
Elution time (min)

Fig F. (continued)

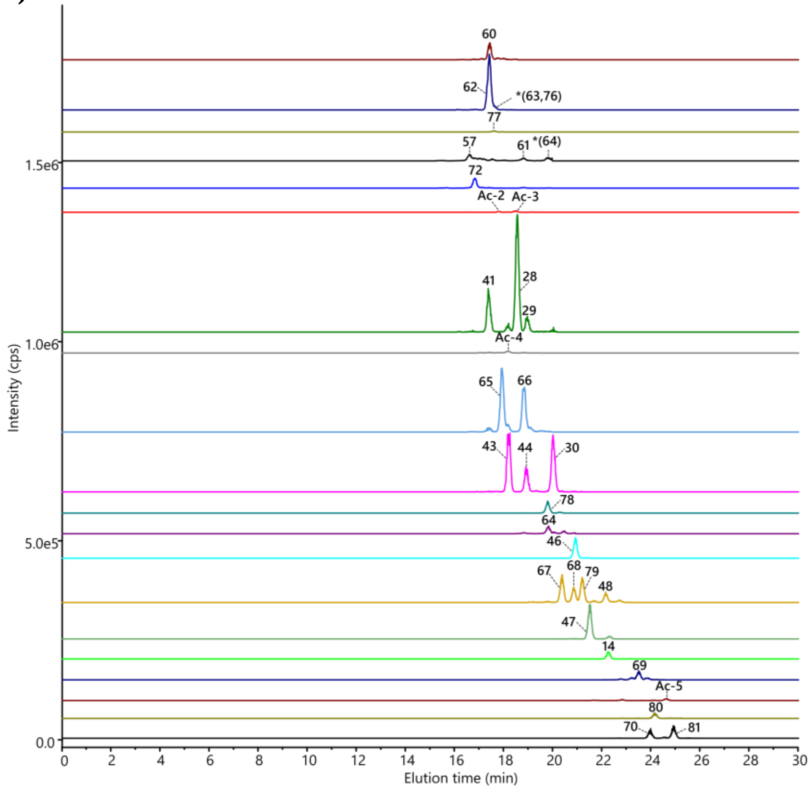

Fig F. Extracted ion chromatograms of reversed phase liquid chromatography / selected reaction monitoring for PA-labeled urinary free-glycans. Representative chromatograms of the quality control (QC) sample are shown. The sample amount corresponded to 40 µg of creatinine of the urine sample. Peaks are labeled with the numbers of the corresponding glycans. Peaks with asterisked number were not subjected to quantification.

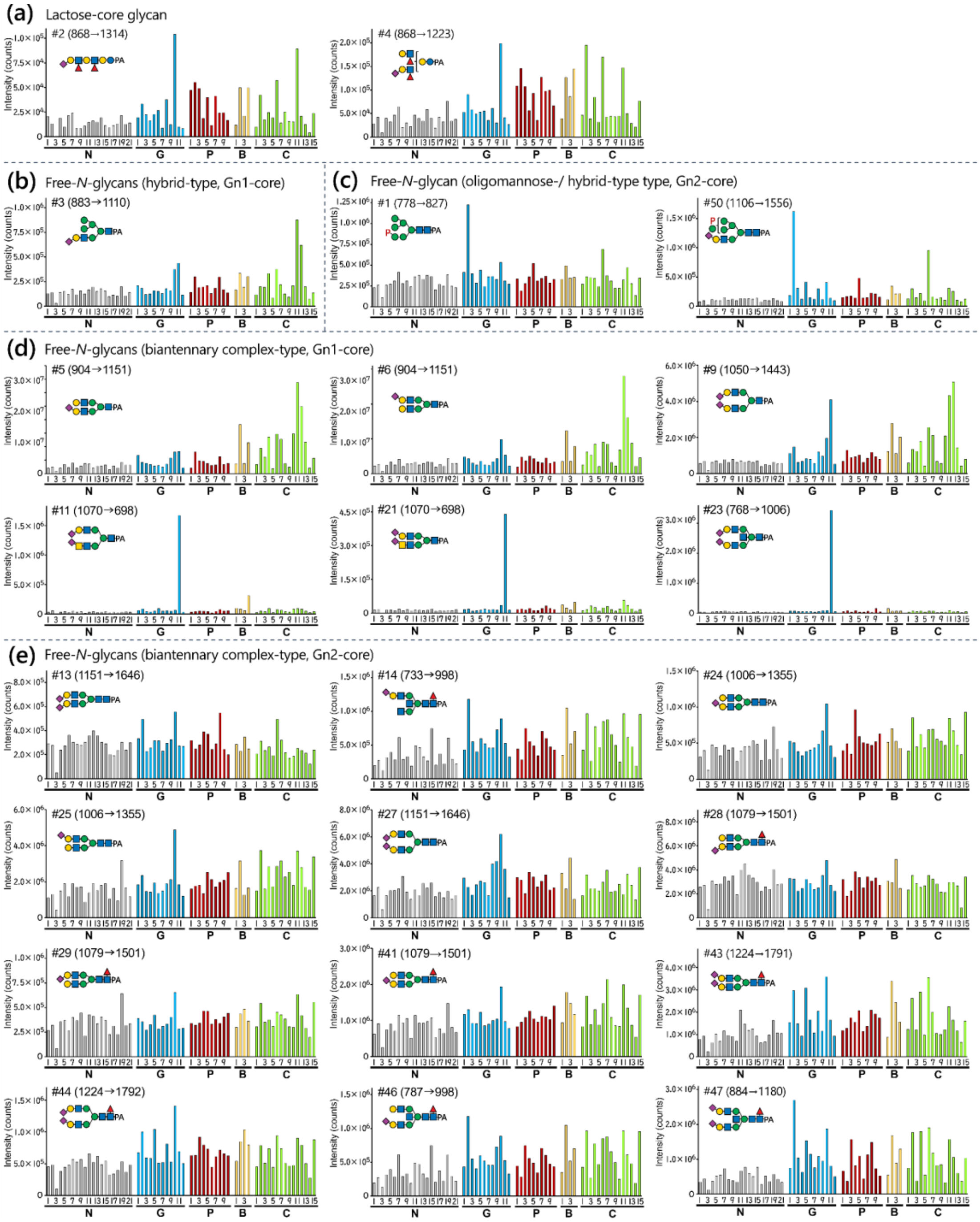

Fig G. Supporting data of levels of representative urinary free-glycans from SRM. The peak areas in the extracted ion chromatograms of SRM measurements are shown. The levels of the glycans are indicated by bars as follows: normal controls (N1–N21), gray; gastric cancer patients (G1–G12), blue; pancreatic cancer patients (P1–P10), red; cholangiocarcinoma patients (B1–B4), yellow; colorectal cancer patients (C1–C15), light green. In each glycan panel, glycan number and proposed structure are indicated. The mass values of Q1 → Q3 (*m/z*) are indicated in parentheses. (a) Lactose-core glycan #2 and 4. (b) Hybrid-type, Gn1-core free-*N*-glycan #3. (c) Oligomannose-/Hybrid-type, Gn2-core free-*N*-glycan #1 and 50. (d) Biantennary complex-type, Gn1-core free-*N*-glycans #5, 6, 9, 11, 21 and 23. (e) Biantennary complex-type, Gn2-core free-*N*-glycans # 13, 14, 24, 25, 27–29, 41, 43, 44, 46 and 47. (f) Tri-/tetra-antennary complex-type, Gn1-core free-*N*-glycans #38, 54, 56, 59 and 73. (g) Tri-/tetra-antennary complex-type, Gn2-core free-*N*-glycans #48, 57, 60, 61, 64, 67–70, 72 and 78–80. (h) Other free-*N*-glycans measured: a standard glycan (#D4-std), additive for performance checking of HPLC and MS; $Man_2GlcNAc_1$-core #6N-LnM2A' and 3N-LnM2A'; *O*-acetylated glycans Ac-1, 2, 4 and 5.

Fig G. (continued)

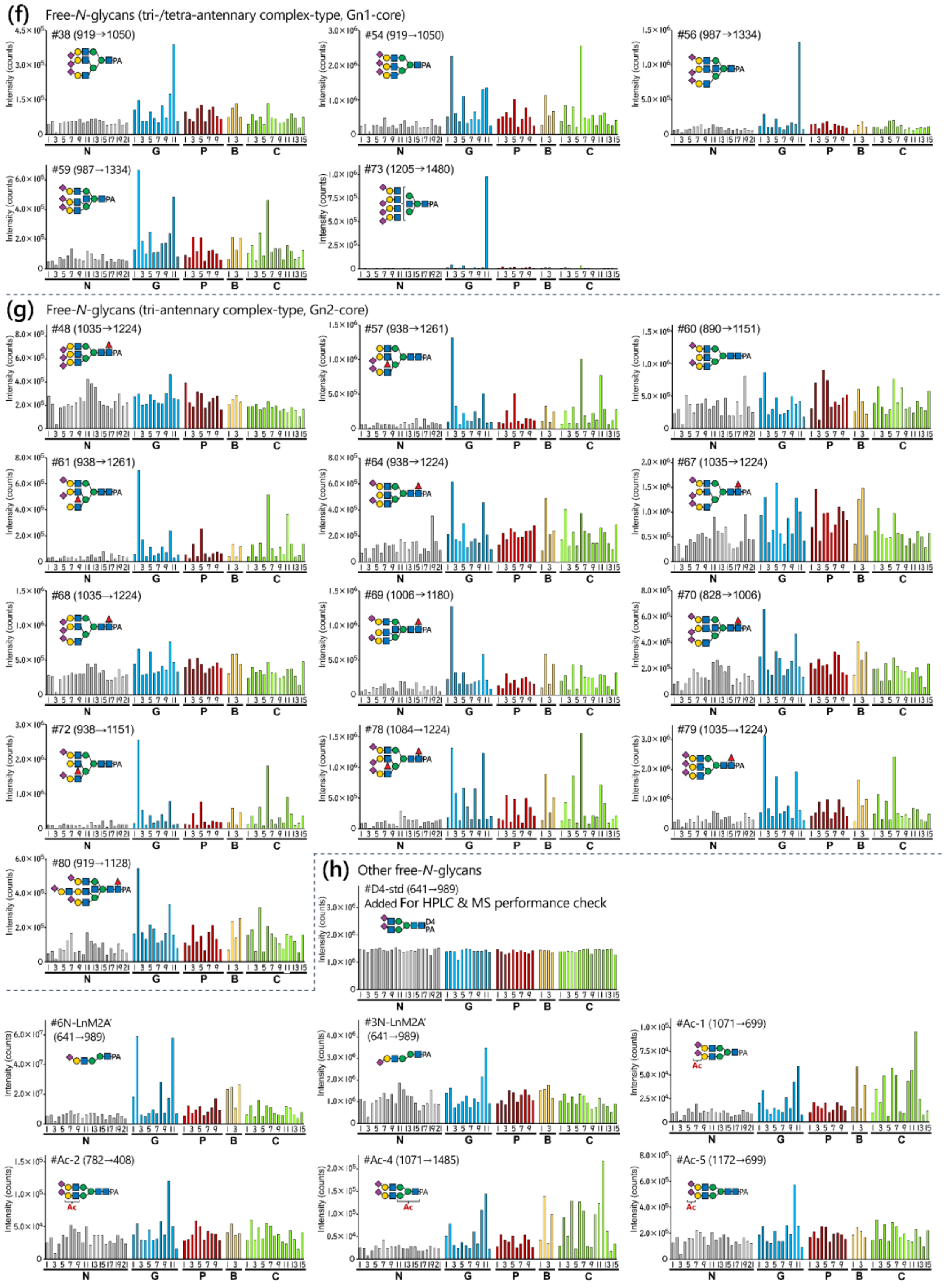

Fig H.

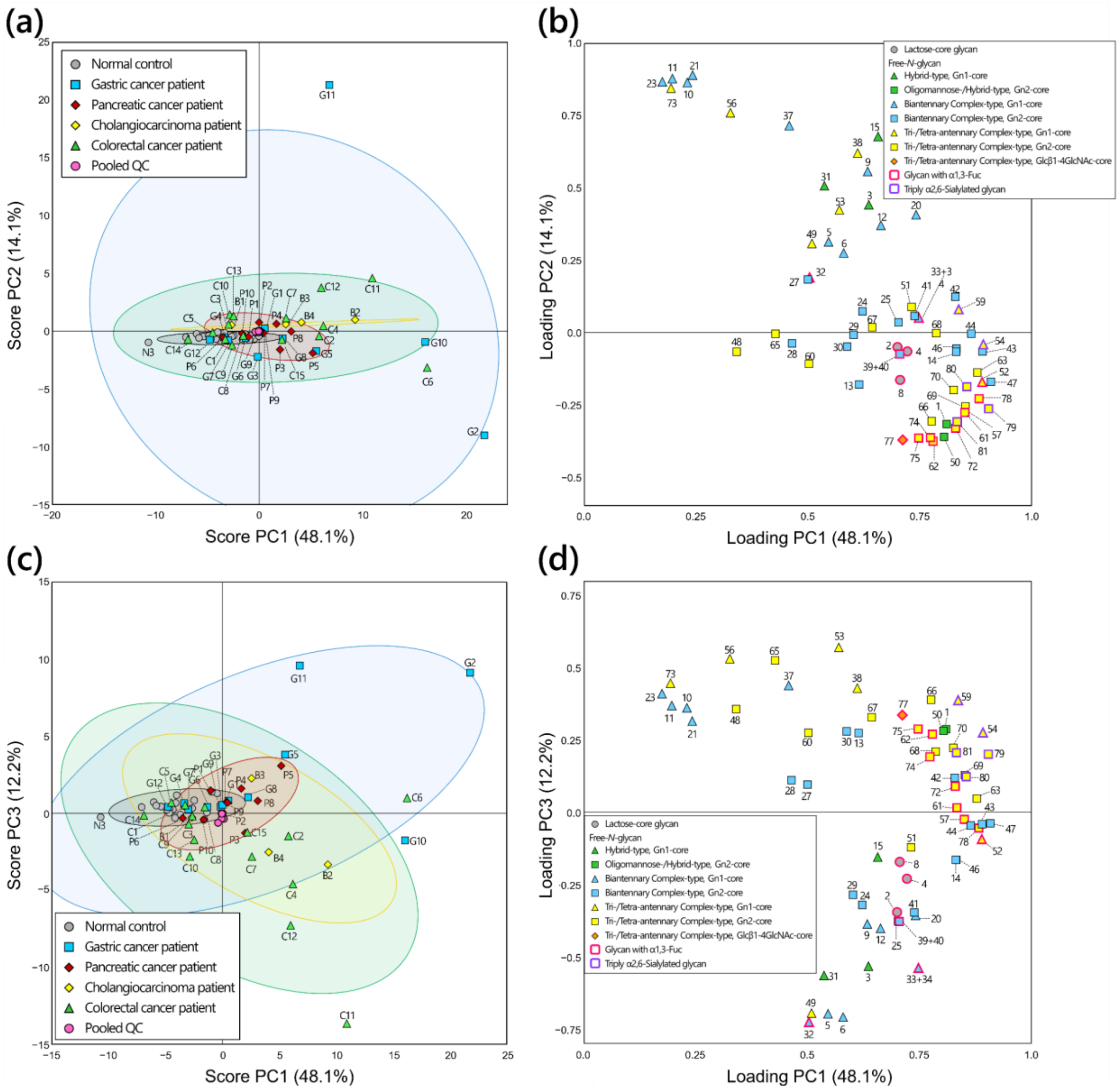

(a)
Normal control
Gastric cancer patient
Pancreatic cancer patient
Cholangiocarcinoma patient
Colorectal cancer patient
Pooled QC
Score PC2 (14.1%)
Score PC1 (48.1%)
(b)
Lactose-core glycan
Free-N-glycan
Hybrid-type, Gn1-core
Oligomannose-/Hybrid-type, Gn2-core
Biantennary Complex-type, Gn1-core
Biantennary Complex-type, Gn2-core
Tri-/Tetra-antennary Complex-type, Gn1-core
Tri-/Tetra-antennary Complex-type, Gn2-core
Tri-/Tetra-antennary Complex-type, Glcβ1-4GlcNAc-core
Glycan with α1,3-Fuc
Triply α2,6-Sialylated glycan
Loading PC2 (14.1%)
Loading PC1 (48.1%)
(c)
Score PC3 (12.2%)
Score PC1 (48.1%)
Normal control
Gastric cancer patient
Pancreatic cancer patient
Cholangiocarcinoma patient
Colorectal cancer patient
Pooled QC
(d)
Loading PC3 (12.2%)
Loading PC1 (48.1%)
Lactose-core glycan
Free-N-glycan
Hybrid-type, Gn1-core
Oligomannose-/Hybrid-type, Gn2-core
Biantennary Complex-type, Gn1-core
Biantennary Complex-type, Gn2-core
Tri-/Tetra-antennary Complex-type, Gn1-core
Tri-/Tetra-antennary Complex-type, Gn2-core
Tri-/Tetra-antennary Complex-type, Glcβ1-4GlcNAc-core
Glycan with α1,3-Fuc
Triply α2,6-Sialylated glycan

Fig H. Principal component analysis plots of glycan levels of cancer patients and normal controls. Principal component analysis (PCA) was performed using the data of glycan levels obtained from SRM (Fig 6 and Fig G in S2 File) in MetaboAnalyst 5.0 software. Plots were generated by Microsoft Excel for detailed labeling of the symbols. (a) Score plot of PC1 to PC2. (b) Loading plot of PC1 to PC2. (c) Score plot of PC1 to PC3. (d) Loading plot of PC1 to PC3. (a, c) Symbols indicate the cases as follows: gray circle, normal control; blue square, gastric cancer patient; red diamond, pancreatic cancer patient; yellow diamond, cholangiocarcinoma patient; green triangle, colorectal cancer patient; pink circle, pooled QC sample. The ellipses correspond to 95% confidence region of each group. (b, d) The glycans are plotted as symbols based on their structures, as follows; blue circle, lactose-core glycan; green triangle, hybrid-type Gn1-core free-*N*-glycan; green square, oligomannose-/hybrid-type Gn2-core free-*N*-glycan; blue triangle, bi-antennary complex-type Gn1-core free-*N*-glycan; blue square, bi-antennary complex-type Gn2-core free-*N*-glycan; yellow triangle, tri-/tetra-antennary complex-type Gn1-core free-*N*-glycan; yellow square, tri-/tetra-antennary complex-type Gn2-core free-*N*-glycan; symbol with red rim, glycan with α1,3-fucosylation; symbol with purple rim, tri-antennary glycan with triple α2,6-sialylation.

Supporting Results (Principal component analysis plots of glycan levels)

Data at the level of the glycans identified in this study and subjected to SRM were used. The PC1-PC2 and PC1-PC3 score plots achieved a separation, albeit partial, between normal controls and cancer patients (Fig H-a, H-c). Cancer patients with no apparent change in glycan levels were plotted mixed with normal controls. The QC samples (QC1–7) were tightly clustered, confirming the stability of the measurements. The PC1 (48.1%) axis seemed to indicate the overall alteration of glycan levels associated with cancer. The PC2 (14.1%) axis seemed to reflect both the reducing terminal core structure and the non-reducing terminal modification of the glycans (Fig H-a). In the PC1-PC2 loading plot, most of the free-*N*-glycans containing Gn1-core were located upward and those containing Gn2-core were located downward (Fig H-b). Most of the glycans with α1,3-fucosylation or triple α2,6-sialylation were plotted on the lower right, probably reflecting a similar increasing trend in some cancer patients. The extreme elevation of Gn1-core with sialyl LacdiNAc or bisecting GlcNAc in only one gastric cancer patient, G11, seemed to reflect the clustering of these glycans. The PC3 (12.2%) axis showed a tendency to separate some gastric cancer patients upward and colorectal cancer patients downward according to the PC1-PC3 score plot (Fig H-c). Referring to the PC1-PC3 loading plot (Fig H-d), Gn1-core glycans with sialyl LacdiNAc or bisecting GlcNAc, glycans with α1,3-fucosylation or triple α2,6-sialylation, in addition to some Gn1 and Gn2-type 2,4,2'-tri-antennary glycans with slight elevated levels, seemed to contribute to the isolation of gastric cancer. Lactose-core glycans, bi-antennary glycans, and Gn1-type bi-antennary and hybrid type glycans, in addition to some of the Gn1-type 2,2',6'-tri-antennary glycans, with small alterations in their levels, seemed to contribute to the isolation of the colorectal cancer samples by PCA.
